# Genome of *Leptomonas pyrrhocoris*: a high-quality reference for monoxenous trypanosomatids and new insights into evolution of *Leishmania*

**DOI:** 10.1038/srep23704

**Published:** 2016-03-29

**Authors:** Pavel Flegontov, Anzhelika Butenko, Sergei Firsov, Natalya Kraeva, Marek Eliáš, Mark C. Field, Dmitry Filatov, Olga Flegontova, Evgeny S. Gerasimov, Jana Hlaváčová, Aygul Ishemgulova, Andrew P. Jackson, Steve Kelly, Alexei Y. Kostygov, Maria D. Logacheva, Dmitri A. Maslov, Fred R. Opperdoes, Amanda O’Reilly, Jovana Sádlová, Tereza Ševčíková, Divya Venkatesh, Čestmír Vlček, Petr Volf, Jan Votýpka, Kristína Záhonová, Vyacheslav Yurchenko, Julius Lukeš

**Affiliations:** 1 Biology Centre, Institute of Parasitology, Czech Academy of Sciences, 370 05 České Budějovice (Budweis), Czech Republic; 2 Life Science Research Centre, Faculty of Science, University of Ostrava, 710 00 Ostrava, Czech Republic; 3 Institute for Information Transmission Problems, Russian Academy of Sciences, 127051, Moscow, Russia; 4 Institute of Environmental Technologies, Faculty of Science, University of Ostrava, 710 00 Ostrava, Czech Republic; 5 School of Life Sciences, University of Dundee, Dundee, DD1 5EH, UK; 6 Department of Plant Sciences, University of Oxford, Oxford, OX1 3RB, UK; 7 Belozersky Institute of Physico-Chemical Biology, M.V. Lomonosov Moscow State University, 119991, Moscow, Russia; 8 Department of Biology, M.V. Lomonosov Moscow State University, 119991, Moscow, Russia; 9 Department of Parasitology, Faculty of Science, Charles University, 128 44 Prague, Czech Republic; 10 Department of Infection Biology, Institute of Infection and Global Health, University of Liverpool, Liverpool, L3 5RF, UK; 11 Department of Biology; University of California at Riverside, Riverside, 92521, CA USA; 12 de Duve Institute, Université Catholique de Louvain, 1200, Brussels, Belgium; 13 Institute of Molecular Genetics, Czech Academy of Sciences, 142 20 Prague, Czech Republic; 14 Faculty of Science, University of South Bohemia, 370 05 České Budějovice (Budweis), Czech Republic; 15 Canadian Institute for Advanced Research, Toronto, ON M5G 1Z8, Canada

## Abstract

Many high-quality genomes are available for dixenous (two hosts) trypanosomatid species of the genera *Trypanosoma*, *Leishmania*, and *Phytomonas*, but only fragmentary information is available for monoxenous (single-host) trypanosomatids. In trypanosomatids, monoxeny is ancestral to dixeny, thus it is anticipated that the genome sequences of the key monoxenous parasites will be instrumental for both understanding the origin of parasitism and the evolution of dixeny. Here, we present a high-quality genome for *Leptomonas pyrrhocoris*, which is closely related to the dixenous genus *Leishmania*. The *L. pyrrhocoris* genome (30.4 Mbp in 60 scaffolds) encodes 10,148 genes. Using the *L. pyrrhocoris* genome, we pinpointed genes gained in *Leishmania*. Among those genes, 20 genes with unknown function had expression patterns in the *Leishmania mexicana* life cycle suggesting their involvement in virulence. By combining differential expression data for *L. mexicana*, *L*. *major* and *Leptomonas seymouri*, we have identified several additional proteins potentially involved in virulence, including SpoU methylase and U3 small nucleolar ribonucleoprotein IMP3. The population genetics of *L. pyrrhocoris* was also addressed by sequencing thirteen strains of different geographic origin, allowing the identification of 1,318 genes under positive selection. This set of genes was significantly enriched in components of the cytoskeleton and the flagellum.

The trypanosomatids (family Trypanosomatidae, class Kinetoplastea) are a group of protists distinguished by a highly specialized mitochondrion and a prominent mitochondrial genome, the kinetoplast. All known trypanosomatids are exclusively parasitic and found primarily in insects^1^. Three lineages have developed a dixenous life cycle that involves a secondary host, which can either be a vertebrate or a vascular plant^2^. Members of the genera *Trypanosoma* and *Leishmania* cause serious diseases in animals and humans. Species of the genus *Leptomonas* have a simpler monoxenous (one-host) life cycle, which is confined to insects, where they almost exclusively occupy the intestinal tract^3^. Monoxenous trypanosomatids are transmitted from one insect to another *via* food sharing, coprophagy and/or cannibalism^4^. Together with *Crithidia*, *Lotmaria*, and *Leishmania*, most nominal *Leptomonas* species belong to the recently established subfamily Leishmaniinae^5^. Such a phylogenetic position implies that acquisition of the *Leishmania* dixenous life style occurred within this clade. We propose that direct comparison of the genomes of *Leishmania* and *Leptomonas* will reveal specific genes and/or pathways driving evolution of dixeny within this group^1^.

The border between monoxenous and dixenous species is not impenetrable. Some monoxenous trypanosomatids can withstand the high temperature encountered in warm-blooded vertebrates^6^,^7^,^8^. It has been proposed recently that some monoxenous species are facultatively dixenous if conditions permit and favor such an alteration of their life cycle^9^,^10^. One such requirement is the absence of an efficient immunological mechanism for clearing the parasite from the potential host. Indeed, several monoxenous species have been found co-infecting vertebrates along with HIV, *Trypanosoma cruzi*, or *Leishmania* spp^9^,^10^,^11^.

Our knowledge of protist genomes is skewed towards parasitic species, with trypanosomatids being one of the most prominent cases^12^. However, this applies exclusively to dixenous trypanosomatids, as they encompass all medically and veterinary relevant species^13^. Hence, dozens of species and strains of *Leishmania* and *Trypanosoma* have been subjected to genome-wide and transcriptome analyses by next-generation sequencing (reviewed in^12^,^14^,^15^). The genomes of all studied *Leishmania* species are fairly similar and range in size from 27 Mb in *Leishmania enriettii* (W. Warren and S. Beverley, pers. commun.) to 33 Mb in *L. major* and *L. infantum*^16^,^17^. The number of chromosomes ranges between 34 and 36, and the overall genome synteny for these species is remarkably conserved. Genome sizes of characterized trypanosomes vary from 22 Mb in *T. b. gambiense* to 47.5 Mb in *T. vivax*^18^,^19^,^20^. The genomes of *Leishmania* and *Trypanosoma* are densely packed with almost invariably intron-less genes organized into long polycistronic clusters^21^.

The only four monoxenous sequenced genomes, albeit in draft quality, are from *Lotmaria passim* (published as *Crithidia mellificae* strain SF) parasitizing honey bees (scaffold N50 = 32 kb), *Leptomonas seymouri* isolated from the cotton stainer (N50 = 70.6 kb), and two endosymbiont-bearing species, *Angomonas deanei* and *Strigomonas culicis* described from hemipterid bugs and mosquitoes (N50 = 2.5 and 2.7 kb, respectively)^8^,^22^,^23^. An annotated genome sequence of *Crithidia fasciculata* encountered in dipteran flies is available in the TriTryp database (N50 = 920 kb, but with 427 unplaced contigs comprising nearly 8.7 Mb)^24^, in addition to contigs of an unidentified *Leptomonas* sp. (N50 = 3.4 kb), and *Herpetomonas muscarum* (N50 = 6.8 kb) in GenBank^25^,^26^. Preliminary analyses of the genomic content of these species revealed several genes specific for monoxenous lineages, but none has been examined in detail.

The model organism for the current study is *Leptomonas pyrrhocoris* (Zotta 1912). This species was isolated from the midgut of the firebug *Pyrrhocoris apterus* (Heteroptera: Pyrrhocoridae), but the range of suitable hosts is now known to embrace several species of the hemipterid family Pyrrhocoridae, including *Pyrrhocoris apterus*, *P. marginatus* and *Scantius aegyptius* in Europe and the Mediterranean, *Dysdercus poecilius* in China, and several *Dysdercus* spp. in the Neotropics and Africa. Our previous work suggested that *L. pyrrhocoris* originated in the Neotropics^3^. This is one a few truly globally distributed monoxenous trypanosomatids, the dissemination of which is facilitated by the omnipresence of its Pyrrhocoridae hosts in all regions studied thus far^2^.

The population genetics and speciation of monoxenous trypanosomatids remains poorly investigated, with information exclusively derived from dixenous *Trypanosoma* and *Leishmania* species^27^. The theory of preponderant clonal evolution states that, in natural populations of pathogens, recombination is infrequent and thus asexual binary division is the main means of reproduction^28^. Nevertheless, recombination has been documented in many Trypanosomatidae species, including *Leishmania major*, *L. donovani*, *L. guyanensis*, *L. infantum*, *Trypanosoma cruzi*, *T. brucei*, and *T. congolense*^29^,^30^,^31^. The relative contribution of these competing evolutionary forces remains to be scrutinized further, but some *Leishmania* hybrids were shown to have enhanced their transmission potential and fitness^32^,^33^. The only monoxenous species investigated in this regard are *Crithidia bombi* and *C. fasciculata*^34^,^35^,^36^. While recombination was not demonstrated in *C. fasciculata*, different strains of *C. bombi* regularly exchange genetic material, with occasional crossing during mixed infections^37^,^38^.

Here we describe the first complete, annotated genome of a monoxenous trypanosomatid parasite, *L. pyrrhocoris*. We also present population analysis of 13 isolates of *L. pyrrhocoris* collected worldwide, analysis of positive selection signatures in the protein-coding genes, and of their synteny, as compared to model trypanosomatids. We investigated gene family gains and losses in a phylogeny including dixenous species with published genomes and five monoxenous species (*L. pyrrhocoris*, *L. seymouri*, *C. fasciculata*, *Blechomonas ayalai*, *Paratrypanosoma confusum*), with a focus on genes gained in *Leishmania* and Leishmaniinae. In addition, we overlapped differential gene expression data for *Leishmania mexicana, Leishmania major*, and *Leptomonas seymouri* with gene family phyletic patterns and identified several novel proteins probably involved in *Leishmania* virulence.

## Results

### The genome of *L. pyrrhocoris*: general features

The *L. pyrrhocoris* genome was assembled almost to chromosome level (see Supplementary Methods for details) and contains 60 scaffolds (37× average coverage with 454 reads, maximum scaffold length 2,995,728 bp) with scaffold N50 of 910,096 bp and a total assembly length of approximately 30.4 Mb. High degree of synteny with other species (Supplementary Note 1, Supplementary Tables S1, S2 and Supplementary Figs S1–S3) indicates that *L. pyrrhocoris* genome was assembled correctly. Its current annotation contains 10,148 genes, of which 9,878 are protein-coding (plus rRNAs, tRNAs, snRNAs, snoRNAs, and other non-coding RNAs). This number falls within the range of previously annotated genomes of *Leishmania* and *Trypanosoma* (8,400 protein-coding genes for *L. major* Friedlin and 11,567 for *T. brucei* TREU927, TriTrypDB, release 25)^24^ and is significantly higher than that of the streamlined genomes of two plant-infecting *Phytomonas* spp., with 6,381 and 6,451 protein-coding genes^39^.

Many genes in the *L. pyrrhocoris* resulted from recent duplications. Using an E-value cut-off of 10^−10^ combined with a filter on percent identity (>70%) and the ratio of BLAST alignment length to query length (>0.8), the percentage of genes present as two or more homologous copies is estimated at ~14% (1,382 of all protein-coding genes). Percentages of duplicated protein-coding genes for *Phytomonas* sp. EM1, *Phytomonas* sp. HART1, *L. seymouri*, *L. major*, *T. brucei* TREU927, and *C. fasciculata* are 2.8%, 4.1%, 0.8%, 9.8%, 31.1%, and 33.0%, respectively. Notably, most groups of paralogs analyzed here are composed of exact duplicates with 99–100% (in *T. brucei* TREU927, *L. pyrrhocoris*, and *L. major*) or 95–100% in (*C. fasciculata*) identity at the amino acid level (Supplementary Fig. S4). The same picture was revealed by mapping gene family expansions on the tree of trypanosomatids: most striking expansions were mapped on the *C. fasciculata*, *T. brucei*, and *T. congolense* branches (Supplementary Fig. S5). Gene duplication represents a major genome evolution mechanism among trypanosomatids, given the general lack of transcription regulation in this group^40^. For example, 102 copies of a putative autophagy gene ATG8, 21 copies of a *Leishmania* GP46 surface-antigen homolog and 13 folate/biopterin transporters were found in the *L. pyrrhocoris* genome, probably reflecting the need for higher gene expression levels. On the other hand, certain lineages demonstrate a trend for streamlining of gene families, as exemplified by *Phytomonas* spp. (the percentage of duplicated genes in *Phytomonas* sp. EM1 was estimated at 2.8%).

### Metabolism and other functional modules in *L. pyrrhocoris*

**Figure 1:**
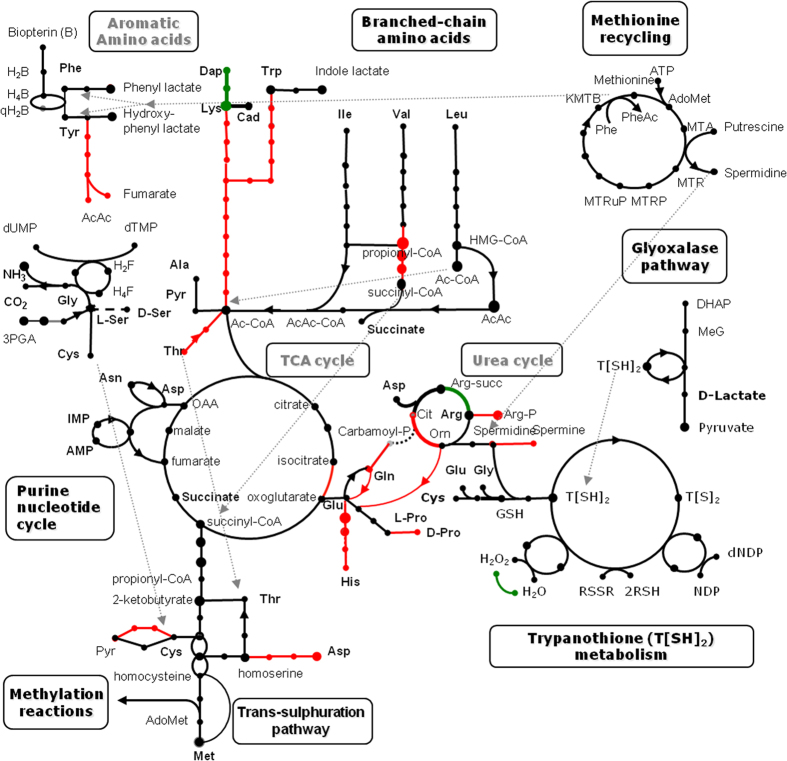
Schematic representation of *Leptomonas pyrrhocoris* amino-acid metabolism and some related pathways. Each dot represents a metabolite and each line represents the presence or absence of a predicted enzyme. Color coding: black, enzyme present; red, enzyme present in most free-living eukaryotic organisms, but lost in *L. pyrrhocoris*; green, enzyme present in *L. pyrrhocoris* and *Crithidia fasciculata*, but absent in *Leishmania*.

All predicted protein sequences of *L. pyrrhocoris* were compared with an in-house database of proteins representing over 550 metabolic enzymes from six previously sequenced trypanosomatid genomes as described previously^8^. The genome of *L. pyrrhocoris* contains genes coding for a fully functional mitochondrion with respiratory chain and functional glycosomes. A classic glycolytic pathway responsible for the metabolism of various exogenous sugars is partly located inside glycosomes (Supplementary Table S3). Carbohydrate metabolism is characterized by an incomplete aerobic oxidation because one of the classic mitochondrial TCA cycle enzymes, NAD-linked isocitrate dehydrogenase, has been replaced by an NADP-linked isoenzyme, making the cycle non-functional (Supplementary Note 2). This is a general feature of all trypanosomatids studied thus far^41^. However, all other TCA-cycle enzymes are encoded in the genome (Supplementary Table S4). The enzymes involved in the β-oxidation of fatty acids are also present. Similarly to *Leishmania* spp., *L. pyrrhocoris* is able to synthesize its own pyrimidines, but depends on a supply of external purines. Other metabolic features shared with *Leishmania* include the inability to oxidize aromatic amino acids and requirement for an external supply of most of the essential amino acids, cofactors, and vitamins for growth (Fig. 1, Supplementary Note 2, and Supplementary Table S5). A unique property amongst trypanosomatids, only shared with *C. fasciculata*, is the capacity to convert diaminopimelic acid, an amino acid of bacterial cell walls, into lysine. Diaminopimelate epimerase, one of the enzymes involved in this process, has apparently been acquired by the clade of *C. fasciculata* and *L. pyrrhocoris*, probably *via* horizontal gene transfer from bacteria, and then lost in *L. seymouri*. Oxidative stress protection in *L. pyrrhocoris*, *L. seymouri*, and *C. fasciculata* also differs from the other trypanosomatids analyzed thus far - in addition to the trypanothione system and many homologs of tryparedoxins and peroxiredoxins, the ancestor of these species has acquired a bacterial-type catalase by lateral gene transfer (Supplementary Table S6).

To illuminate specific features of *L. pyrrhocoris*, we also focused on other selected pathways and functional modules previously shown to be critical for the trypanosomatid biology, including membrane trafficking and small GTPases (Supplementary Note 3, Supplementary Tables S7, S8, and Supplementary Fig. S6), cell surface proteins (Supplementary Note 4 and Supplementary Figs S7–S9), and protein kinases (Supplementary Note 5 and Supplementary Table S9). Of particular interest is the presence of genes encoding Argonaute, Dicer1 and PIWI-like proteins (Supplementary Table S10). This suggests that *L. pyrrhocoris* is endowed with RNA interference capacity, similar to the African trypanosomes, *Leishmania braziliensis*, and *C. fasciculata*. Interestingly, genes for RNAi are lost in the close relative of *L. pyrrhocoris*, *L. seymouri*^8^.

### Gene gains and losses in trypanosomatids

**Figure 2:**
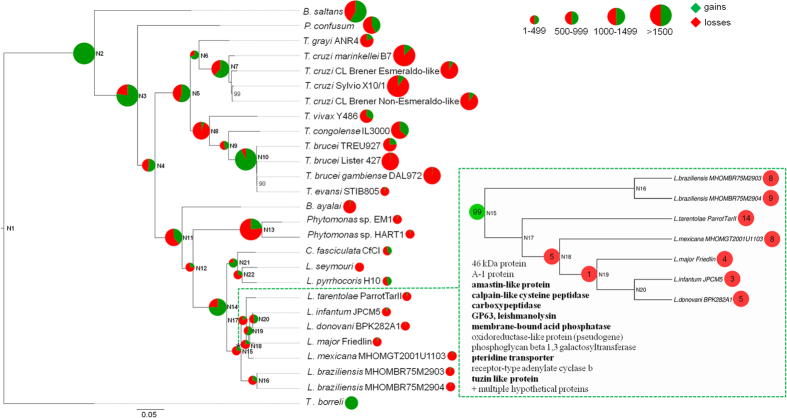
Gene family gains/losses mapped on the tree of kinetoplastids using Dollo parsimony algorithm. The maximum likelihood tree is based on the alignment of 57 conserved proteins and inferred using the LG + Г + F model and 1,000 bootstrap replicates. Only bootstrap support values lower than 100% are shown. The horizontal bar represents 0.05 substitutions per site. Gene gains dominate at the basal node of trypanosomatids, and at the basal nodes of Leishmaniinae, *Leptomonas/Crithidia*, American trypanosomes, *T. cruzi*, and *T. brucei*. The other internal nodes and leafs are either dominated by losses or have almost equal counts of gains and losses. An inset figure on the right depicts the losses for 99 OGs gained at the *Leishmania* node. Annotations for proteins within these OGs are shown at the bottom left corner of the inset. Annotations of the known proteins implicated in virulence are in bold.

For unraveling evolution of the dixenous life style in *Leishmania* we focused on lineage-specific gene family gains and losses in the sister lineages *Leptomonas*/*Crithidia* and *Leishmania*, forming the clade Leishmaniinae^5^. We performed OrthoMCL analysis (see Methods for details) on a dataset of 27 annotated trypanosomatid genomes (Supplementary Table S1). This resulted in 19,866 orthologous groups (OGs), 8,318 of which contained proteins of *L. pyrrhocoris*. Next, we mapped gene family (i.e., OG) gains and losses on the tree of trypanosomatids with Dollo and Wagner (to infer OG expansions/contractions) parsimony algorithms (Fig. 2, Supplementary Figs S5, S10, S11)^42^. Gene gains clearly dominate at the basal node of trypanosomatids, and at the basal nodes of Leishmaniinae, *Leptomonas/Crithidia*, American trypanosomes, *T. cruzi*, and *T. brucei*. The other internal nodes and leafs are either dominated by losses or have almost equal counts of gains and losses (Fig. 2; Supplementary Fig. S11).

The OG gains and losses at the Leishmaniinae, *Leishmania*, and *Leptomonas/Crithidia* nodes are of primary interest for identification of novel genes involved in *Leishmania* virulence (Fig. 2, Supplementary Note 6, and Supplementary Figs S12–S22). Using genome sequences of three monoxenous Leishmaniinae species (*L. pyrrhocoris*, *L. seymouri*, and *C. fasciculata*) as a robust reference, we have delineated a group of 99 OGs gained at the basal node of *Leishmania* (Fig. 2). This group of proteins includes several known virulence factors, but a great majority, 87 of 99 OGs, is represented by proteins of unknown function, which highlights the need of future gene-focused functional studies. However, in the next section we will use differential expression data to zoom in on a group of most promising virulence factor candidates among *Leishmania*-specific proteins. Functions of genes gained at the *Leishmania* and Leishmaniinae nodes and their possible implications for the evolution of dixenous parasitism are discussed in Supplementary Note 6.

### Identification of novel *Leishmania* virulence factor candidates

**Figure 3:**
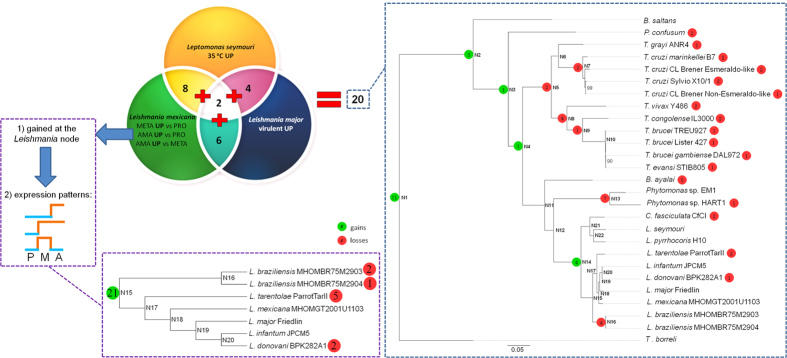
Approaches used for identification of novel *Leishmania* virulence factors. The Venn diagram represents two- and three-way intersections between differential expression datasets (**A**) (L*eptomonas seymouri* genes up-regulated at elevated temperature of 35 °C compared to 23 °C), (**B**) (genes up-regulated in a L*eishmania* major LV561 virulent isolate compared to an avirulent one), and (**C**) (differential expression data for *L. mexicana* developmental stages). Two phylogenetic trees of kinetoplastids with gene family gains/losses mapped using Dollo parsimony algorithm are shown. Gain and loss counts for 20 OGs obtained through overlapping the differential expression datasets are depicted in the tree on the right. The *Leishmania* cladogram at the bottom shows gains and losses for 21 OGs containing 40 *L. mexicana* genes gained at the *Leishmania* node and having differential expression patterns suggesting a potential role in virulence. *Leishmania* life cycle stages are abbreviated as follows: PRO or P, procyclics; META or M, metacyclics; AMA or A, amastigotes.

In our search for new candidates for *Leishmania* virulence factors we used OG gains/losses mapped on the tree of trypanosomatids and differential gene expression data generated by our team for *Leptomonas seymouri* (genes up-regulated at an elevated temperature^8^), *Leishmania major* LV561 (genes up-regulated in a virulent isolate compared to an avirulent one) and *L. mexicana* M379 (genes overexpressed at the virulent stages of the life cycle: metacyclic promastigotes and amastigotes) (Fig. 3, Supplementary Figs S23–S25). At least some of *Leishmania*-specific virulence factors are expected to be gained either at the Leishmaniinae node N14 or at the basal *Leishmania* node N15 (Fig. 2). In order to verify this assumption, we first focused on 47 proteins involved in virulence and confirmed by experimental studies (Supplementary Table S11), identified corresponding OGs and mapped their gains and losses on the tree (Supplementary Fig. S26). Five OGs were gained at the Leishmaniinae node, four at nodes within *Leishmania*, and the rest at more basal nodes of the tree.

Then, we took only those OGs gained at the *Leishmania* node which included *L. mexicana* M379 genes with relevant expression profiles in the life cycle, 21 OGs in total (Fig. 3, and Supplementary Table S12). The first group includes 35 genes (in 16 OGs) up-regulated in *L. mexicana* amastigotes as compared to the metacyclic and procyclic stages. A majority of these OGs in *L. mexicana* is annotated as proteins with unknown functions, with only one OG including amastin-like and A1 proteins (amastin is a known virulence-associated protein)^43^,^44^. The second group of interest contained two hypothetical proteins (two OGs) up-regulated in amastigotes and metacyclics in comparison to procyclics. Finally, the third group brought together three hypothetical proteins (three OGs) up-regulated in metacyclics only. Although, to the best of our knowledge, any functional data that might link these proteins to *Leishmania* virulence are lacking, the combination of two properties makes them a reasonably good starting group for future experimental studies: i/the gene families originated at the basal *Leishmania* node; ii/expression of the genes is up-regulated only at the virulent stages of the *Leishmania* life cycle. Excluding the OG annotated as amastin-like proteins leaves 20 novel OGs potentially involved in virulence.

By the second approach, we identified OG overlaps between the three differential expression datasets. As a result, 20 OGs were found to be shared by at least two datasets, and only two OGs were shared by all three datasets (Fig. 3 and Supplementary Table S13). Eleven of the 20 OGs had annotations of known virulence factors (Supplementary Table S13), while the remaining nine OGs represented proteins, the involvement of which in *Leishmania* virulence remains to be elucidated. We also inferred phyletic patterns for the OGs shared by the differential expression datasets (Fig. 3). The situation resembles that observed for the known *Leishmania* virulence factors (Supplementary Fig. S26) - a majority of the OGs were gained at the basal nodes, a high proportion of them was later lost in *Trypanosoma* and *Phytomonas* spp. and retained within the Leishmaniinae clade, and no OG was gained at the basal *Leishmania* node. Detailed information about four OGs gained at the Leishmaniinae node is presented in Table 1. Three of them have annotations of known virulence-associated proteins, while one of the OGs contains proteins with unknown function. Importantly, hypothetical proteins found within the latter OG have relevant differential expression patterns (up-regulated in *L. seymouri* at 35 °C and in *L. mexicana* amastigotes, Table 1). All proteins belonging to this OG have no known domains, are about 380 amino acids long, and are present in the *Leishmania* genomes (*L. donovani*, *L. infantum*, *L. major*, *L. mexicana*, and *L. tarentolae*) as single copies. They represent primary targets for gene ablation experiments in order to shed light on their function.

### Sequence polymorphism in the *L. pyrrhocoris* genome

Whole genome sequencing of clonal cultures of 13 *L. pyrrhocoris* lineages allowed us to study ploidy and the patterns of sequence variation across the genome. We sampled as widely as possible, including isolates from Europe, Central America and Africa (Supplementary Table S14). Despite this, the overall level of sequence variation was relatively modest, with an average number of sequence differences per nucleotide of 0.0058, comparable to *Arabidopsis thaliana* and about 5 times higher than in humans^45^,^46^. Total number of homozygous and heterozygous single nucleotide polymorphic (SNP) sites (inferred using *L. pyrrhocoris* H10 as a reference) varied from 18,654 in the F165 lineage to 219,139 in G58. In total, 907,015 SNP sites were identified. The majority of SNPs (892,221; 98.37%) were biallelic. The percentage of nonsynonimous SNPs ranged from 27% in the G58 lineage to 31% in P59, with 29% on average across all lineages. The average percentage of heterozygous sites was 2.6%, with the lowest value (1.1%) observed in the K06 lineage and the highest (6.2%) in CH278. Sequence variation was significantly lowered at non-synonymous, compared to synonymous sites in protein-coding genes (Supplementary Fig. S27), reflecting the action of purifying selection against deleterious amino acid replacements. The distribution of polymorphisms was uniform across the genome (Supplementary Fig. S28) with no apparent peaks or valleys typically seen around centromeres or telomeres in actively recombining genomes. Indeed, we found no evidence for the presence of recombination in our sequence polymorphism data using a coalescent-based approach^47^, indicating that this species is entirely clonal (see Methods for details). Interestingly, the *L. pyrrhocoris* genome harbors the same complement of meiosis-associated genes as *L. major* and *T. brucei* and also codes for homologs of proteins involved in gamete membrane fusion (HAP2/GCS1) and karyogamy (GEX1) in other eukaryotes^48^,^49^, suggesting that the cellular machinery for executing a (para)sexual cycle is present in this species, but it is not functional. An alternative explanation for the apparent lack of recombination may be the lack of genetic exchange between geographically remote populations of *L. pyrrhocoris*. Either way, the 13 *L. pyrrhocoris* isolates with sequenced genomes represent independent lineages that do not appear to have recombined with each other.

The ploidy of *L. pyrrhocoris* lineages was inferred using average read coverage for scaffolds under the assumption that most of them are diploid (which was supported by heterozygosity patterns). Twenty *L. pyrrhocoris* scaffolds (LpyrH10_01–02, 04–11, 13, 15, 17, 19, 21–22, 25, 31, 35, and 48), which constitute ~66% of the genome, were stably diploid in all 14 lineages investigated. Eleven scaffolds were found to be diploid in all lineages except one or two, while four scaffolds (LpyrH10_12, 29, 32, 42) are almost invariably tetraploid, scaffold 53 is almost invariably haploid, and five scaffolds (44, 45, 49, 56, 60) are polyploid or, more likely, represent unplaced repeats. Such ploidy pattern in *L. pyrrhocoris* strains is not surprising given the variable ploidy of *Leishmania* and *Trypanosoma* strains^50^,^51^,^52^.

### Adaptive evolution in the *L. pyrrhocoris* genome

To test whether any *L. pyrrhocoris* genes show evidence of molecular adaptation in the recent past (since the divergence of *L. pyrrhocoris* strains from their common ancestor), we employed a maximum likelihood phylogeny-based approach that is appropriate given the lack of recombination^53^. This approach is based on comparing the fit of nested models to data, with a simpler model (‘M7’) allowing only for purifying selection, and the more general model (‘M8’) accommodating both purifying and positive selection^54^. Likelihood ratio tests comparing these models demonstrated a significantly better fit of the M8 model for 1,318 *L. pyrrhocoris* genes, corresponding to 1,253 OGs (see Methods for details of the analysis). Thus, genes that putatively evolved under positive selection account for 13.3% of all protein-coding genes in *L. pyrrhocoris*. All three GO term categories (biological process, molecular function, and cell compartment) point to the fact that cytoskeleton- and flagellum-related functions occur more frequently among the positively selected genes as compared to the whole genome (Supplementary Table S15). Flagellum in trypanosomatids participates in cell mobility, cell division, influences cell size and organelle positioning, and may even function as a sensory platform for host-parasite interactions^55^.

Mapping the 1,253 OGs containing positively selected genes on the trypanosomatid phylogeny (Supplementary Fig. S29) revealed that the highest numbers of OGs were reconstructed to the basal nodes (N1–N3) and to the Leishmaniinae node N14: 493, 245, 143, and 162 OGs, respectively. Sixty one percent of the OGs containing genes under positive selection were gained within the studied phylogeny (i.e. cannot be reconstructed to the most basal node N1), while 44% were never lost, and 17% of OGs were never gained or lost within the studied phylogeny. A noticeable fraction of positively selected OGs (70 or 5.6%) was gained at the basal node of monoxenous species *C. fasciculata*, *L. pyrrhocoris*, and *L. seymouri*, and a substantial share was lost in both *Phytomonas* spp. (249 OGs, 19.9%) and in all *Leishmania* spp. (42 OGs, 3.4%). These observations highlight the functional significance of genes under positive selection for the monoxenous species of the *Leptomonas*/*Crithidia* clade. Indeed, the percentage of monoxenous-specific genes (i.e. genes gained at the *Crithidia/Leptomonas* node) under positive selection (16.8% or 70 out of 416, see Supplementary Table S16) is significantly higher than that across the genome (13.3%), with a 2 × 2 contingency test *p*-value of 0.0494. A great majority of those genes (52 of 70) have no functional annotation, and the remaining handful of genes are involved in carbohydrate and amino acid metabolism, glycolysis, cAMP signaling, protection against ROS and phenolic acid metabolism. These findings highlight the need for experimental studies to unravel functions of the proteins apparently important for the lifestyle of monoxenous trypanosomatids.

Five OGs containing positively selected genes were unique to *L. pyrrhocoris* (Supplementary Table S17). Two genes within these groups are annotated as proteins with unknown function; two other genes showed homology to *L. braziliensis* TATE transposable elements, and one gene was annotated as a putative surface antigen protein. For each of these genes positions of codons under positive selection were determined using the empirical Bayes method (Supplementary Table S17). The presence of mobile elements with a telomeric-repeat site specificity (TATE) resembling those found in the *L. braziliensis* genome correlates well with our finding that the *L. pyrrhocoris* genome encodes the full set of RNAi pathway genes with a potential role in control of transposable element mobilization^56^.

## Discussion

Kinetoplastid flagellates are a well-defined, clearly monophyletic lineage with a range of dramatically different life strategies, ranging from a free-living one, to obligatory endosymbiosis in an amoeba^57^ or complex parasitic life cycles involving invertebrate and vertebrate hosts or plants^1^. Due to their medical importance, virtually all attention was focused on trypanosomatids parasitizing humans, which resulted in high-quality nuclear genome sequences for *T. brucei*, *T. cruzi*, and *L. major*^58^,^59^,^60^. However, a decade later, when dozens of genomes of *Trypanosoma* and *Leishmania* have been published or are in progress, our information about the genomes of monoxenous trypanosomatids remains fragmentary. Since several genomes of draft quality available for these highly diverse parasites of insects^8^,^22^,^23^ are insufficient for a detailed comparison with dixenous genomes, we generated a high-quality genome sequence of a globally distributed parasite of firebugs, *L. pyrrhocoris*.

Both the size of the *Leptomonas* genome and its coding capacity are similar to the dixenous kins. The levels of synteny, ranging from 22% to 90% with *T. brucei* and *L. seymouri*, respectively, are not surprising and correlate with evolutionary distances between the compared trypanosomatids (Supplementary Note 1). The studied parasite possesses all core components of the RNAi pathway making *L. pyrrhocoris* particularly interesting from the perspective of functional studies.

The transition from the free-living style to parasitism in kinetoplastids was apparently followed by a massive gene loss, as suggested by comparing the ~18,943 gene content of *Bodo saltans*^61^, a representative of the free-living kinetoplastid group most closely related to parasitic trypanosomatids, with a mere 6,000 to 12,000 gene complement encountered in all sequenced trypanosomatids^24^,^39^,^62^. Detailed analysis has shown that in the process of streamlining its genome the ancestral trypanosomatid have lost genes that functioned in macromolecular digestion and assimilation, as well as many intracellular membrane pumps and ABC transporters, while expanded the families of membrane transporters for scavenging amino acids, nucleosides, and other metabolites from the host^61^. Our analysis also revealed gene losses in trypanosomatid amino acid metabolism (Fig. 1), as well as the loss of murein- and glycogen-degrading capabilities (Supplementary Note 2). A similarly drastic gene reduction occurred in the evolutionary history of apicomplexans, another highly successful and diverse parasitic lineage^63^, and genome reduction is thought to be a major evolutionary trend for parasites^64^.

Based on the patterns of gene family gains and losses in trypanosomatids (Fig. 2, Supplementary Fig. S11), we speculate that not only losses, but also massive gene gains at certain nodes, in particular the basal node of trypanosomatids, the Leishmaniinae node, and the *T. brucei* and *T. cruzi* nodes, have driven expansion into novel niches. This was followed by rather limited lineage-specific losses as a result of adaptation to specific hosts. *Phytomonas*, with its minimized gene repertoire, seems to be an extreme case of a prevailing OG loss trend^39^.

Emergence of novel gene families of transmembrane transporters and proteins participating in fatty acid biosynthesis and amino acid metabolism at the Leishmaniinae node clearly reflects continuing adaptation to the insect host, where sugars and amino acids serve as important energy substrates^65^. Previously reported presence of additional desaturases and elongases in *Leishmania* spp. and their absence in *T. brucei* and *T. cruzi*^66^ correlates with our OG analysis. Of importance is also the presence in *Leptomonas* spp. of at least a partial LPG (lipophosphoglycan) pathway, highly expressed in the *Leishmania* insect stages, indicating that both protists utilize similar molecules in their interaction with the insect hosts^67^,^68^. A detailed analysis of the amastin family in *L. pyrrhocoris* (Supplementary Note 4) further confirms that a substantial elaboration of amastin transmembrane glycoproteins occurred after the origin of *Leishmania*^43^.

Analyses of the protein machinery involved in vesicle trafficking in *L. pyrrhocoris* revealed a set of genes highly similar to the previously studied dixenous trypanosomatids (Supplementary Note 3, Supplementary Table S7). Notably, *L. pyrrhocoris* has retained all four adaptor complexes (AP-1 to AP-4) inherited from free-living kinetoplastids ancestors, whereas some of the complexes are missing in *Leishmania*, *Phytomonas* and African trypanosomes. However, *T. cruzi* also exhibits all four adaptor complexes, so their reduction is not directly linked to the transition from monoxeny to dixeny. The comparison of the small GTPase genes in different trypanosomatids (Supplementary Note 3, Supplementary Table S8) showed that gene loss is a much more significant trend in the evolution of this group of genes than innovation by gene duplications. Given the association of small GTPases with various basic cellular processes, this finding suggests the continuing simplification of the general cellular physiology in the trypanosomatid evolution. In terms of the number of ancestral small GTPases retained, *L. pyrrhocorris* stands between *T. cruzi* exhibiting a complete set and other dixenous trypanosomatids that show some secondary simplification of the ancestral small GTPase complement. Again, no straightforward correlation between the lifestyle and the complexity of this gene cohort is apparent. The analysis of small GTPases also revealed minimization of their ARF clade in *L. pyrrhocoris* (Supplementary Note 3). Overall, the studied trypanosomatid has an endomembrane system similar to that of its dixenous relatives with no evidence for massive remodeling accompanying the dixenous life style. As ARFs/ARLs tend to control vesicle coat assembly and other cytoskeletal-related functions, this may also reflect a decreased requirement for regulation of pathways accompanying switches between hosts. However, the stability of the Ras-like GTPase cohort between distantly related *L. pyrrhocoris* and *T. brucei*^69^ suggests that the system is unlikely to play a major role in life stage transition, and that these proteins rather play roles in general cellular physiology.

This study revealed that 38 OGs corresponding to known *Leishmania* virulence factors were gained at the basal nodes, 5 OGs were gained at the Leishmaniinae node, and only 4 OGs emerged at the nodes within the genus *Leishmania*. Notably, a high proportion of the putative virulence factors (from the 38 OG mentioned above) were lost in the streamlined genomes of *Phytomonas* spp. We consider two alternative explanations for the rather basal acquisition of most virulence factors, with only a small proportion gained at the Leishmaniinae node. Firstly, it is possible that not the genes themselves, but their coordinated expression is required for virulence. The second explanation relies on the fact that, due to the unavailability of monoxenous outgroups, previous studies lacked defined sets of OGs gained at the Leishmaniinae and *Leishmania* nodes and, consequently, these OGs were never a focus of *Leishmania* virulence factors studies. This could also explain the fact that none of the virulence factors in our dataset was gained at the *Leishmania* node. However, this does not imply that this node does not deserve attention in further studies specifically targeting *Leishmania* virulence factors. The inclusion of a high-quality monoxenous trypanosomatid genome and differential gene expression data in the OG analysis across all trypanosomatids has the added value of identifying additional virulence factors confined to *Leishmania*. Indeed, in this study we have identified 29 orthologous groups representing novel *Leishmania* virulence factor candidates.

## Methods

### Parasites and establishment of the clonal lines

*L. pyrrhocoris* isolate H10 isolated from the firebug, *Pyrrhocoris apterus*, in the Czech Republic^3^ was used for initial genome and transcriptome sequencing. Cultures were routinely maintained in Brain Heart Infusion (BHI) medium (Sigma-Aldrich, St. Louis, USA) supplemented with 10% Fetal Bovine Serum (Thermo Fisher Scientific, Waltham, USA), 50 units/ml of penicillin, 50 μg/ml of streptomycin (both from Sigma-Aldrich), and 10 μg/ml of hemin (Jena Bioscience GmbH, Jena, Germany) at +23 °C. Origin of other isolates used in this work is summarized in Supplementary Table S14. Two or three independent clonal lines for each of the of *L. pyrrhocoris* isolates 10VL, 121AL, 14BT, 25EC, 324RV, 329MV, CH278, F165, F19, G58, K06, P59, and SERG were established as described before^70^,^71^. In brief, parasite primary cultures were plated in multiple serial dilutions onto a 1% agar medium supplemented with BHI and antibiotics as described earlier^72^. The identities of clonal lines used in downstream analyses were confirmed by sequencing their spliced leader (SL) RNA gene repeats (see below). Cultures and clonal lines obtained were deposited in the collection of the Life Science Research Centre of the University of Ostrava and are available upon request.

SL RNA gene repeats were amplified using primers M167 and M168, and the PCR products were cloned and sequenced as described previously^73^,^74^,^75^. Obtained sequences were compared to published data^3^,^13^, and one clonal line from each isolate was selected for whole genome sequencing. SL sequences determined in the course of this work are deposited in GenBank under accession numbers KT012485–KT012505.

### Genome assembly and annotation, transcriptomic data processing and analysis

See Supplementary Methods.

### Gene family analysis using the OrthoMCL approach

Orthologous groups (OGs) for the *Leptomonas* proteins were inferred using the OrthoMCL v.2.0 software^76^. Full lists of annotated proteins for 23 trypanosomatid species were downloaded from the TriTrypDB v.7.0 and combined with newly annotated proteins from *L. pyrrhocoris* and three other trypanosomatid species: *L. seymouri*, *Blechomonas ayalai* and *Paratrypanosoma confusum* (Supplementary Table S1). The reference protein dataset was subjected to removal of poor quality proteins (based on sequence length and percent of in-frame stop codons), all vs. all BLAST search (E-value cut-off 10^−10^) and a clustering procedure implemented in the OrthoMCL algorithm.

Gene family gains and losses were mapped on the reference species tree using the COUNT software^42^. Gains and losses pattern was inferred using the Dollo and Wagner parsimony algorithms implemented in COUNT (with gain penalty value set to 3 for Wagner algorithm). Dollo parsimony allows a gene family to be gained only once, while Wagner parsimony allows multiple gains with a certain gain penalty and, in addition, inferring gene family expansions and contractions.

### Phylogenetic analysis

For the phylogenetic tree construction 644 OGs containing only one protein for each of 27 kinetoplastid species were taken. The protein sequences were aligned using MUSCLE^77^ with default parameters. The alistat script from the HMMER package v.3.1 was used to calculate average percent identity within each OG. Fifty seven OGs having average percent identity within the group >75% were used for constructing a multi-gene phylogeny. Protein alignments were individually trimmed in Gblocks v.0.91b with relaxed parameters (−b3 = 10 −b4 = 5 −b5 = h) and concatenated, producing an alignment of 22,544 characters. Maximum likelihood phylogenetic analysis was performed using RA × ML v.8.0.24 with the LG + Г phylogenetic model and with amino acid equilibrium frequencies inferred from the data^78^. One thousand bootstrap replicates and 200 iterations of the maximum likelihood algorithm were performed, and the best tree was visualized in FigTree v.1.4.1.

### Gene ontology analysis

Gene ontology (GO) annotation was performed for *L. pyrrhocoris* gene families gained/lost at certain nodes in the trypanosomatid tree using the Blast2GO PRO software^79^ with the following settings: BLAST E-value cut-off, 10^−10^; maximum number of hits per sequence, 100; with filtering of low complexity regions turned on. A BLASTP search was run against a local NCBI nr database (download date: 13.02.2015). Subsequently, mapping GO terms associated with BLAST hits onto query sequences was performed, followed by GO annotation, i.e. selection of most specific GO terms. Annotation was conducted with an E-value cut-off of 10^−10^ and an annotation cutoff of 55. GO term graphs were generated, and multi-level pie charts were created for each GO term category: cellular component, biological process, and molecular function. Combined graphs and pie charts were plotted with a sequence filter, i.e. a minimal number of sequences a GO node must contain in order to be displayed on the graph or chart, set to 5 in the case of gains/losses and to 10 for expansions/contractions. Analysis of GO terms enrichment including all the standard steps (BLAST search, GO terms mapping and annotation) was also performed for *L. pyrrhocoris* protein-coding genes showing signs of positive selection vs. all protein-coding genes using Fisher’s exact test with a *p*-value cut-off of 0.01.

### Identification of novel *Leishmania* virulence factor candidates

In order to identify novel proteins involved in *Leishmania* virulence, phyletic patterns for OGs were overlapped with three gene expression datasets generated with the Illumina HiSeq technology. The first dataset contained 189 genes of *L. seymouri* up-regulated in an axenic culture at an elevated temperature (35 °C) compared to 23 °C^8^. The second dataset was comprised of 53 genes up-regulated in a virulent isolate of *L. major* LV561 compared to its avirulent counterpart (our unpublished data). The third dataset included differential expression data for *L. mexicana* M379 developmental stages in axenic cultures (our unpublished data). This dataset, in turn, consisted of four subsets: i/genes up-regulated in metacyclics compared to procyclics (12 genes); ii/genes up-regulated in amastigotes compared to procyclics (433 genes); iii/genes up-regulated in metacyclics compared to amastigotes (713 genes); iv/genes up-regulated in amastigotes compared to metacyclics (358 genes). Subset iii/was omitted since it showed considerable overlap with subset ii/and probably included a lot of genes functioning in the insect host environment and not involved in virulence directly. In all datasets mentioned above, genes with expression fold change ≥1.5 and an FDR-corrected *p*-value ≤0.05 were chosen for further analyses. First, differential expression patterns of *L. mexicana* M379 genes gained at the *Leishmania* node were investigated. In brief, *L. mexicana* gene IDs were identified within OGs gained at the *Leishmania* node and genes (showing at least a 1.5-fold change in expression) with the following expression profiles in the life cycle were selected: i/up-regulated in amastigotes only; ii/up-regulated in metacyclics only; iii/up-regulated in amastigotes and metacyclics. Second, for each gene within the three differential expression datasets an OG number was inferred. All OGs overlapping between at least two expression datasets were identified. Both approaches combined resulted in identification of 29 OGs containing novel virulence factor candidates.

### Analysis of positive selection in *Leptomonas*

Paired-end 100 nt genomic reads for 13 clonal lines of *L. pyrrhocoris* (10VL, 121AL, 14BT, 25EC, 324RV, 329MV, CH278, F165, F19, G58, K06, P59, SERG) were generated using the Illumina HiSeq platform (Wellcome Trust Centre for Human Genetics, Oxford, UK). An average read count per strain was 12,980,951 (with 43× average coverage), ranging from 11,505,714 reads for the 10 VL isolate (~38× coverage) to 15,457,054 reads for F19 (~51× coverage). Prior to further analysis, reads were subjected to trimming and quality filtering using CLC Genomics Workbench v. 7.0 with the following settings: regions with Phred quality <20 were trimmed, no more than one N was allowed in the remaining sequence, then TruSeq adapter trimming and a minimum length threshold of 75 nt were applied. Filtered reads were mapped to the reference genome of *L. pyrrhocoris* isolate H10 using CLC Genomics Workbench v. 7.0. The mapping parameters were as follows: mismatch cost, 2; insertion cost, 2; deletion cost, 2; length fraction, 0.75; and similarity fraction, 0.95. Variant calling procedure implemented in Platypus^80^ produced 907,015 single nucleotide variants (SNVs) and 261,475 insertions or deletions (indels), 1,168,490 variants in total. Consensus coding sequences (9,878 within the *L. pyrrhocoris* genome) were extracted for each strain, taking into account the observed SNVs, and aligned using MUSCLE with default parameters.

The recombination analysis was performed using Ldhat v.2.2^47^ and suggested the absence of recombination between populations. Selection analysis was performed using the CodeML script from the PAML package v.4.8^54^. We compared the M7 (β) and M8 (β + ω) models for inferring positive selection. Both models are codon-based and allow ω, i.e. the dN/dS ratio (the rate of nonsynonymous substitutions divided by the rate of synonymous substitutions per site), to vary between sites. Model M7 splits codons of analyzed sequence into 9 classes with 0 <ω < 1, while M8 allows one of the codon classes to have ω > 1 (estimated from the data). Comparison between the two models was performed using the likelihood ratio test (LRT). LRT allows determining which of the opposing models, M7 or M8, fits the data better. If the M8 model, which allows ω values >1, fitted the data better than model M7, then positive selection was inferred. It was considered that model M8 fitted the data significantly better than M7 if 2 × (lnL_Model8_ − lnL_Model7_) > 4.61 (10% χ^2^ critical value with 2 degrees of freedom). If the conditions above were satisfied, a gene was considered to be under positive selection. For genes showing a signature of positive selection, an attempt to determine particular codons clustering into the codon class with ω > 1 was made using the empirical Bayes method^81^. A codon was considered to be under positive selection if a *p*-value under model M8 was >0.95.

## Additional Information

**How to cite this article**: Flegontov, P. *et al*. Genome of *Leptomonas pyrrhocoris*: a high-quality reference for monoxenous trypanosomatids and new insights into evolution of *Leishmania*. *Sci. Rep*. **6**, 23704; doi: 10.1038/srep23704 (2016).

## Supplementary Material

Supplementary Information

Supplementary Table S1

## Figures and Tables

**Table 1:** Expression patterns of putative virulence factors gained at the Leishmaniinae node.

OG	Gene IDs	Annotation	Upregulated in		
			*L. seymouri*	*L. mexicana*	*L. major*Friedlin
06971	LmjF.19.0840, LmjF.19.0844, LmjF.19.0848, LbrM2903_19_0920, LbrM.19.1140, LdBPK_190840.1, LinJ.19.0840, LmxM.19.0870, LtaP19.0810	ATG8/AUT7/APG8/PAZ2 (ATG8A.1)	35 °C	META	VIRULENT
09307	LdBPK_051210.1, LinJ.05.1210, LmjF.05.1215, LmxM.05.1215, LbrM2903_05_1260, LbrM.05.1210, LtaP05.1300	surface antigen-like protein	35 °C	PRO and META	VIRULENT
09635	LbrM2903_27_2010, LbrM.27.1900, LdBPK_271680.1, LinJ.27.1680, LmjF.27.1780, LmxM.27.1780, LtaP27.1830	casein kinase I-like protein	no significant changes	META and AMA	VIRULENT
10354	LdBPK_312140.1, LinJ.31.2140, LmjF.31.2090, LmxM.30.2090, LtaP31.2520	protein with unknown function	35 °C	AMA	no significant changes

Only *Leishmania* spp. gene IDs are shown. *Leishmania* life cycle stages are abbreviated as follows: PRO, procyclics; META, metacyclics; AMA, amastigotes.
