# Supplementary material for: Genome of Leptomonas pyrrhocoris: a high-quality reference for monoxenous trypanosomatids and new insights into evolution of Leishmania
Source: Sci Rep. 2016 Mar 29;6:23704. doi: 10.1038/srep23704 (PMC4810370; doi:10.1038/srep23704)
Supplement: Supplementary Information [file srep23704-s1.pdf]

Supplementary Information

Genome of *Leptomonas pyrrhocoris*: a high-quality reference for monoxenous trypanosomatids and new insights into evolution of *Leishmania*

Pavel Flegontov, Anzhelika Butenko, Sergei Firsov, Natalya Kraeva, Marek Eliáš, Mark C. Field, Dmitry Filatov, Olga Flegontova, Evgeny S. Gerasimov, Jana Hlaváčová, Aygul Ishemgulova, Andrew P. Jackson, Steve Kelly, Alexei Yu. Kostygov, Maria D. Logacheva, Dmitri A. Maslov, Fred R. Opperdoes, Amanda O'Reilly, Jovana Sádlová, Tereza Ševčíková, Divya Venkatesh, Čestmír Vlček, Petr Volf, Jan Votýpka, Kristína Záhonová, Vyacheslav Yurchenko, and Julius Lukeš

Contents

Supplementary figures 2

Supplementary notes 33

1. Synteny analysis 33
2. Metabolic pathways 34
3. Trafficking and signaling components 37
4. Cell surface proteins 39
5. Kinase candidates 40
6. Patterns of gene gains and losses 41

Supplementary Methods 44

Supplementary References 47

Supplementary Figures

Suppl. Fig. S1: Schematic representation of the two-way synteny between *L. pyrrhocoris* and *C. fasciculata*. *L. pyrrhocoris* scaffolds are depicted in black; *C. fasciculata* scaffolds are colored according to the legend. Only the chromosomes which actually have synteny blocks are shown.

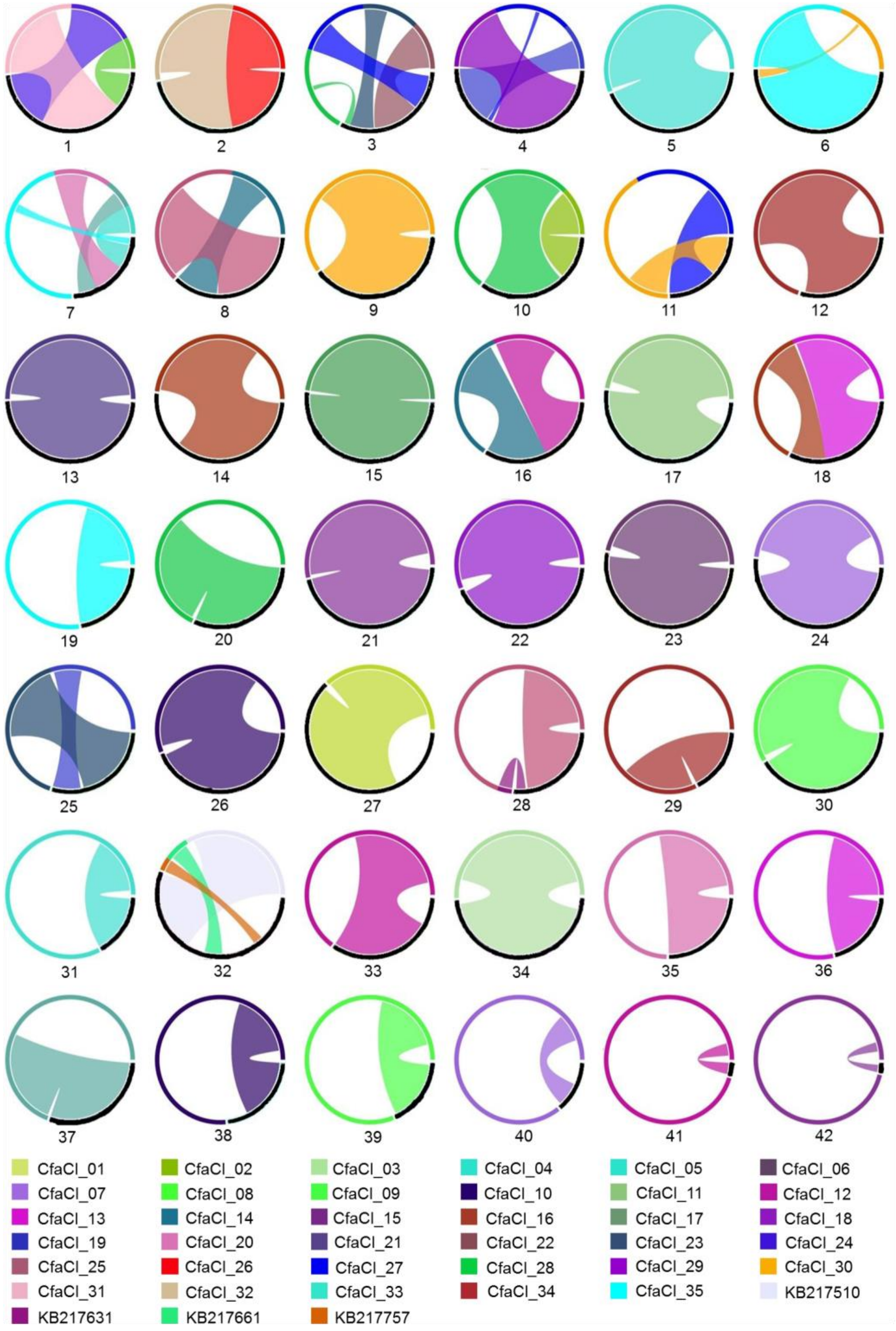

Suppl. Fig. S2: Schematic representation of the two-way synteny between *L. pyrrhocoris* and *L. major* Friedlin. *L. pyrrhocoris* scaffolds are depicted in black; *L. major* Friedlin chromosomes are colored according to the legend. Only the chromosomes which actually have synteny blocks are shown.

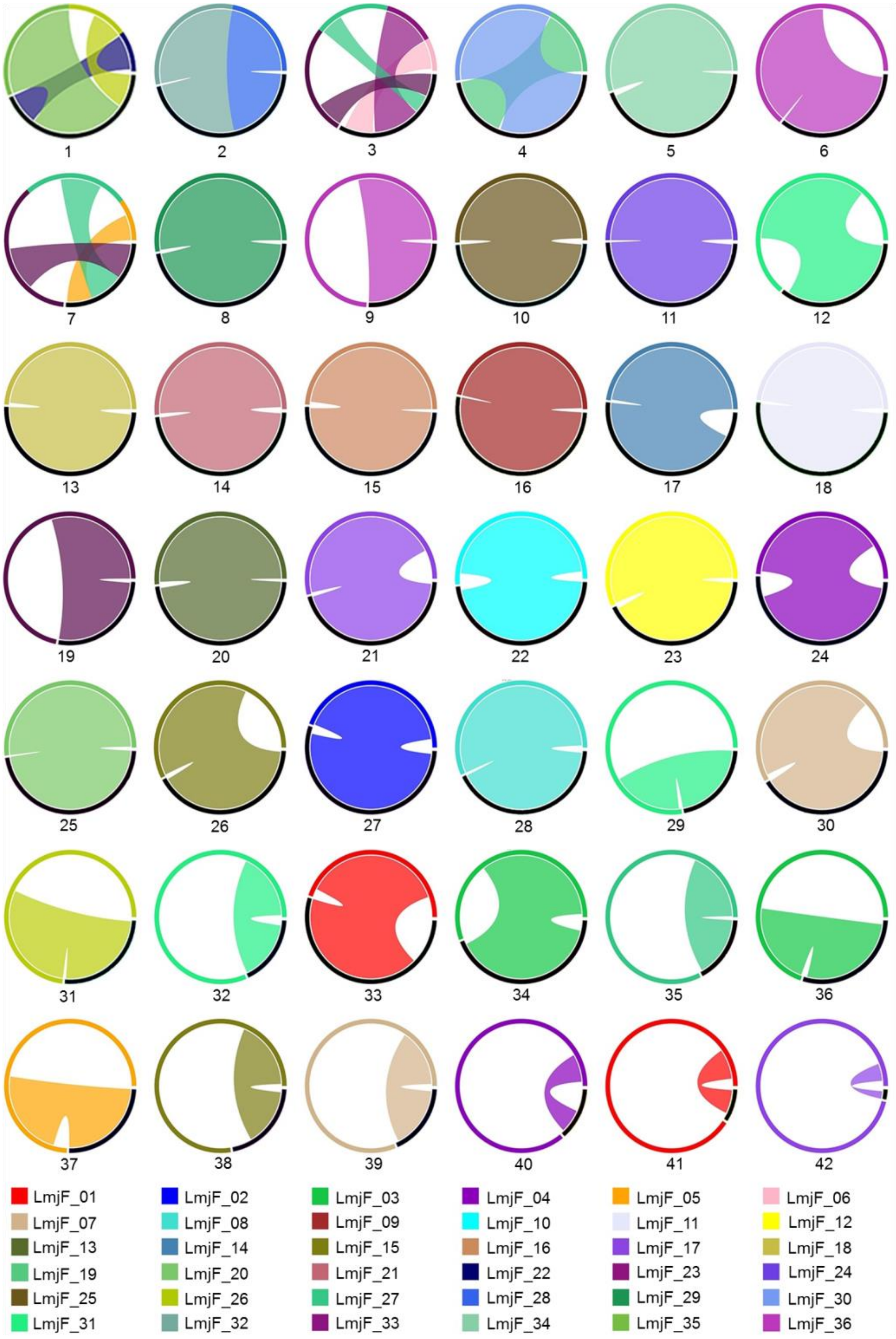

Suppl. Fig. S3: Schematic representation of the two-way synteny between *L. pyrrhocoris* and *T. brucei* TREU927. *L. pyrrhocoris* scaffolds are depicted in black; *T. brucei* chromosomes are colored according to the legend. Only the chromosomes which actually have synteny blocks are shown.

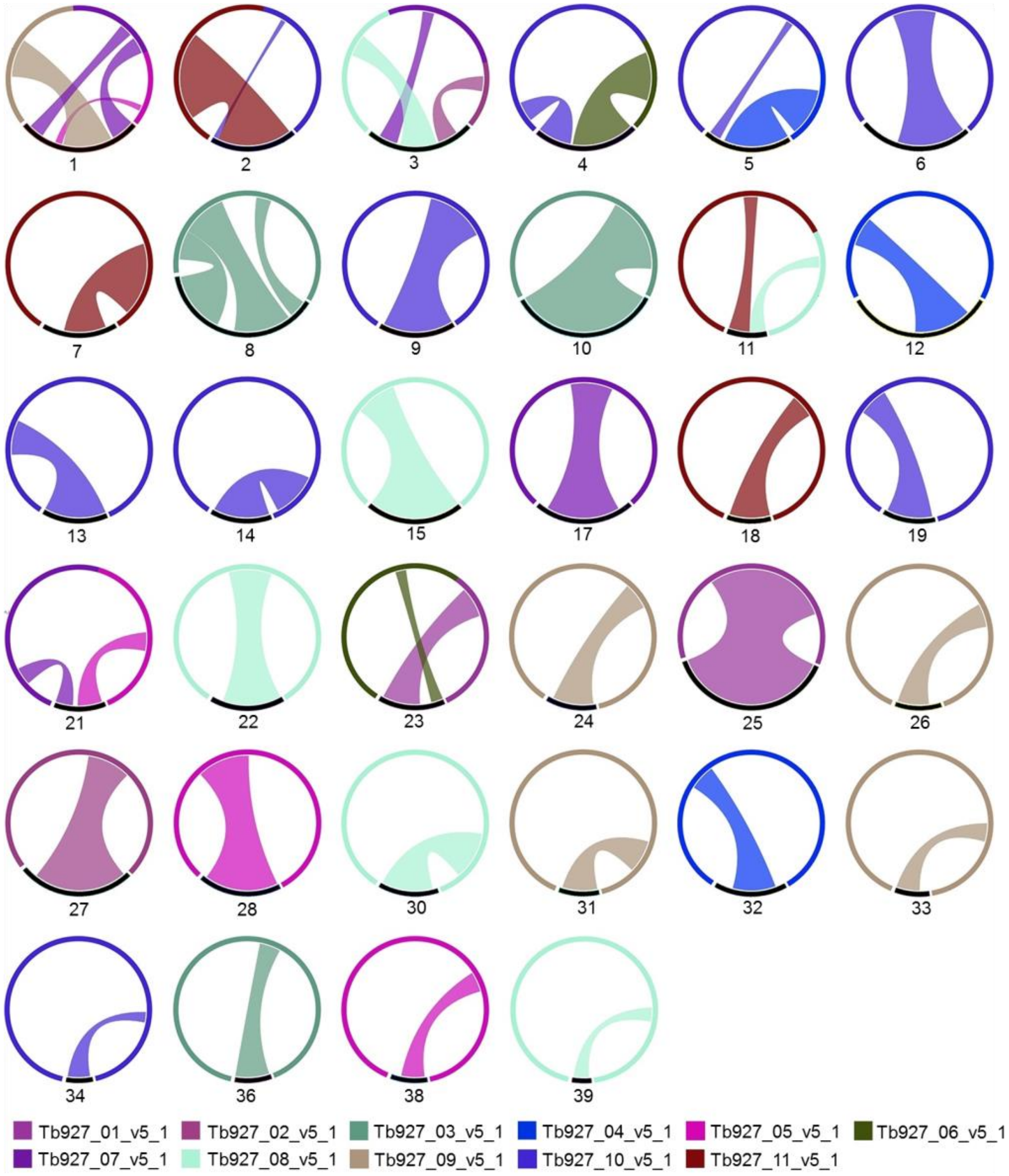

Suppl. Fig. S4: Distribution of groups of paralogs in *L. pyrrhocoris*, *L. seymouri*, *C. fasciculata*, *L. major*, *T. brucei* and *Phytomonas* spp. according to average percent identity at the amino acid level within each group.

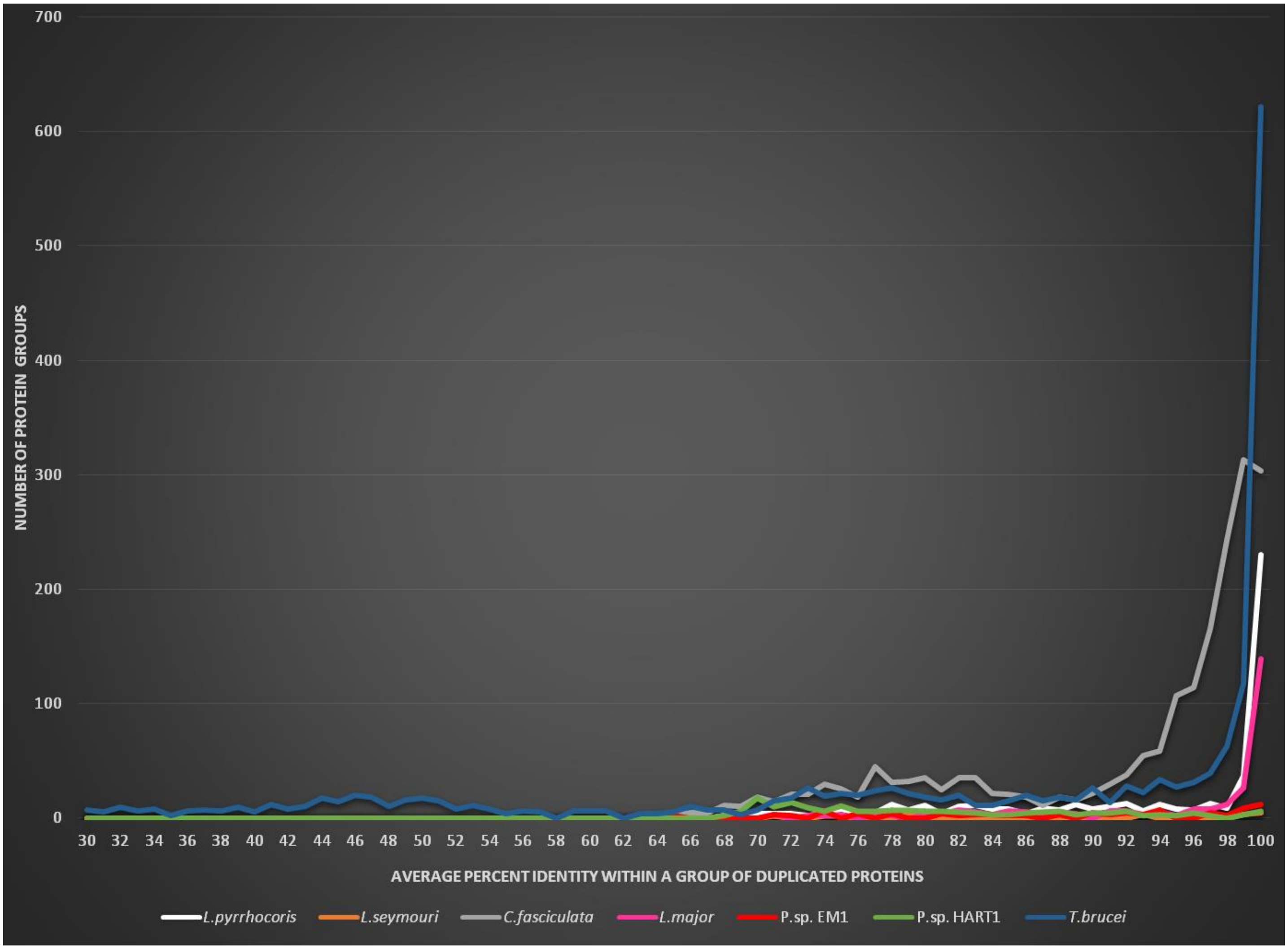

Suppl. Fig. S5: Gene family gains/losses mapped on the tree of kinetoplastids with Wagner parsimony algorithm implemented in the COUNT software.

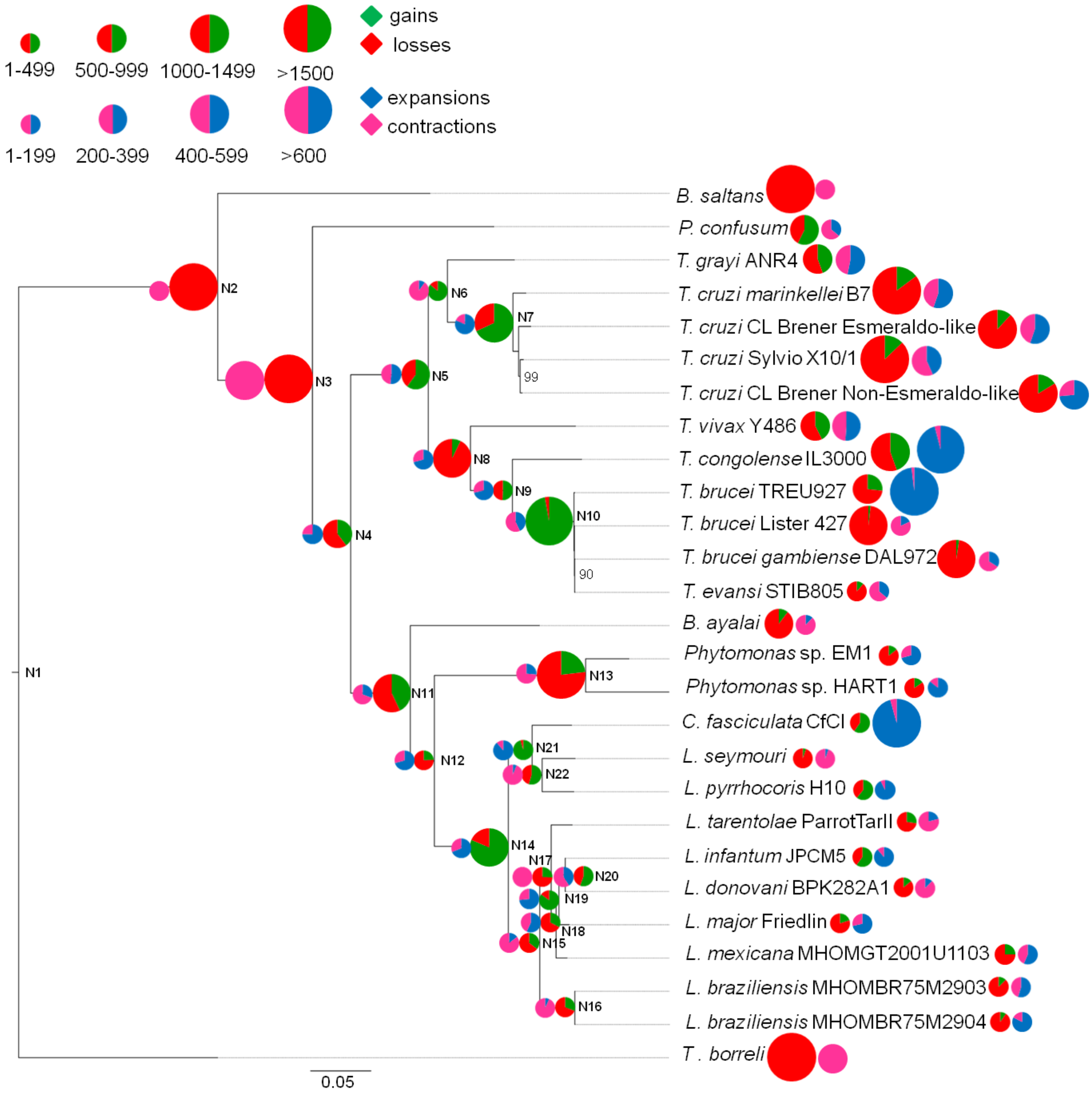

Suppl. Fig. S6: Comparative analysis of SNARE proteins in *L. pyrrhocoris*. Accession numbers and gene copy number are given for *T.brucei* as a reference, followed by the equivalent assignments for T. *cruzi*, *Leishmania* and *Leptomonas*. Filled dots indicate the presence of an ortholog and numbers within the dots the number of paralogs. At left are the predicted domain structures of these proteins. The nomenclature and figure are adapted from[1].

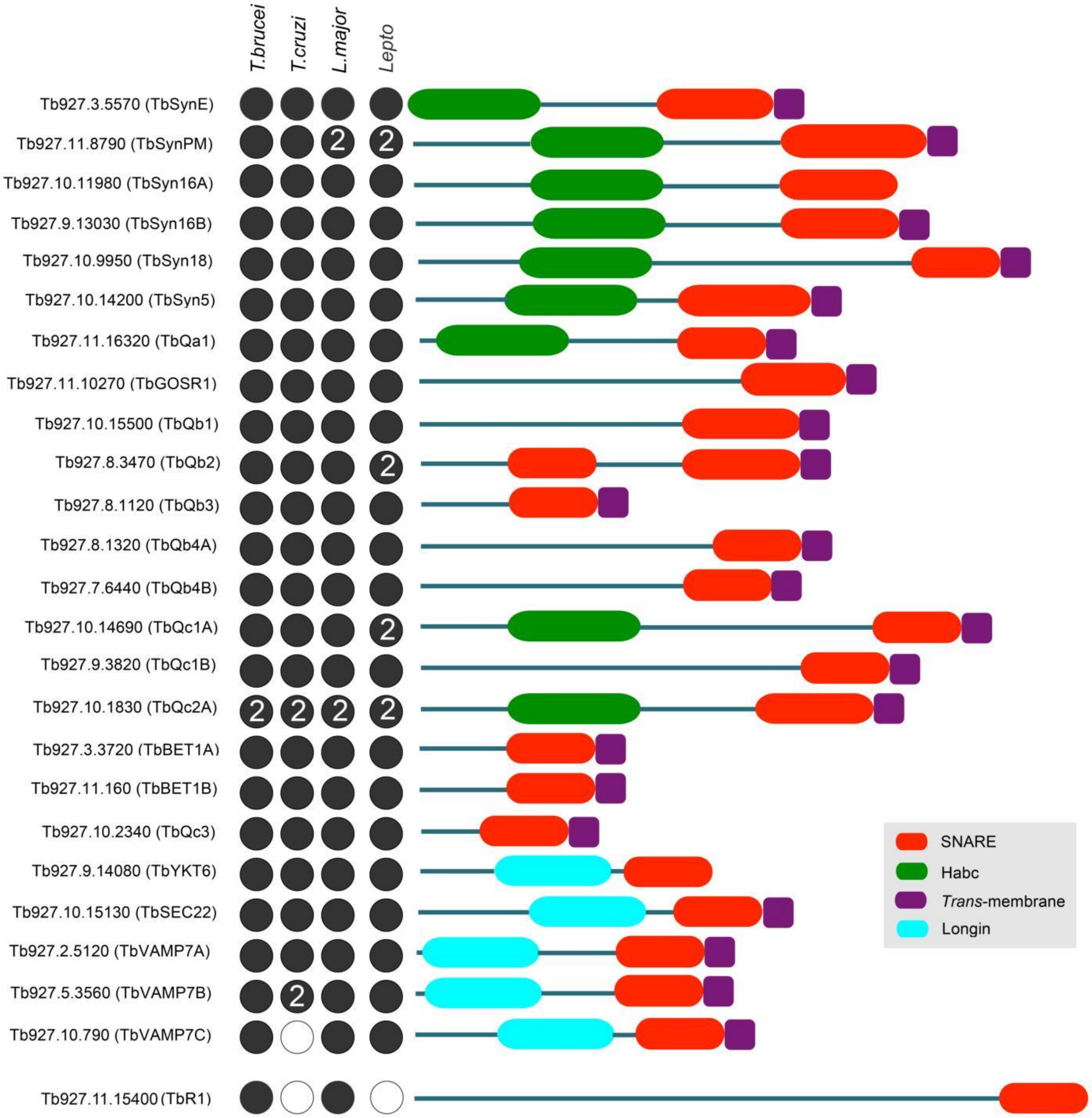

Suppl. Fig. S7: Reconstruction of the GPI-anchor precursor biosynthesis pathway. Presence of orthologs was determined by reverse BLAST and other criteria as indicated in Methods, and detected orthologs are indicated by filled circles. Gray circles indicate reverse BLAST hits that fall into a lower significance class due to one or more criteria for orthology failing and therefore are classed as tentative.

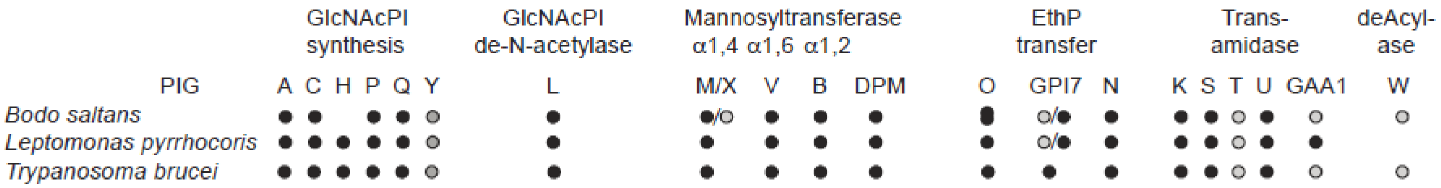

Suppl. Fig. S8: Maximum likelihood phylogeny of amastin-like amino acid sequences from *L. pyrrhocoris* and related trypanosomatids. The tree was estimated using PhyML and the LG + Γ model. Branches subtending nodes with bootstrap support > 75 are shown in bold. Terminal nodes are labelled with TriTrypDB gene identifiers according to species: LmjF (*L. major* Friedlin), Linj (*L. infantum*), LbrM (*L. braziliensis*), LmxM (*L. mexicana*), CfaC1 (*C. fasciculata* CfCl), TcCLB (*T. cruzi* CL Brener), and Tb (*T. brucei*). *L. pyrrhocoris* genes are shaded pink. The tree is rooted using the divergent α-amastin sub-family.

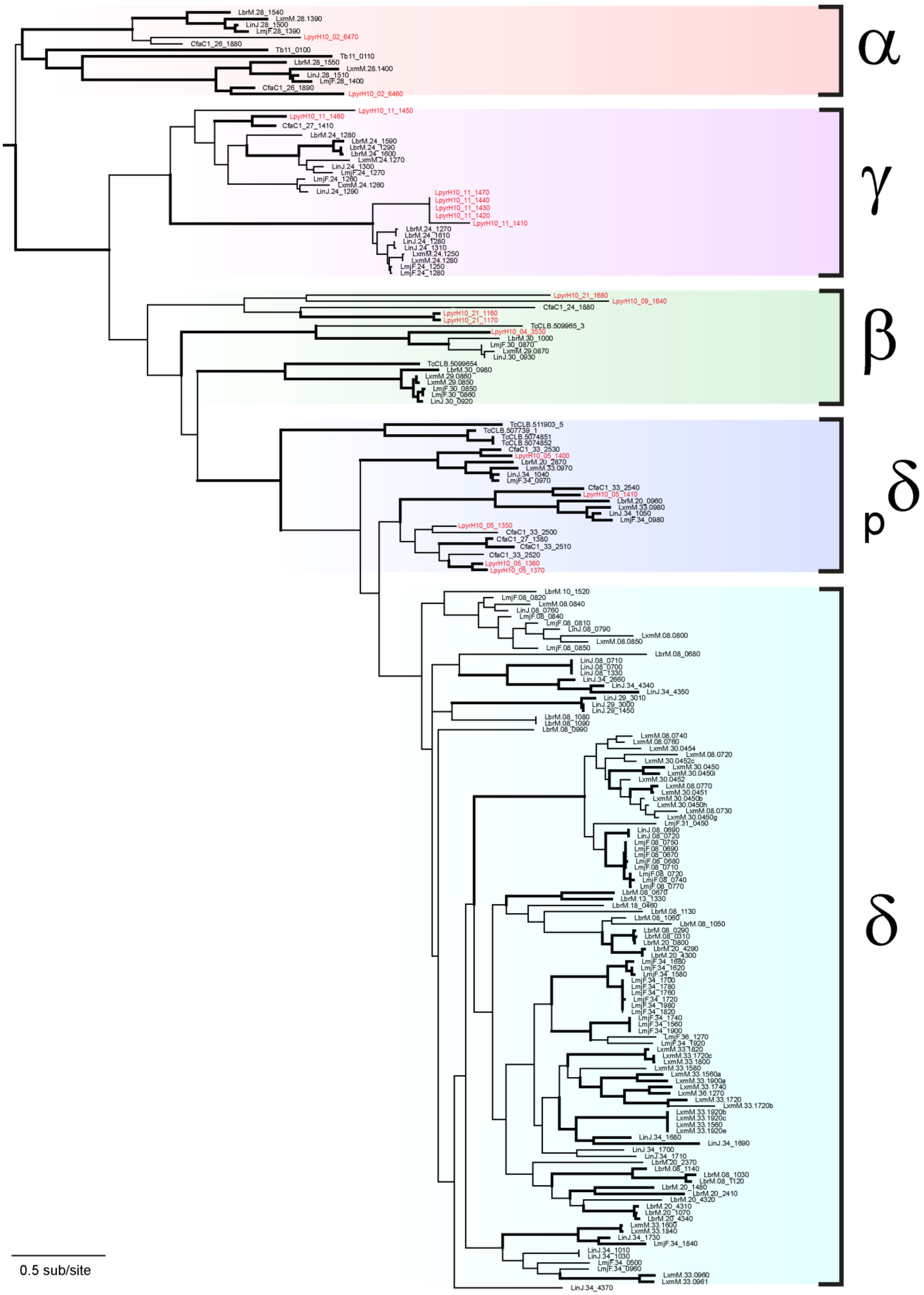
α
γ
β
pδ
δ
0.5 sub/site

Supp. Fig. S9: Maximum likelihood phylogeny of Promastigote Surface Antigen-like (PSA-like) amino acid sequences from *L. pyrrhocoris* and related trypanosomatids. The tree was estimated using PhyML and the LG + Γ model. Branches subtending nodes with bootstrap support > 75 and Bayesian posterior probabilities > 0.9 are shown in bold. Node robustness at two basal nodes is specified with three measures: aLRT SH-test/ML bootstrap proportions/Bayesian posterior probabilities. Terminal nodes are labelled with TriTrypDB gene identifiers according to species: LmjF (*L. major* Friedlin), Linj (*L. infantum*), LbrM (*L. braziliensis*), LmxM (*L. mexicana*), CfaC1 (*C. fasciculata* CfCl), TcCLB (*T. cruzi* CL Brener), and Tb (*T. brucei*). *L. pyrrhocoris* genes are shaded pink. The tree is rooted using an outgroup consisting of a single *Trypanosoma cruzi* homolog (TcCLB.508387.80).

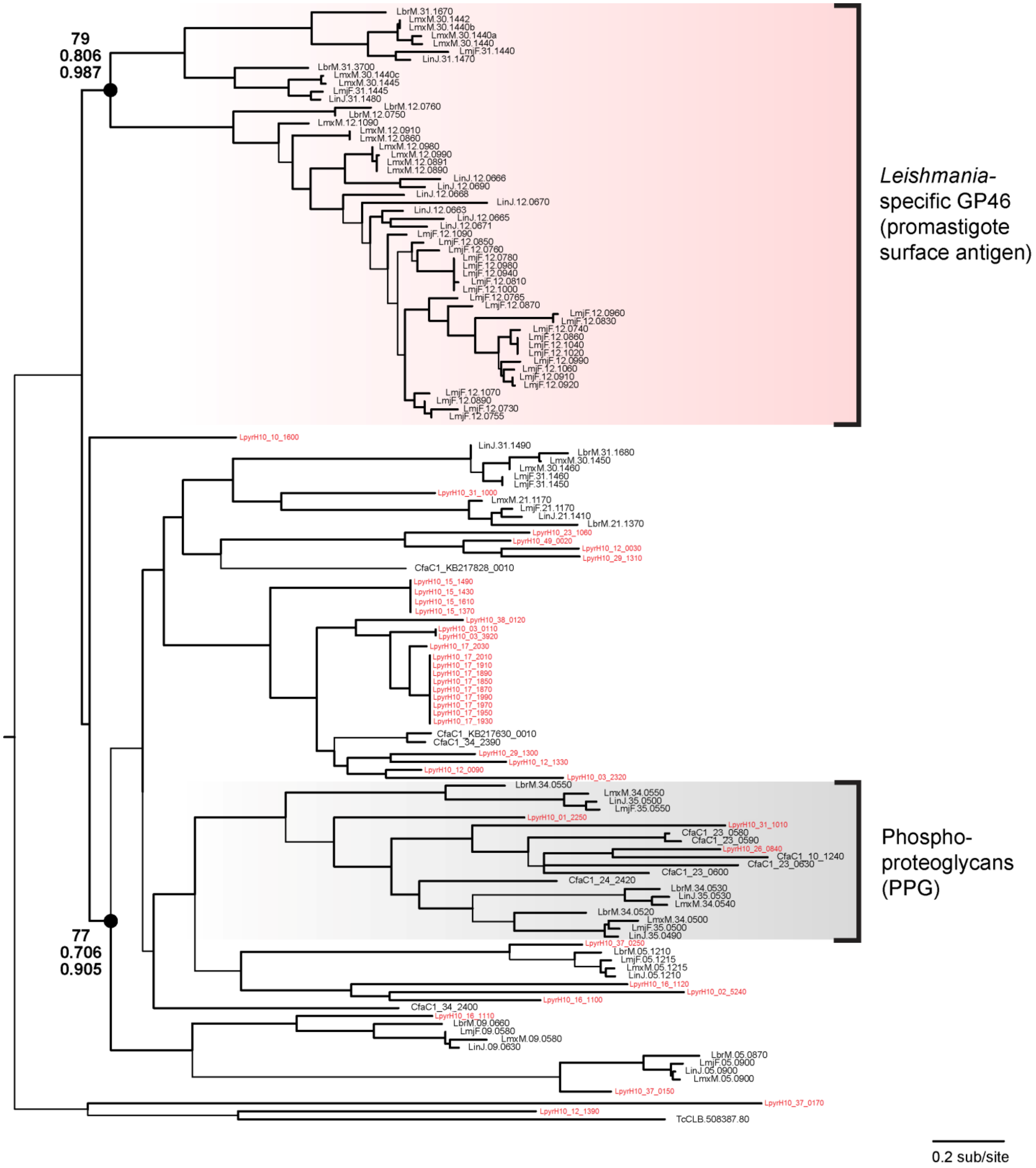

79
0.806
0.987
LbrM.31.1670
LmxM.30.1442
LmxM.30.1440b
LmxM.30.1440a
LmxM.30.1440
LmjF.31.1440
LinJ.31.1470
LbrM.31.3700
LmxM.30.1440c
LmxM.30.1445
LmjF.31.1445
LinJ.31.1480
LbrM.12.0760
LbrM.12.0750
LmxM.12.1090
LmxM.12.0910
LmxM.12.0860
LmxM.12.0980
LmxM.12.0990
LmxM.12.0891
LmxM.12.0890
LinJ.12.0666
LinJ.12.0690
LinJ.12.0668
LinJ.12.0670
LinJ.12.0663
LinJ.12.0665
LinJ.12.0671
LmjF.12.1090
LmjF.12.0850
LmjF.12.0760
LmjF.12.0780
LmjF.12.0980
LmjF.12.0940
LmjF.12.0810
LmjF.12.1000
LmjF.12.0765
LmjF.12.0870
LmjF.12.0960
LmjF.12.0830
LmjF.12.0740
LmjF.12.0860
LmjF.12.1040
LmjF.12.1020
LmjF.12.0990
LmjF.12.1060
LmjF.12.0910
LmjF.12.0920
LmjF.12.1070
LmjF.12.0890
LmjF.12.0730
LmjF.12.0755
Leishmania-specific GP46 (promastigote surface antigen)
LpyrH10_10_1600
LinJ.31.1490
LbrM.31.1680
LmxM.30.1450
LmxM.30.1460
LmjF.31.1460
LmjF.31.1450
LpyrH10_31_1000
LmxM.21.1170
LmjF.21.1170
LinJ.21.1410
LbrM.21.1370
LpyrH10_23_1060
LpyrH10_49_0020
LpyrH10_12_0030
LpyrH10_29_1310
CfaC1_KB217828_0010
LpyrH10_15_1490
LpyrH10_15_1430
LpyrH10_15_1610
LpyrH10_15_1370
LpyrH10_38_0120
LpyrH10_03_0110
LpyrH10_03_3920
LpyrH10_17_2030
LpyrH10_17_2010
LpyrH10_17_1910
LpyrH10_17_1890
LpyrH10_17_1850
LpyrH10_17_1870
LpyrH10_17_1990
LpyrH10_17_1970
LpyrH10_17_1950
LpyrH10_17_1930
CfaC1_KB217630_0010
CfaC1_34_2390
LpyrH10_29_1300
LpyrH10_12_1330
LpyrH10_12_0090
LpyrH10_03_2320
LbrM.34.0550
LmxM.34.0550
LinJ.35.0500
LmjF.35.0550
LpyrH10_01_2250
LpyrH10_31_1010
CfaC1_23_0580
CfaC1_23_0590
LpyrH10_26_0840
CfaC1_10_1240
CfaC1_23_0630
CfaC1_23_0600
CfaC1_24_2420
LbrM.34.0530
LinJ.35.0530
LmxM.34.0540
LbrM.34.0520
LmxM.34.0500
LmjF.35.0500
LinJ.35.0490
Phospho-proteoglycans (PPG)
77
0.706
0.905
LpyrH10_37_0250
LbrM.05.1210
LmjF.05.1215
LmxM.05.1215
LinJ.05.1210
LpyrH10_16_1120
LpyrH10_02_5240
LpyrH10_16_1100
CfaC1_34_2400
LpyrH10_16_1110
LbrM.09.0660
LmjF.09.0580
LmxM.09.0580
LinJ.09.0630
LbrM.05.0870
LmjF.05.0900
LinJ.05.0900
LmxM.05.0900
LpyrH10_37_0150
LpyrH10_37_0170
LpyrH10_12_1390
TcCLB.508387.80
0.2 sub/site

Suppl. Fig. S10: Gene family gains/losses/expansions/contractions mapped on the tree of kinetoplastids with Wagner parsimony algorithm implemented in the COUNT software. Gain/loss counts for the nodes are plotted beside node names in the following format: + number of gains/ - number of losses/number of expansions/number of contractions. Gain/loss counts for the leafs are shown on the right. The difference between the number of gain and loss events for each leaf is shown in the third column. If the number of gains exceeds the number of losses the value is colored in green. In the cases when losses prevail the corresponding number is colored in red and has "-" sign. The difference between the number of expansions and contractions for each leaf is shown in the last column. If the number of expansions exceeds the number of contractions the value is colored in blue. In the cases when contractions prevail the corresponding number is colored in black.

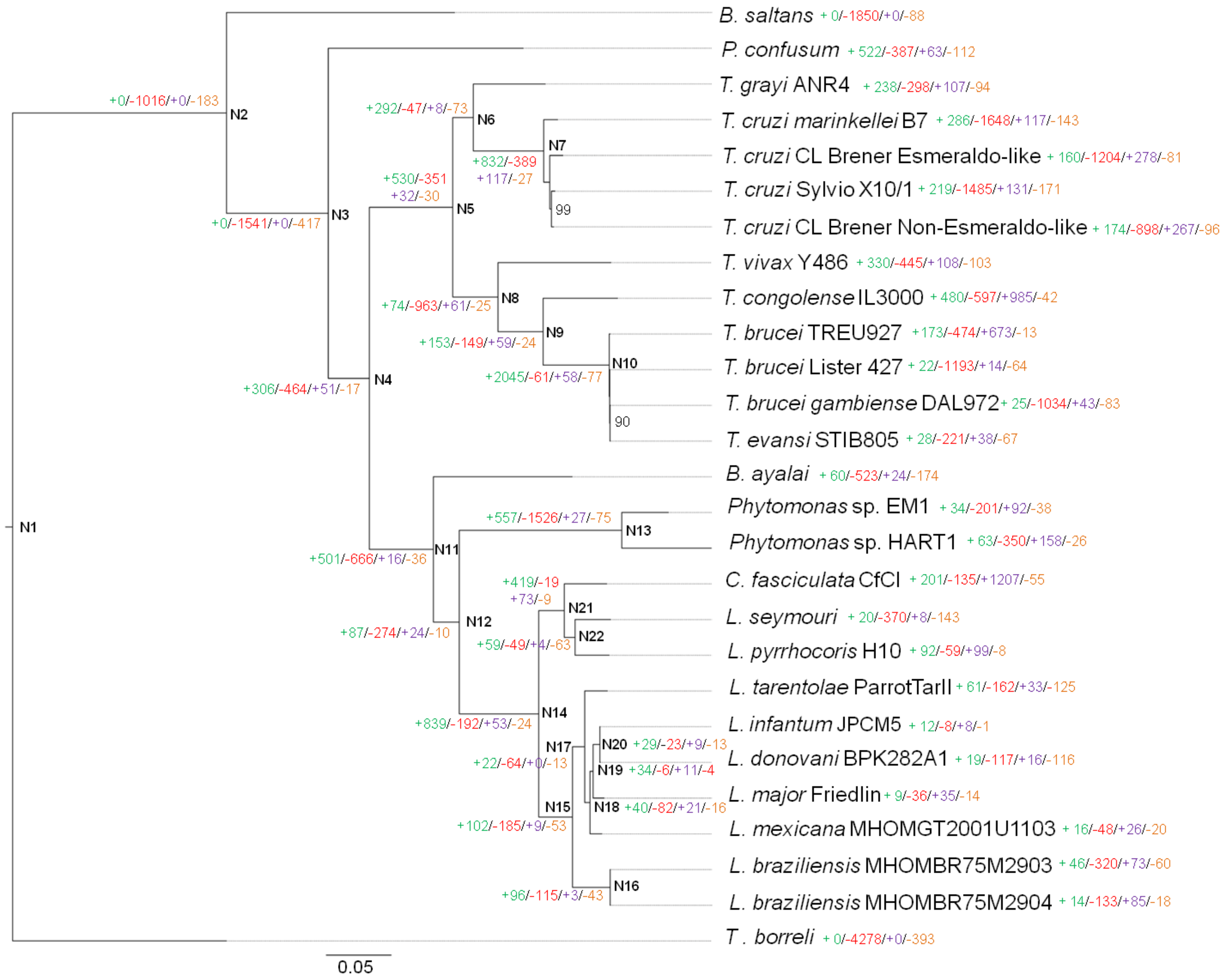

Suppl. Fig. S11: Gene family gains/losses mapped on the tree of kinetoplastids with Dollo parsimony algorithm implemented in the COUNT software. Gain/loss counts for the nodes are plotted beside node names in the following format: + number of gains/ - number of losses. Gain/loss counts for the leafs are shown on the right. The difference between the number of gain and loss events for each leaf is shown in the last column. If the number of gains exceeds the number of losses the number is colored in green. In the cases when losses prevail the corresponding number is colored in red.

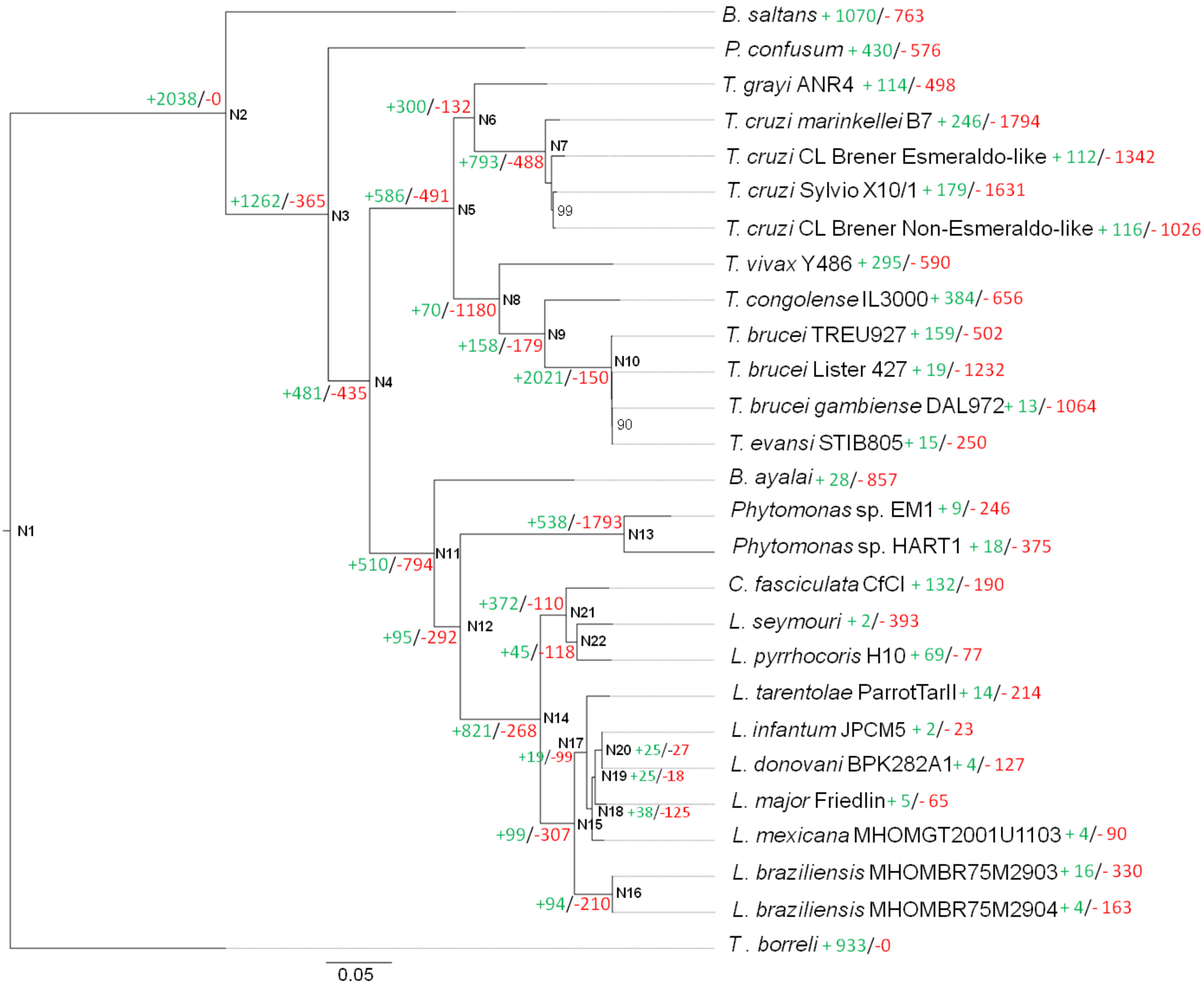

Suppl. Fig. S12: Distribution of GO terms across the ‘biological process’ GO category assigned to genes gained at the *Leishmania* node. Due to a small number of functionally annotated genes gained at this node, 'molecular function' and 'cellular component' GO categories are not informative and are not shown. Numbers in brackets represent gene counts associated with each GO category. A multi-level pie chart was generated using the sequence filter set to 5 (minimal number of sequences a GO node must contain in order to be displayed). GO terms within all of the lowest nodes of the annotation graph that fulfill the filter condition are shown.

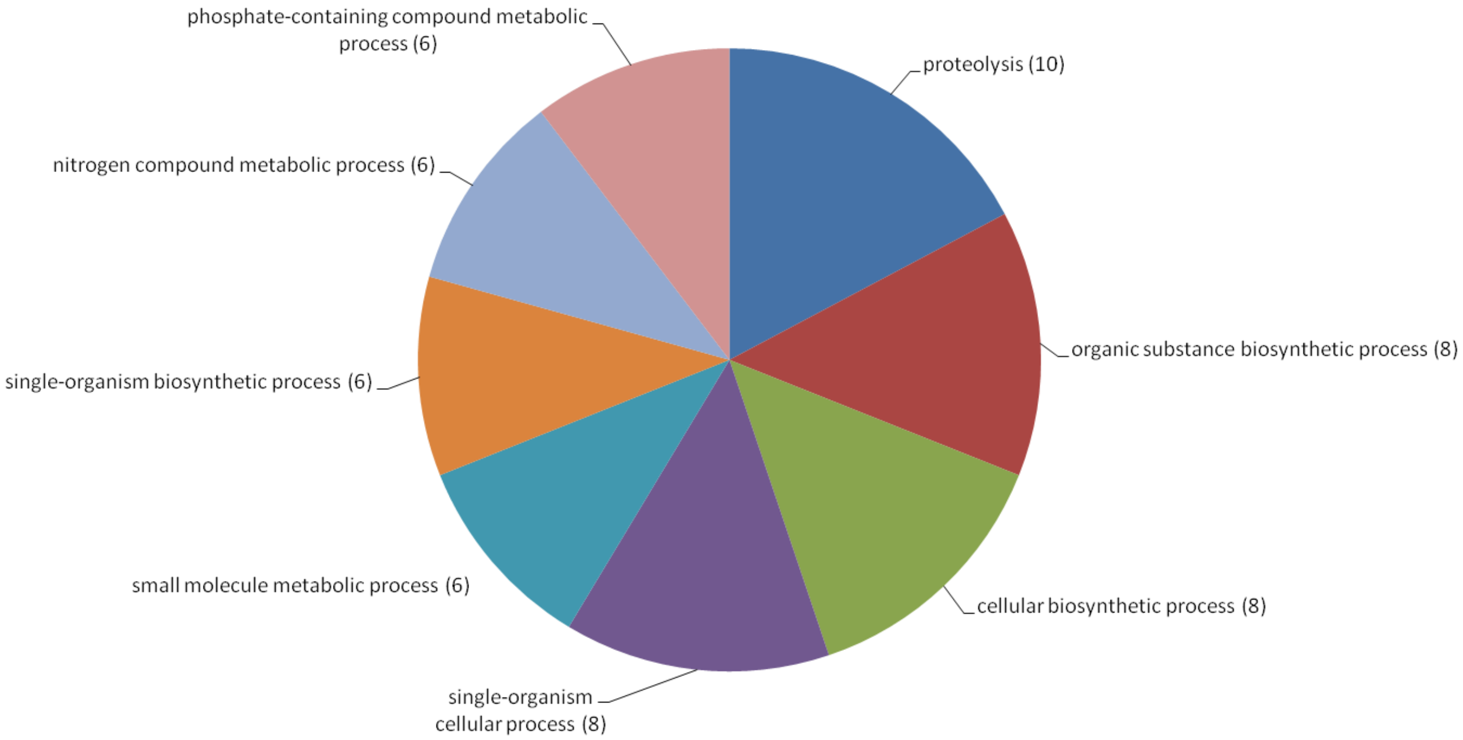

Suppl. Fig. S13: Distribution of GO terms across the 'biological process' GO category assigned to genes within OGs that underwent expansion at the *Leishmania* node. Numbers in brackets represent gene counts associated with each GO category. A multi-level pie chart was generated using the sequence filter set to 10 (minimal number of sequences a GO node must contain in order to be displayed). GO terms within all of the lowest nodes of the annotation graph that fulfill the filter condition are shown.

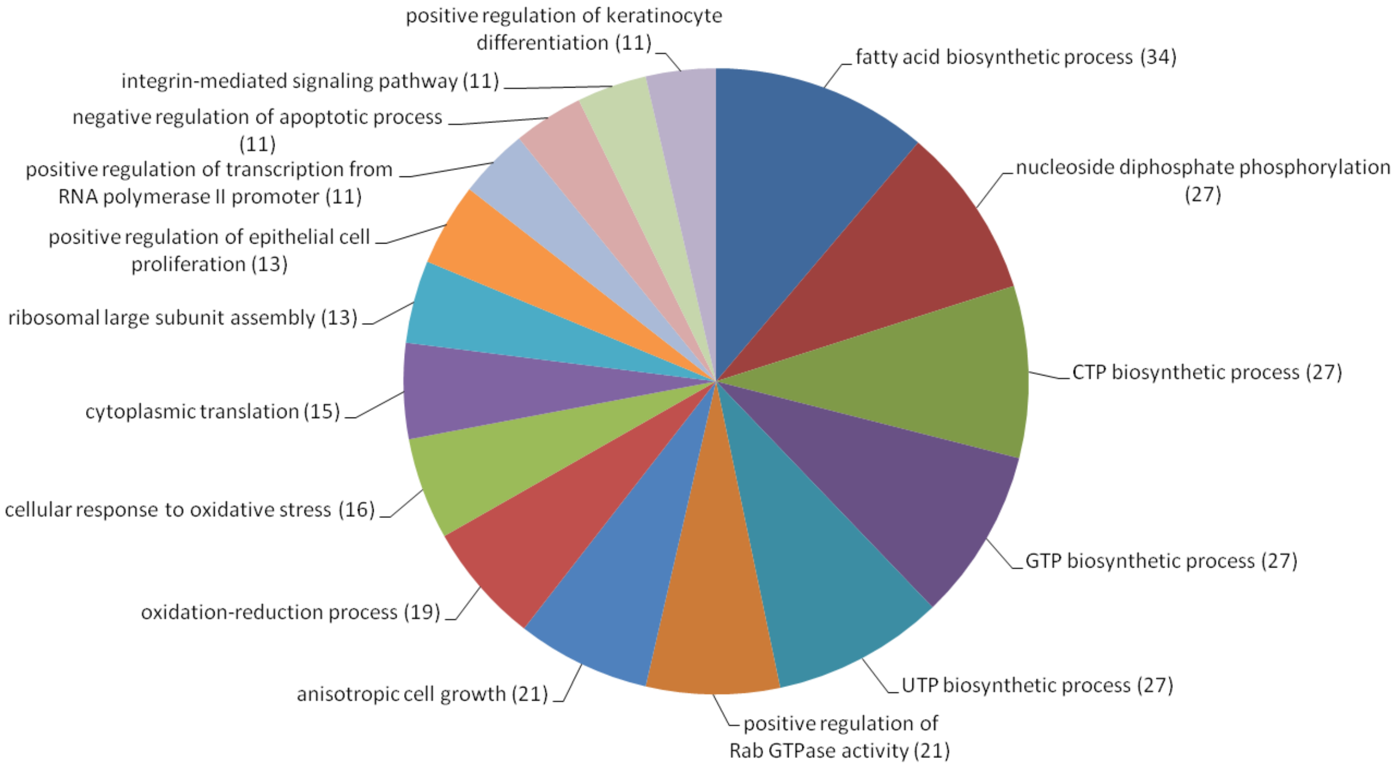

Suppl. Fig. S14: Distribution of GO terms across the 'molecular function' GO category assigned to genes within OGs that underwent expansion at the *Leishmania* node. Numbers in brackets represent gene counts associated with each GO category. A multi-level pie chart was generated using the sequence filter set to 10 (minimal number of sequences a GO node must contain in order to be displayed). GO terms within all of the lowest nodes of the annotation graph that fulfill the filter condition are shown.

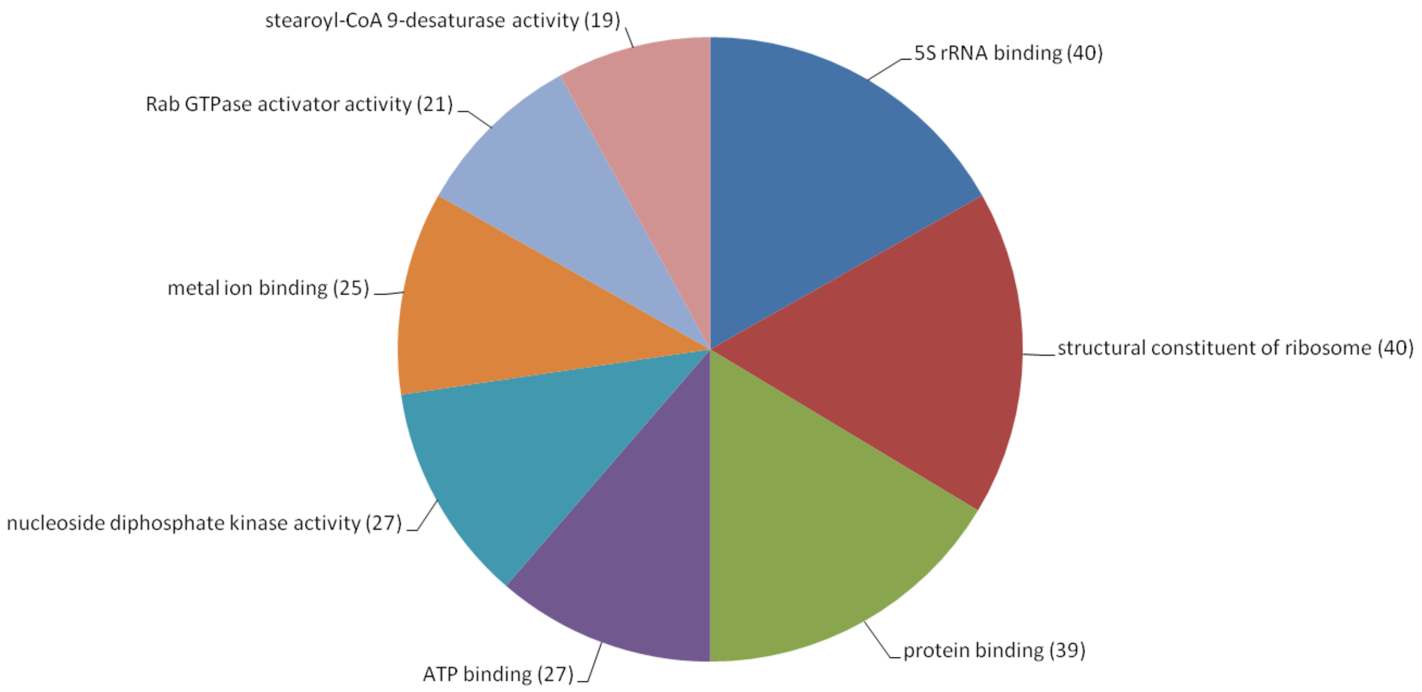

Suppl. Fig. S15: Distribution of GO terms across the ‘cellular component’ GO category assigned to genes within OGs that underwent expansion at the *Leishmania* node. Numbers in brackets represent gene counts associated with each GO category. A multi-level pie chart was generated using the sequence filter set to 10 (minimal number of sequences a GO node must contain in order to be displayed). GO terms within all of the lowest nodes of the annotation graph that fulfill the filter condition are shown.

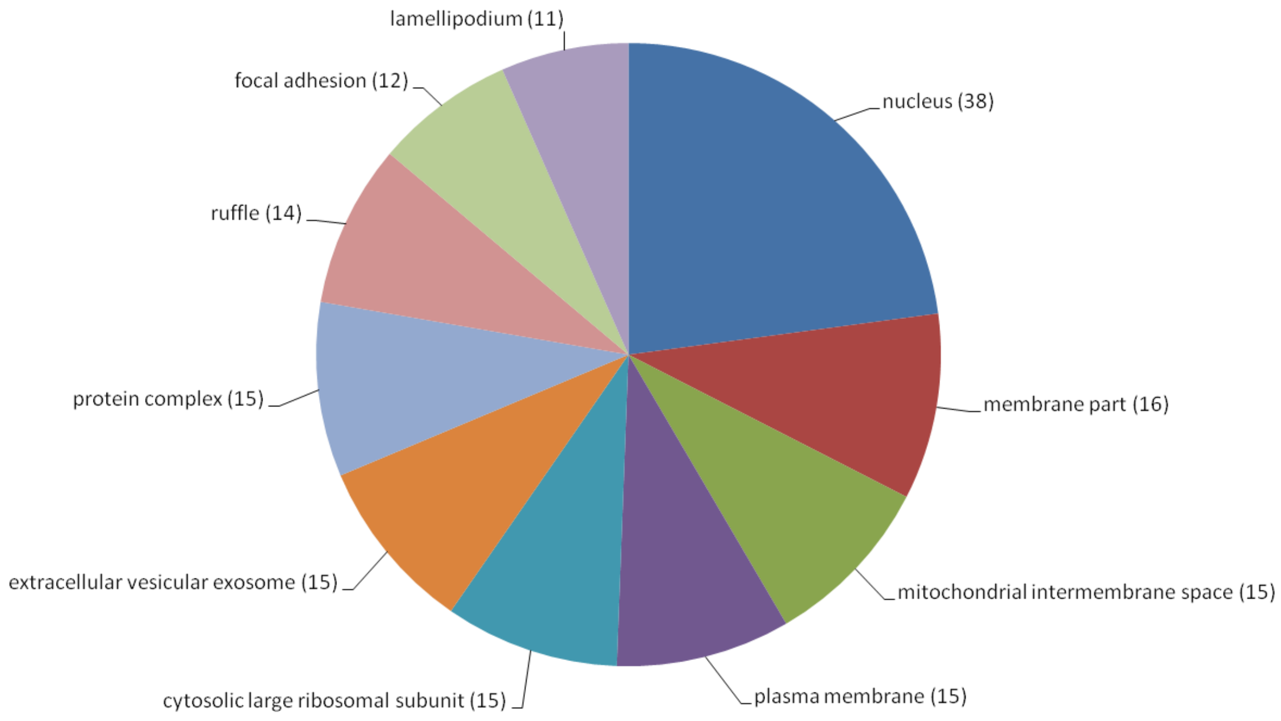

Suppl. Fig. S16: Gain nodes inferred with Dollo parsimony algorithm for orthologous groups lost at the *Leishmania* node.

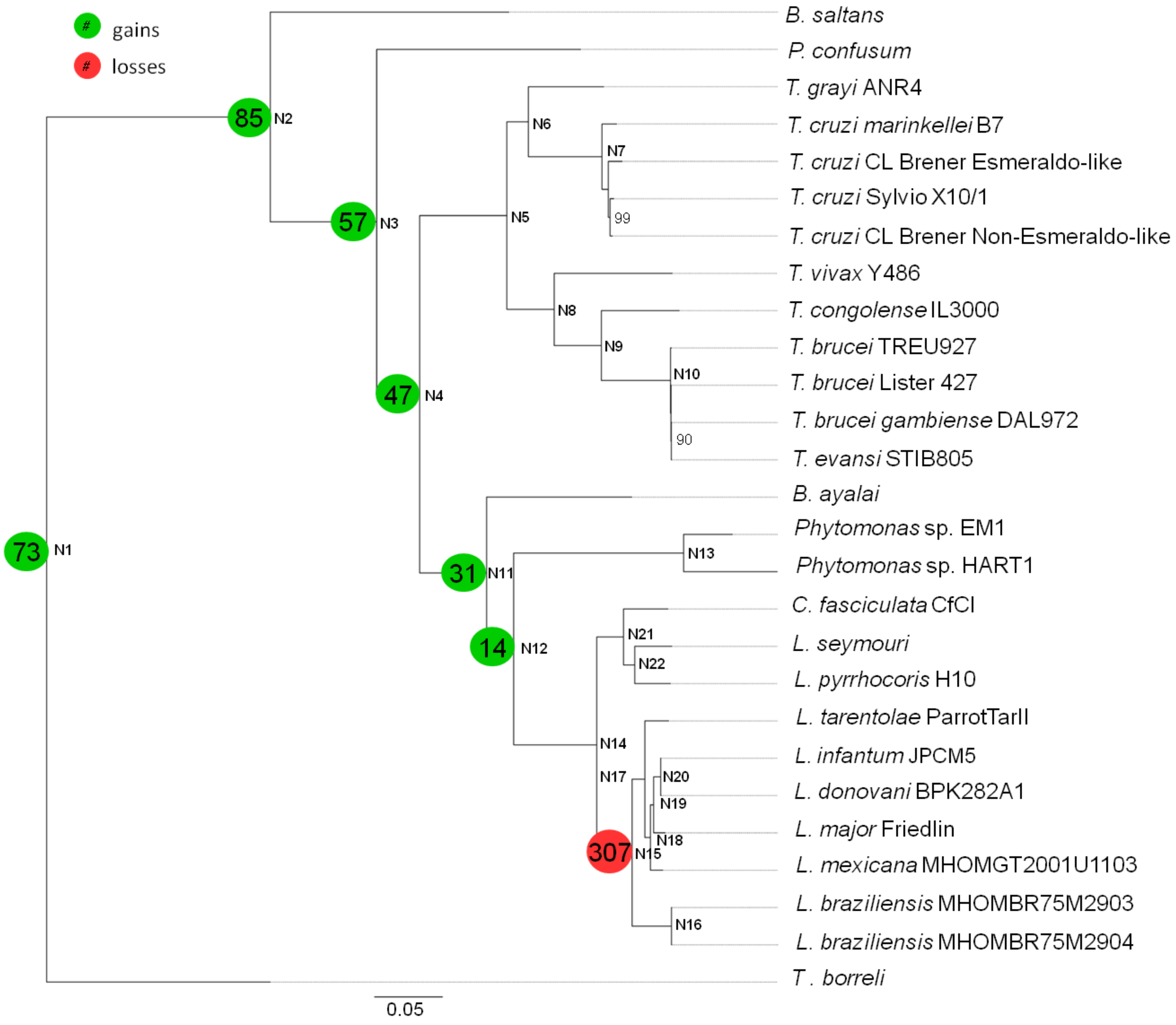

Suppl. Fig. S17: Gene family gains (in green) and losses (in red) mapped on the Leishmaniinae subtree with Dollo parsimony algorithm implemented in the COUNT software.

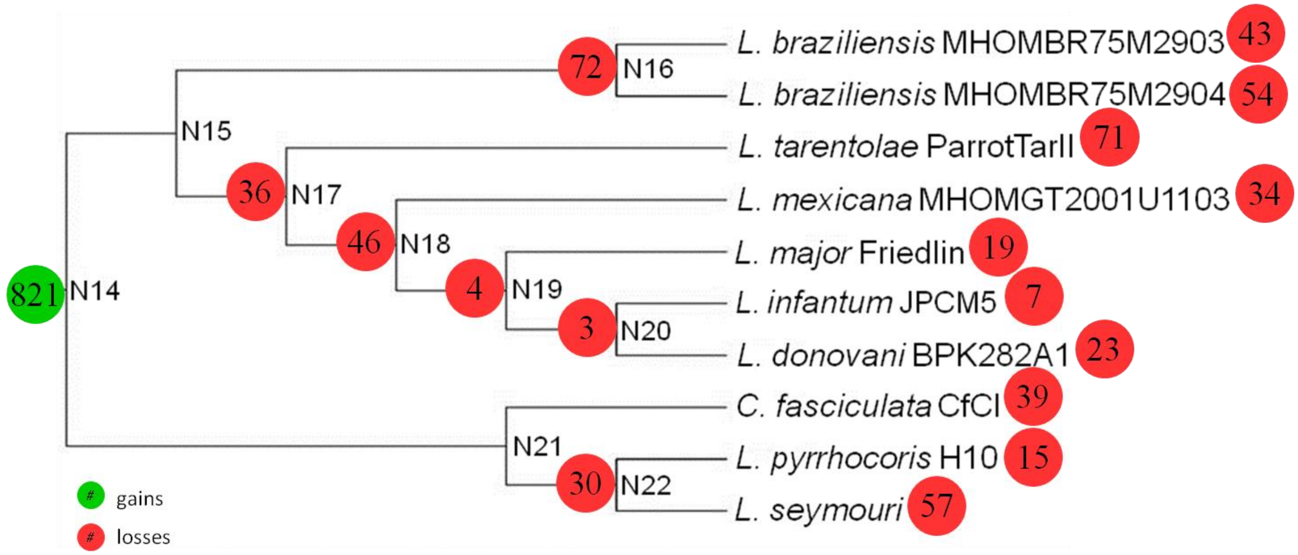

Suppl. Fig. S18: A multi-level pie chart demonstrating distribution of GO terms across the 'biological process' GO category assigned to genes gained at the Leishmaniinae node. Numbers in brackets represent gene counts associated with each GO category. A multi-level pie chart was generated using the sequence filter set to 5 (minimal number of sequences a GO node must contain in order to be displayed). GO terms within all of the lowest nodes of the annotation graph that fulfill the filter condition are shown. Slice 'other' represents GO terms assigned to less than 1% of sequences.

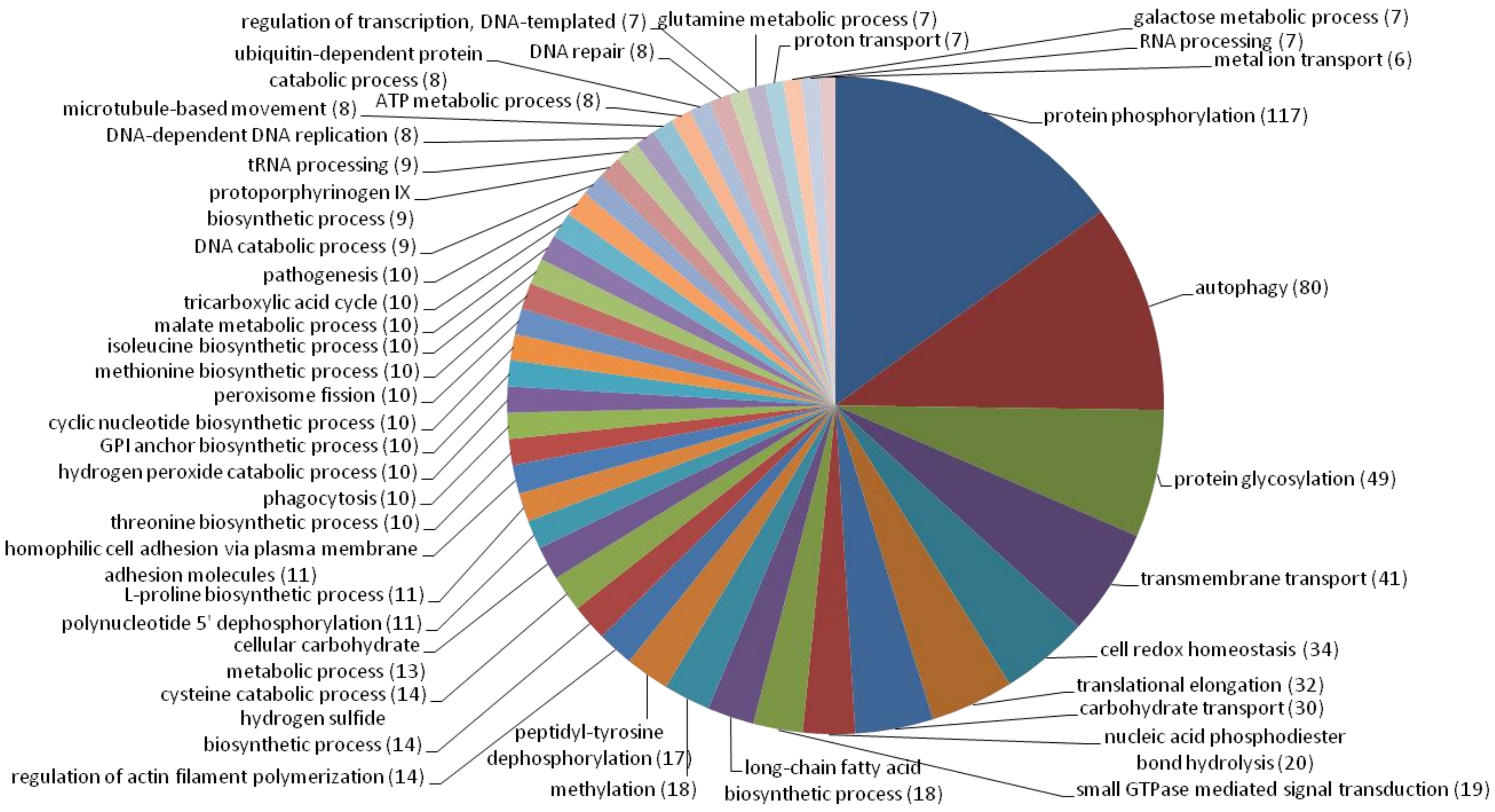

Suppl. Fig. S19: A multi-level pie chart demonstrating distribution of GO terms across the “molecular function” GO category assigned to genes gained at the Leishmaniinae node. Numbers in brackets represent gene counts associated with each GO category. A multi-level pie chart was generated using the sequence filter set to 5 (minimal number of sequences a GO node must contain in order to be displayed). GO terms within all of the lowest nodes of the annotation graph that fulfill the filter condition are shown. Slice "other" was enabled for pie charts and represents GO terms that were assigned to less than 1 % of sequences.

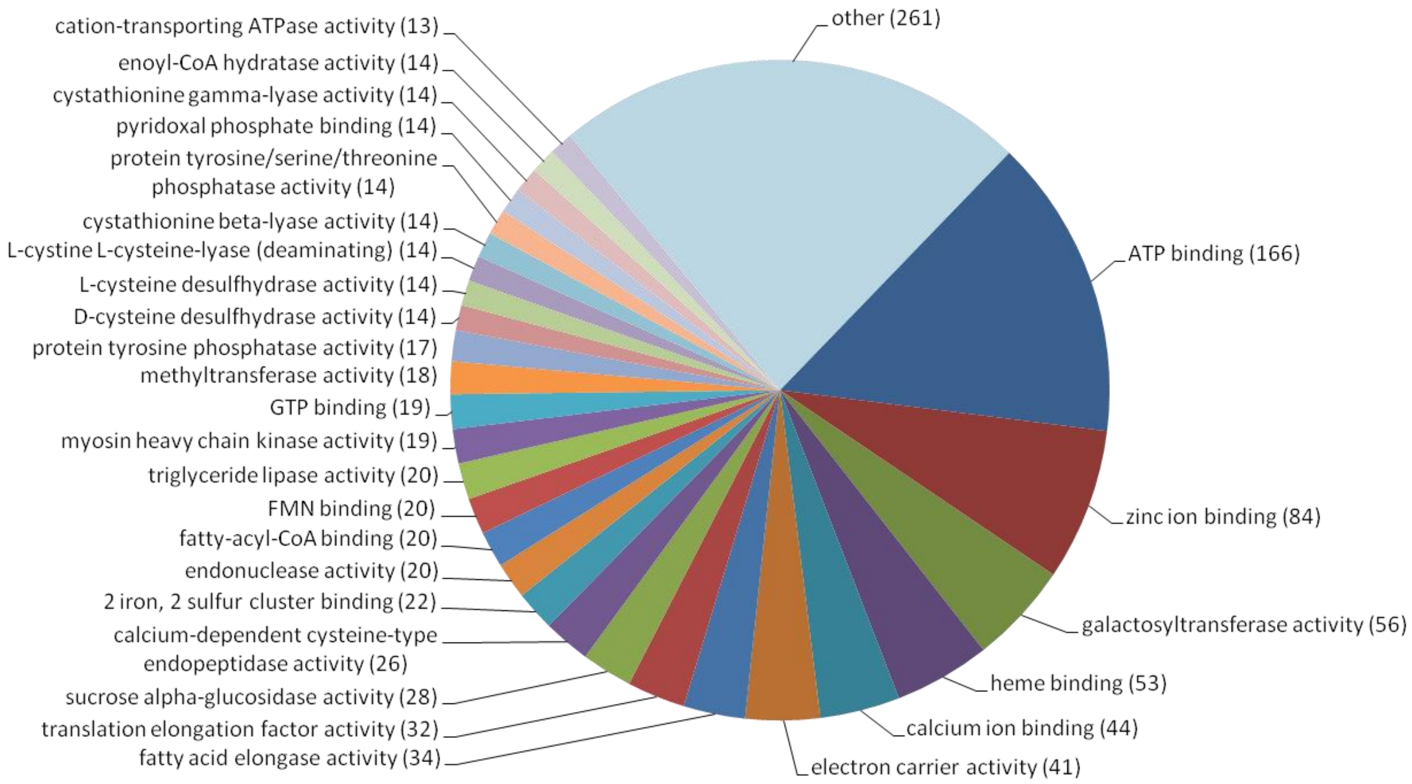

Suppl. Fig. S20: A multi-level pie chart demonstrating distribution of GO terms across the "cellular component" GO category assigned to genes gained at the Leishmaniinae node. Numbers in brackets represent gene counts associated with each GO category. A multi-level pie chart was generated using the sequence filter set to 5 (minimal number of sequences a GO node must contain in order to be displayed). GO terms within all of the lowest nodes of the annotation graph that fulfill the filter condition are shown.

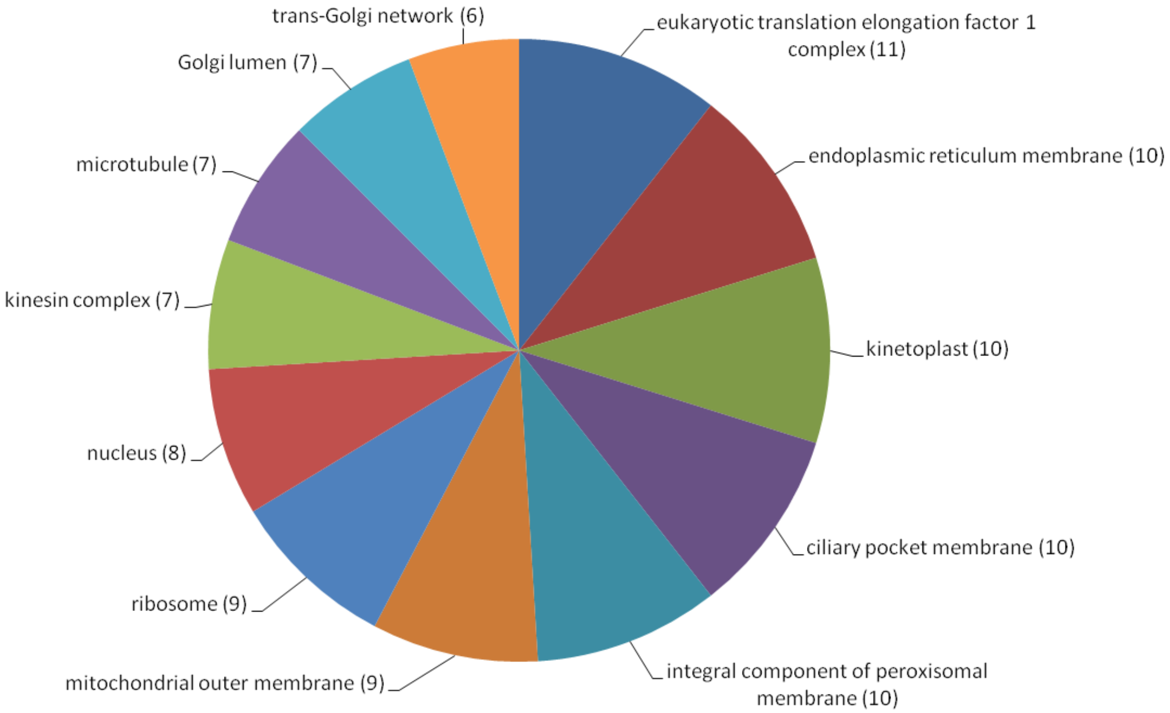

Suppl. Fig. S21: A multi-level pie chart demonstrating distribution of GO terms across the 'biological process' GO category assigned to genes gained at the node of *L. pyrrhocoris*, *C. fasciculata* and *L. seymouri*. Numbers in brackets represent gene counts associated with each GO category. A multi-level pie chart was generated using the sequence filter set to 5 (minimal number of sequences a GO node must contain in order to be displayed). GO terms within all of the lowest nodes of the annotation graph that fulfill the filter condition are shown.

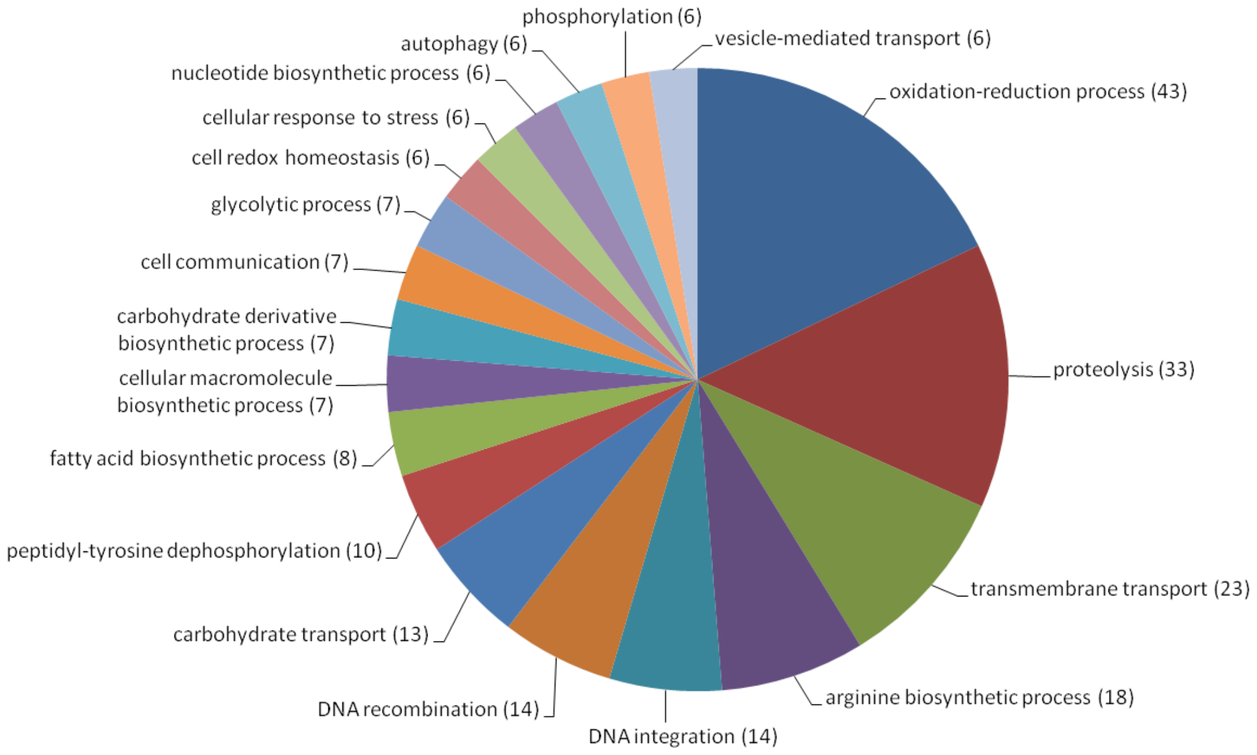

Suppl. Fig. S22: A multi-level pie chart demonstrating distribution of GO terms across the 'molecular function' GO category assigned to genes gained at the node of *L. pyrrhocoris*, *C. fasciculata* and *L. seymouri*. Numbers in brackets represent gene counts associated with each GO category. A multi-level pie chart was generated using the sequence filter set to 5 (minimal number of sequences a GO node must contain in order to be displayed). GO terms within all of the lowest nodes of the annotation graph that fulfill the filter condition are shown.

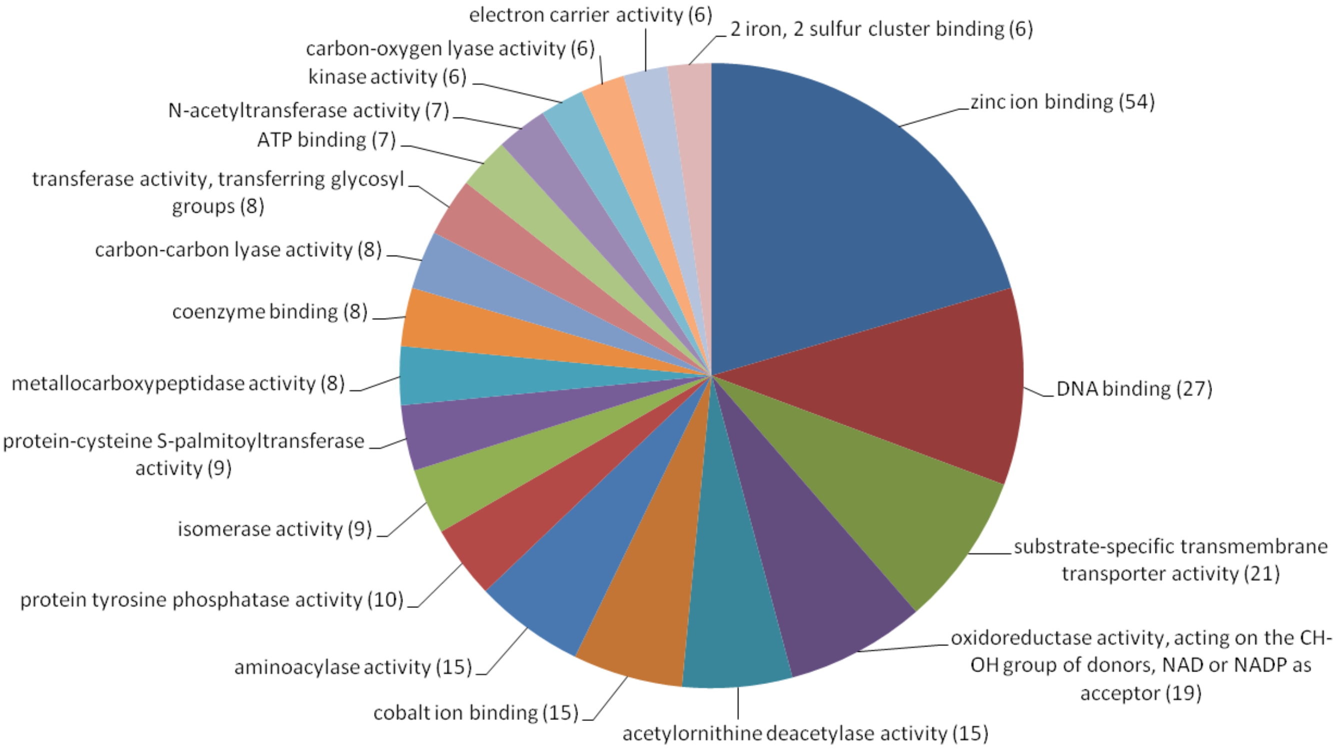

Suppl. Fig. S23: Gene family gains/losses for genes showing at least 1.5-fold up-regulation in *L. seymouri* at 35°C compared to 23°C and in a *L. major* virulent isolate compared to an avirulent one mapped on the tree of kinetoplastids with Dollo parsimony algorithm.

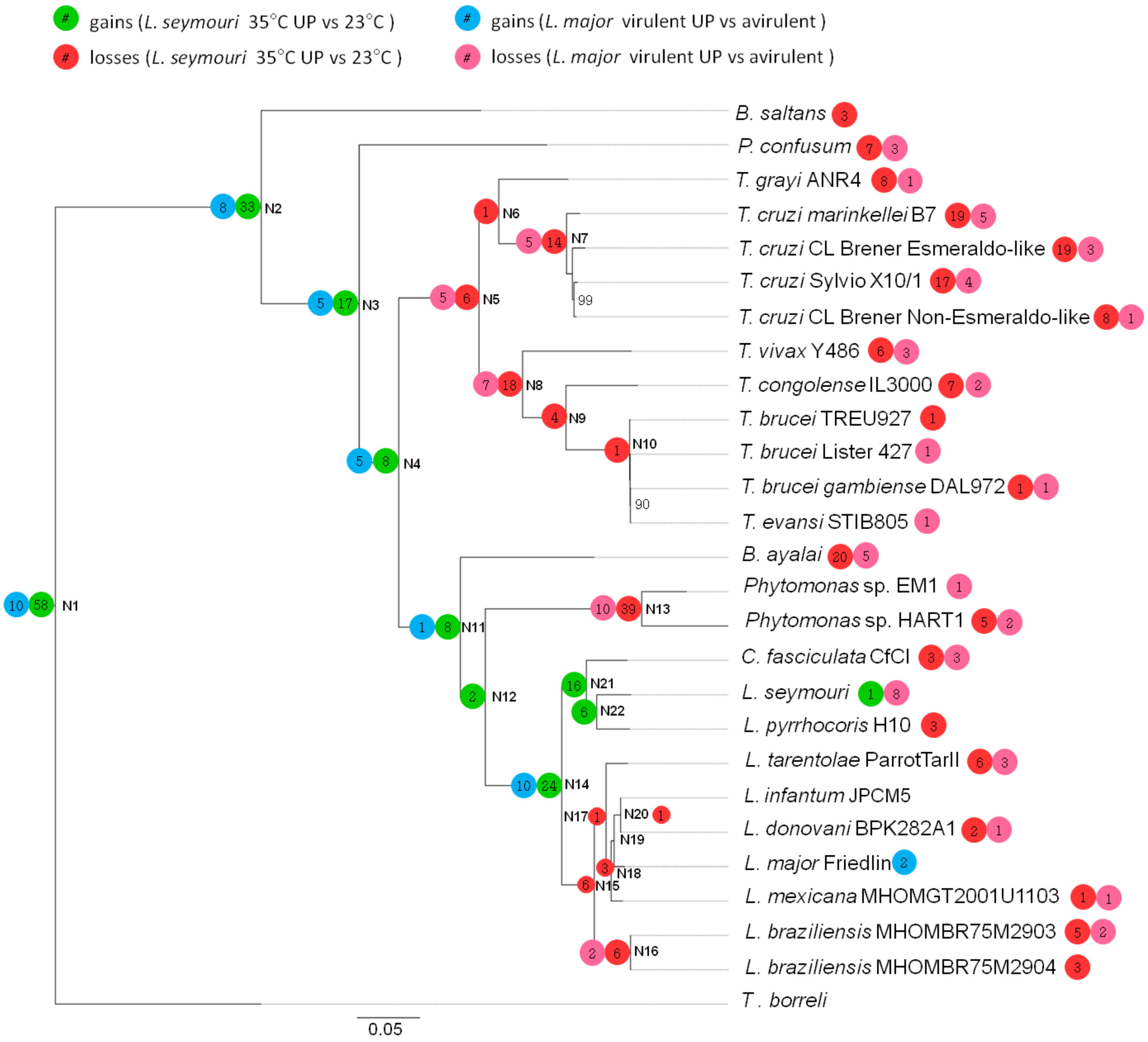

Suppl. Fig. S24: Gene family gains/losses for genes showing at least 1.5-fold up-regulation in *L. mexicana* metacyclics compared to procyclics and in amastigotes compared to metacyclics mapped on the tree of kinetoplastids with Dollo parsimony algorithm.

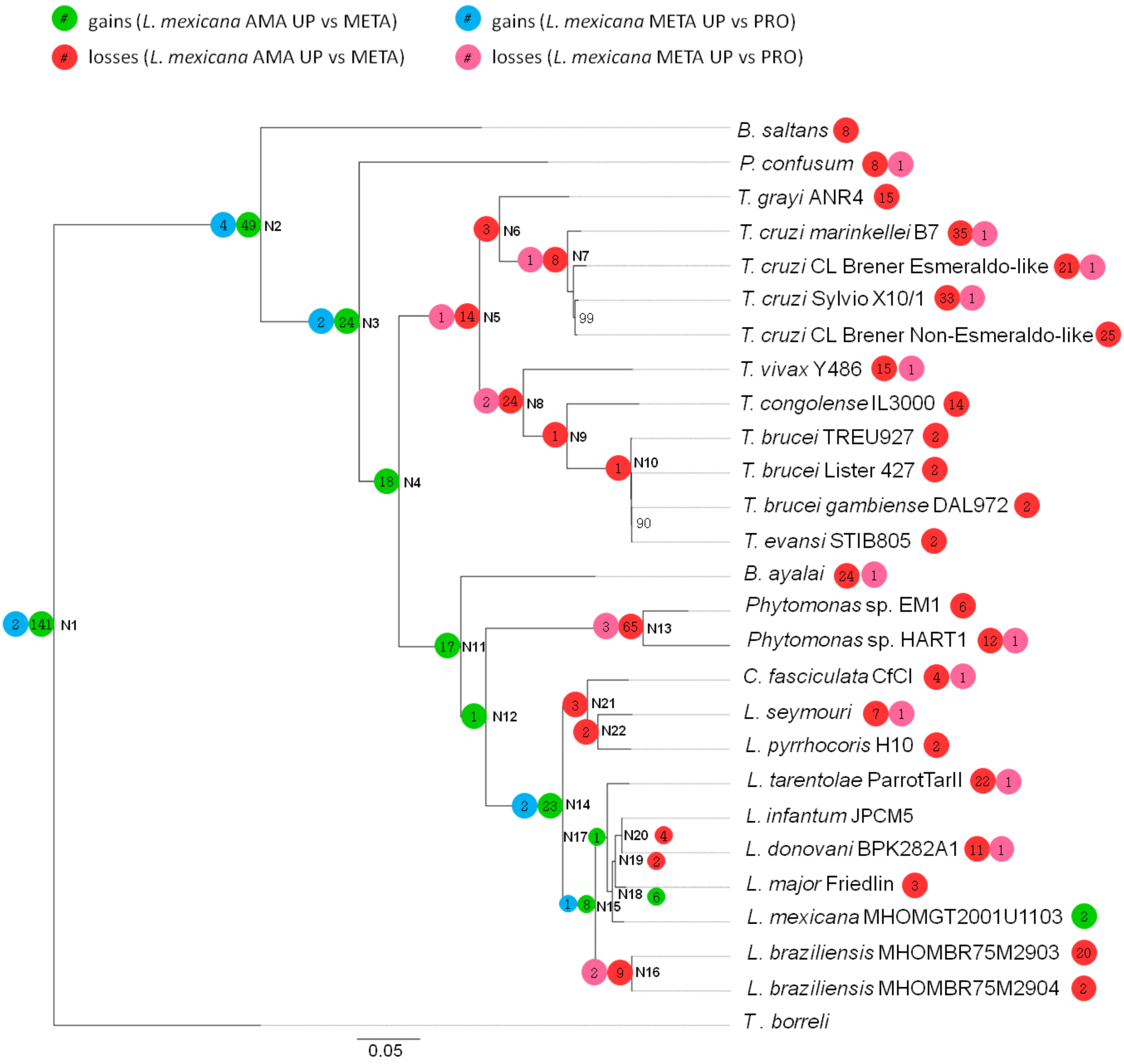

Suppl. Fig. S25: Gene family gains/losses for genes showing at least 1.5-fold up-regulation in *L. mexicana* amastigotes compared to procyclics mapped on the tree of kinetoplastids with Dollo parsimony algorithm.

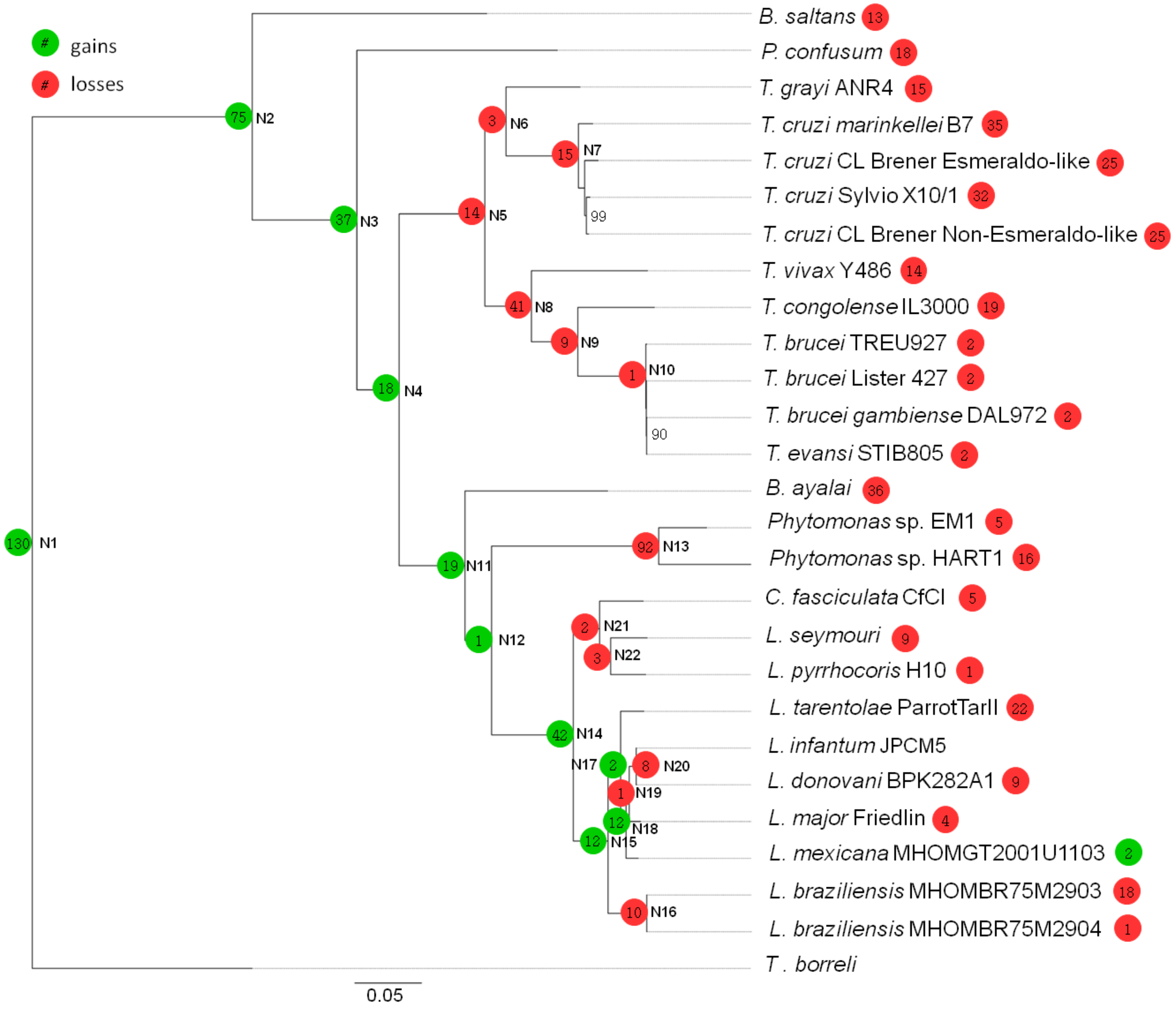

Suppl. Fig. S26: Cladogram of kinetoplastids with gene family gains/losses for 47 known *Leishmania* virulence factors mapped using Dollo parsimony algorithm. Some of the proteins analyzed belonged to the same OGs, therefore the number of groups mapped on the tree is lower than the actual number of virulence factors. OGs gained at the Leishmaniinae node were annotated as casein kinase 1 (CK1) isoform 4, cysteine protease b (cpb), autophagin 8, biopterin transporter 1 (BT1) and a hypothetical protein. CK1 plays an important role in signaling pathways and affects cell differentiation, proliferation and chromosome segregation. *Leishmania* genomes contain six CK1 isoforms, only one of which is specific for *Leishmania* spp. and *C. fasciculata* (the other isoforms have orthologs in other trypanosomatids). *L. donovani* over-expressing CK1 isoform 4 produced significantly higher infections of mouse peritoneal macrophages compared to wild-type parasites[2]. Notably, CK1 isoform 4 is up-regulated in *L. mexicana* amastigotes and metacyclics compared to procyclics according to *L. mexicana* RNA-seq data in this study. Another OG, cpb, plays an important role in suppressing Th1 host immune response. Mice infected with a *L. mexicana* cpb null mutant initially developed lesions which, however, were subsequently healed in a Th1-dependent manner[3]. Autophagin 8 is necessary for *Leishmania* life cycle progression and participates in the response to starvation[4]. *Leishmania* species are pterin auxotrophs and rely on transport of pterin from the environment using biopterin transporters (BTs). *L. donovani* with ablated BT1 gene showed a much lower capacity for inducing infection in mice than the wild-type parasites[5]. Notably, a knock-out of the hypothetical protein gained at the Leishmaniinae node in *L. donovani* caused a 200-fold decrease in virulence[6].

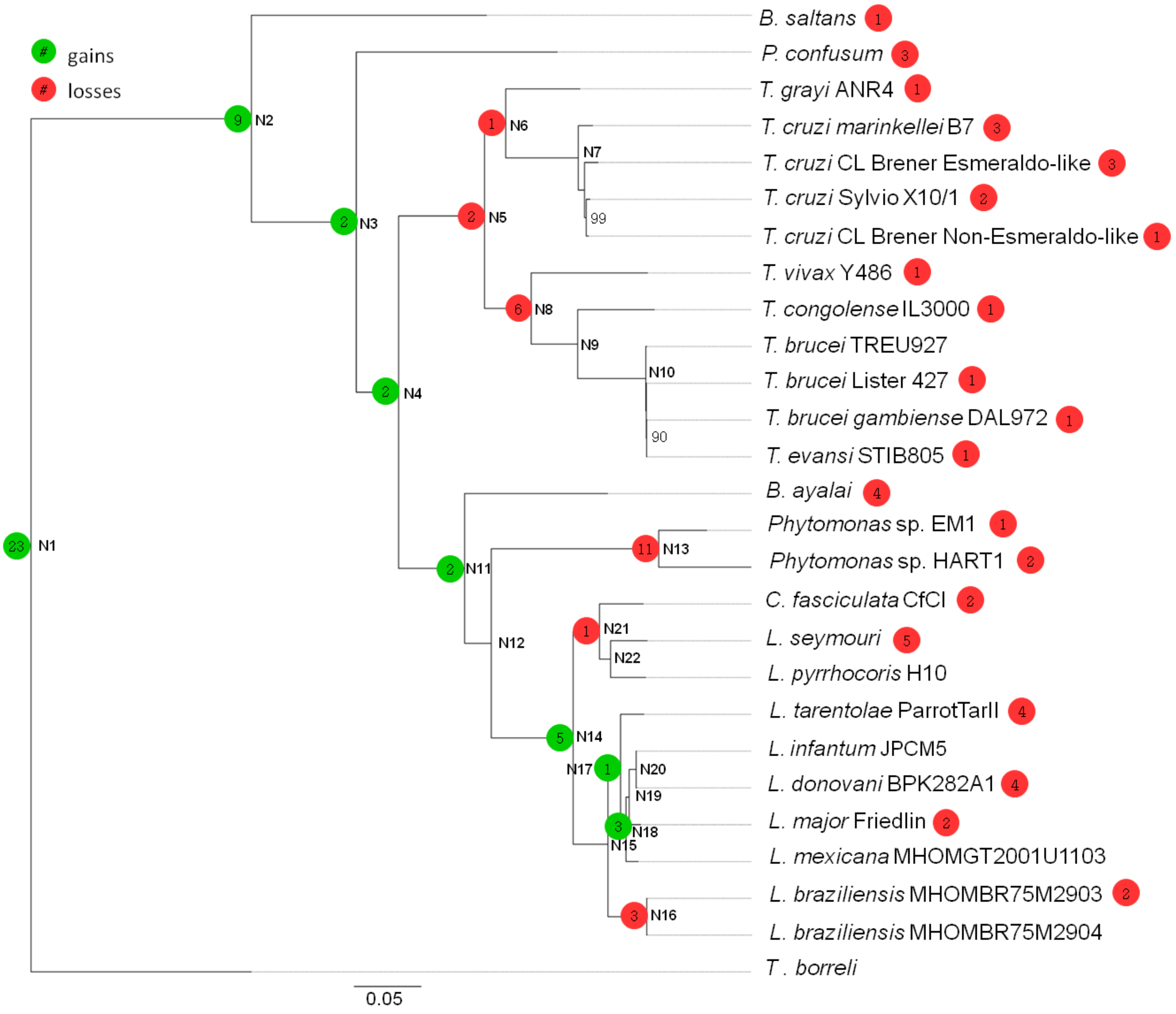

gains
losses
B. saltans 1
P. confusum 3
T. grayi ANR4 1
T. cruzi marinkellei B7 3
T. cruzi CL Brener Esmeraldo-like 3
T. cruzi Sylvio X10/1 2
T. cruzi CL Brener Non-Esmeraldo-like 1
T. vivax Y486 1
T. congolense IL3000 1
T. brucei TREU927
T. brucei Lister 427 1
T. brucei gambiense DAL972 1
T. evansi STIB805 1
B. ayalai 4
Phytomonas sp. EM1 1
Phytomonas sp. HART1 2
C. fasciculata CfCl 2
L. seymouri 5
L. pyrrhocoris H10
L. tarentolae ParrotTarII 4
L. infantum JPCM5
L. donovani BPK282A1 4
L. major Friedlin 2
L. mexicana MHOMGT2001U1103
L. braziliensis MHOMBR75M2903 2
L. braziliensis MHOMBR75M2904
T . borreli
N1 23
N2 9
N3 2
N4 2
N5 2
N6 1
N7
N8 6
N9
N10
N11 2
N12
N13 11
N14 5
N15 3
N16 3
N17 1
N18
N19
N20
N21 1
N22
99
90
0.05

Suppl. Fig. S27: Sequence variation at non-synonymous (red) and synonymous (blue) sites in protein coding genes of *L. pyrrhocoris.*

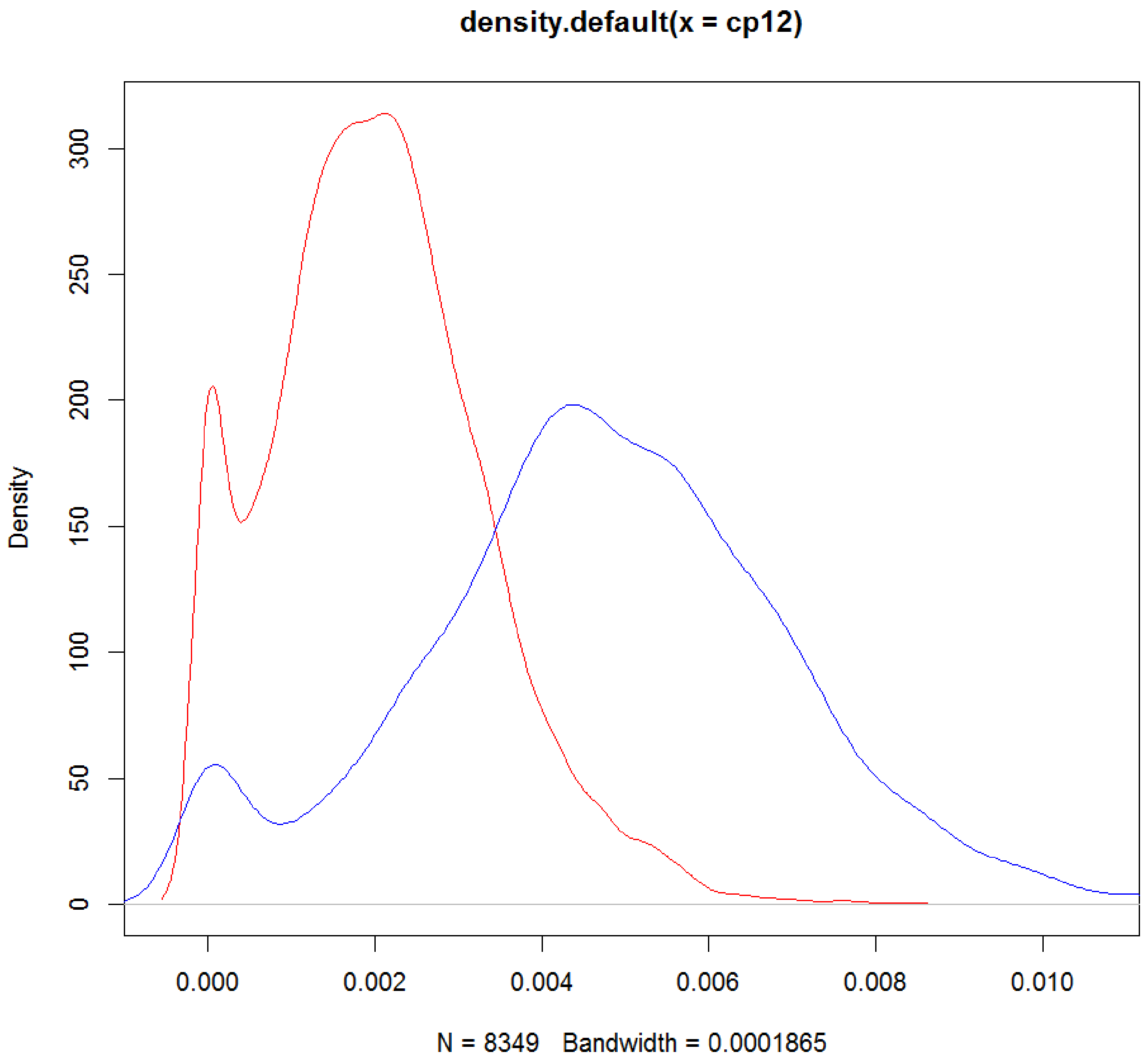

Suppl. Fig. S28: Distribution of single nucleotide polymorphisms across the genome of *Leptomonas pyrrhocoris*. Assembled scaffolds are depicted on top.

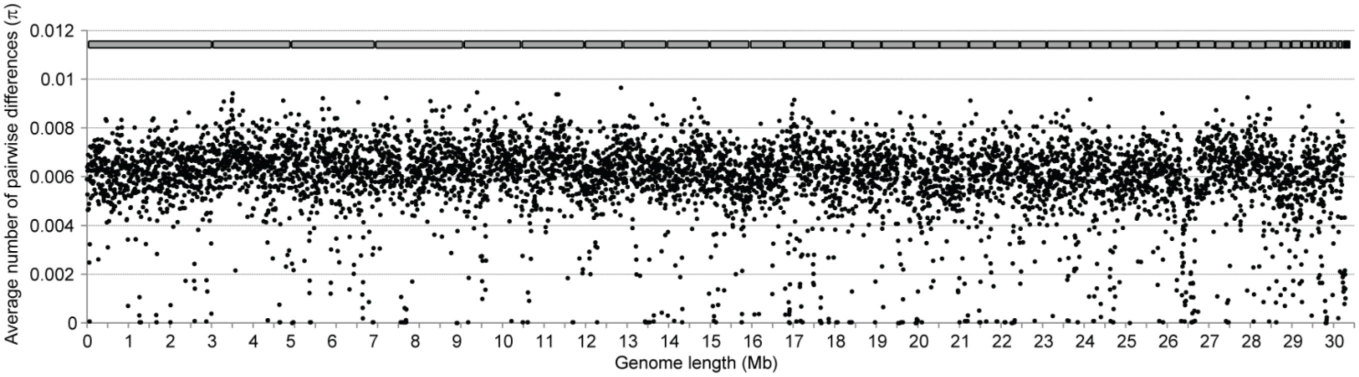

Suppl. Fig. S29: Gene family gains/losses mapped on the tree of kinetoplastids using Dollo parsimony algorithm, showing only OGs under positive selection in *Leptomonas pyrrhocoris*. The percentages near the nodes represent the proportion of OG gains for positively selected genes among all the OG gains at a certain node.

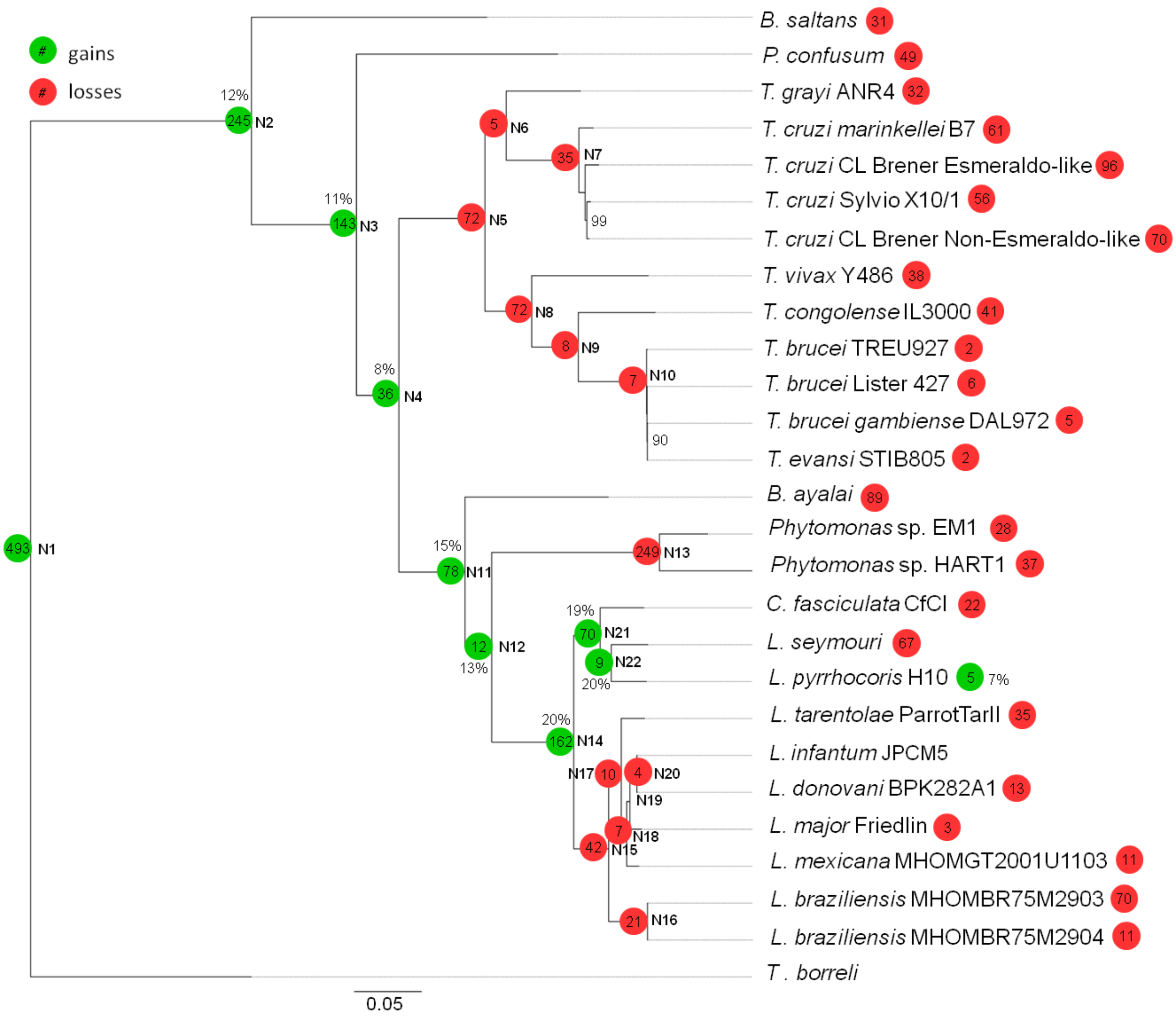

Supplementary Notes

1. Synteny analysis

Synteny can be a source of valuable information about selective forces acting on the genome structure. In particular, knowledge of synteny can be used for inferring gene functions (homologous genes having syntenic positions in genomes of different species are likely to retain similar functions), evolutionary history of genomes and, in addition, for refining existing genome assemblies[7,8]. Earlier studies demonstrated a striking conservation of gene order in the genomes of dixenous trypanosomatids of the genera *Trypanosoma*, *Leishmania*, and *Phytomonas*[9,10]. It was proposed that selection pressure supports the organization of genes into clusters, in which replication and transcription are co-directional, providing a possible explanation for the conservation of gene order in trypanosomatids.

The overall level of synteny in *L. pyrrhocoris* as compared to other trypanosomatids was studied using the reference dataset of 17 trypanosomatid species (Suppl. Table S1). Syntenic regions were inferred and visualized using SyMAP v.4.2[8] with the following settings: minimum size of sequence to load, 10 kbp; minimum number of anchors required to define a synteny block, 7; overlapping (or nearby) synteny blocks were automatically merged into larger blocks, and only the larger block was kept if two synteny blocks overlapped on a chromosome. Summary synteny statistics for 44 *L. pyrrhocoris* scaffolds longer than 10 kb and corresponding scaffolds/chromosomes of 9 selected species are shown in Suppl. Table S2. Analyzed scaffolds of *L. pyrrhocoris* account for 99.8% of its genome length and contain 10,121 out of its 10,148 genes. Between 74 and 96% of 'anchors' (pairwise alignments) in *L. pyrrhocoris* genome are located within synteny blocks in different inter-species comparisons, with the highest percentage in comparisons with *L. braziliensis* and *L. major* (Suppl. Table S2). This fact reflects a relatively short evolutionary distance between *L. pyrrhocoris* and *Leishmania* spp. and a good quality of their genome assemblies, as compared to assemblies of *L. seymouri* and *C. fasciculata*, even closer relatives of *L. pyrrhocoris*. The proportion of anchors intersecting gene annotation (Suppl. Table S2) is 95 to 99%. As expected, high overall levels of synteny between the compared genomes are mainly determined by homology between coding rather than intergenic regions. The highest proportion of genes within synteny blocks in the *L. pyrrhocoris* genome (90%) was found when aligned to scaffolds of its close monoxenous relative, *L. seymouri*. The fraction of genes retained within syntenic blocks gradually decreases from 77 to 22 % when the *L. pyrrhocoris* genome is compared to *C. fasciculata*, *L. major*, *L. braziliensis*, *B. ayalai*, *Phytomonas* sp., *T. cruzi*, and *T. brucei* genomes (Suppl. Table S2). The pairwise synteny between *L. pyrrhocoris* and three major model trypanosomatids (*C. fasciculata*, *L. major* Friedlin, and *T. brucei* TREU927) is

schematically represented in Suppl. Figs. S1-S3. A higher level of synteny is observed between *L. pyrrhocoris* and *L. major* Friedlin rather than its closer monoxenous relative *C. fasciculata*. This can be explained by the quality of the *C. fasciculata* genome assembly being lower than that of *L. major*. Remarkably, 15 scaffolds of *L. pyrrhocoris* (of 44 scaffolds >10kb in length) align to the chromosomes of *L. major* end-to-end and therefore do not show any rearrangements during the evolution of both species. These scaffolds probably represent completely assembled chromosomes of *L. pyrrhocoris*.

The syntenic context of 441 monoxenous-specific genes was also studied. We considered a gene as monoxenous-specific if it was shared between at least two monoxenous trypanosomatids and missing in all dixenous species according to our gene content analysis. By manual inspection of SyMAP results we found that *L. pyrrhocoris* scaffolds containing monoxenous-specific genes (LpyrH10_01 through LpyrH10_39) shared extensive regions of homology with 36 chromosomes of *L. major* Friedlin and *L. braziliensis* MHOM/BR/75/M2904. The regions of homology mentioned above were further analyzed in detail: pairwise TBLASTX search (E-value cut-off 10^{-5}) was conducted for *L. pyrrhocoris* scaffolds containing monoxenous-specific genes and corresponding *L. major* and *L. braziliensis* chromosomes. The results of BLAST were visualized and inspected manually using Artemis Comparison Tool[11]. Around 75% of monoxenous-specific genes were located within syntenic blocks and did not interrupt large-scale synteny of the genomes compared.

2. Metabolic pathways

Glycolysis and glycosomes

Genomic information confirms that *L. pyrrhocoris*, as all other trypanosomatids, has a fully operational glycolytic pathway (Suppl. Table 3). In addition to a classic cytosolic ADP-dependent pyruvate kinase, *L. pyrrhocoris* contains a gene for a second pyruvate-producing enzyme, the glycosomal pyrophosphate-dependent pyruvate:phosphate dikinase, also found in the glycosomes of the other trypanosomatids. *L. pyrrhocoris* is predicted to consume the hexose sugars glucose, fructose, mannose and galactose as well as the pentose sugars ribose, xylulose and ribulose. The predicted end products of carbohydrate metabolism, both aerobic and anaerobic, are succinate, pyruvate, acetate and carbon dioxide as well as minor quantities of ethanol and D-lactate.

It has been shown in *Leishmania* that the accumulation of triosephosphates, generated in the glycolytic pathway, may result in spontaneous dephosphorylation of some dihydroxyacetone phosphate and formation of a toxic metabolite methylglyoxal[12]. Methylglyoxal is inactivated by concerted action of the enzymes glyoxalase I, glyoxalase II, and D-lactate dehydrogenase, which were detected in the *L. pyrrhocoris* genome. Glyoxalase II is present in all kinetoplastids, whereas glyoxalase I and D-lactate dehydrogenase were not found in the African trypanosomes and *Phytomonas* spp.

Although no direct experimental evidence exists for the presence of glycosomes in *Leptomonas* spp., genomic information indicates that the early part of the glycolytic pathway, from hexokinase to phosphoglycerate kinase, localizes to glycosomes. Peroxisome-targeting sequences (PTS1 and PTS2) responsible for the import of proteins inside glycosomes are attached to either the C- or the N-termini of a number of these glycolytic enzymes (i.e. hexokinase, glucose-6-phosphate isomerase, fructose-bisphosphate aldolase, glyceraldehyde-phosphate dehydrogenase and phosphoglycerate kinase). Moreover, the identification of 11 genes coding for peroxins, proteins involved in glycosome biogenesis, and two glycosomal ABC transporters (GAT1 and GAT2) point to the presence of glycosomes in *L. pyrrhocoris*. In addition, *L. pyrrhocoris* genome encodes a catalase, a typical biochemical marker of the peroxisomes of most eukaryotic cells[13].

Carbohydrate metabolism

Based on genome analysis, it is predicted that *L. pyrrhocoris* feeds mainly on carbohydrates present in the insect midgut. Genes coding for glucoamylase, α-glucosidase, invertase and trehalase are all present as multiple copy genes (Suppl. Table 4). This suggests that *L. pyrrhocoris* not only utilizes the hexoses present in the insect's meal as energy source, but also disaccharides, such as sucrose, trehalose and maltose. The presence of a multicopy gene coding for a β-glucosidase (among trypanosomatids occurs only in *L. pyrrhocoris*, *L. seymori*, *C. fasciculata*, and *T. grayi*) that acts upon β1→4 bonds linking two glucose or glucose-substituted molecules, or functions as an exocellulase, together with the presence of chitinase that acts upon 1,4-β-poly-N-acetylglucosamine, indicates that plant and insect polysaccharides may also serve as important nutrients. Chitinase is also present in other representatives of Leishmaniinae as well as in *P. confusum* and *B.ayalai*. After polysaccharide hydrolysis the resulting monosacharides are interiorized, either by a sugar transporter, or by homologs of the *Leishmania* glucose transporter proteins *gt1*, *gt2*, and *gt3*. The above sugars can all be used as energy substrates because they feed directly into the glycolytic pathway.

Mitochondrial metabolism

Identification of the genes of the TCA cycle and all the subunits of the pyruvate dehydrogenase complex indicates the presence a functional mitochondrion in *L. pyrrhocoris*. Similar to *L. major* and *T. brucei*, *L. pyrrhocoris* has two isocitrate dehydrogenase (IDH) isoenzymes. Both are specific for NADP, rather than NAD. One of them is present in the mitochondrion and is of eukaryotic origin, while the other is of prokaryotic origin and is cytosolic, as in *Leishmania*. Thus, in all trypanosomatids analyzed so far, the typical catabolic mitochondrial NAD-dependent IDH has been replaced by an anabolic NADP-dependent isoenzyme, which functions in the reductive direction of isocitrate

formation with NADPH consumption. Isocitrate produced in the mitochondrion is utilized in the cytosol, either for fatty acid synthesis or for protection against oxidative stress[14] through NADPH it generates via the cytosolic NADP-IDH isoenzyme. The absence of a catabolic NAD-IDH isoenzyme renders it difficult, or even impossible, for *L. pyrrhocoris* to use its TCA cycle for the complete oxidation of pyruvate, or acetyl-CoA, to carbon dioxide and water. Instead, the function of the remaining TCA-cycle enzymes seems to be supplying the cell with necessary metabolic intermediates required in its biosynthetic pathways[15,16].

Amino acid metabolism

Capacity of *L. pyrrhocoris* for the synthesis of amino acids is limited to the non-essential ones plus threonine and methionine, similar to other trypanosomatids. Most genes coding for enzymes involved in the synthesis of these amino acids were identified (Suppl. Table 5). Notably, none of trypanosomatids has the capacity to synthesize lysine *de novo*. However, it has been shown that *Crithidia* spp. is able to transform the bacterial amino acid diaminopimelate (DAP) into lysine[17]. DAP is an ε-carboxy derivate of lysine and an important component of the peptidoglycan cell wall of some gram-negative bacteria. *L. pyrrhocoris* is also able to carry out this transformation since it possesses, just as *C. fasciculata*, DAP epimerase and DAP decarboxylase genes.

Proline and glutamic acid, very abundant amino acids of the insect midgut, are used by trypanosomatids as a major source of carbon and ammonia[18]. Proline is oxidized to glutamic acid by mitochondrial proline oxidase and delta-1-pyrroline-5-carboxylate dehydrogenase. Glutamic acid is also produced by transamination of many other amino acids. Interestingly, subsequent oxidative deamination of glutamic acid to the TCA-cycle intermediate 2-ketoglutarate is not possible because a gene for the classic mitochondrial NAD-dependent glutamate dehydrogenase (NAD-GDH) was not detected in *L. pyrrhocoris*. Instead, the *L. pyrrhocoris* genome encodes four tandemly linked and very similar alanine aminotransferase genes, one of which carries a mitochondrial transit peptide. This makes it likely that in the *L. pyrrhocoris* mitochondrion glutamate is converted to 2-ketoglutarate by transamination between glutamate and pyruvate. The resulting alanine is excreted as an important end product of amino-acid catabolism. Alternatively, NADP-GDH, a cytosolic enzyme previously reported for *T. cruzi* and *L. major*[19], could be responsible for the formation of 2-ketoglutarate, but this step would have to take place in the cytosol. Whatever the mechanism, 2-ketoglutarate, once formed, is either converted to succinyl-CoA and succinate by the forward TCA cycle, or by a reversed TCA cycle, comprising NADP-linked IDH and aconitase, is converted to isocitrate and citrate. Histidine is not metabolized by *L. pyrrhocoris*, similarly to most trypanosomatids. This contrasts with the situation encountered in *T. cruzi*, where four genes of histidine catabolism were identified. As observed

previously for *Trypanosoma* and *Leishmania* spp., the enzymes of the classic pathway of aromatic amino-acid oxidation are missing in *L. pyrrhocoris* as well. Interestingly, only *C. fasciculata* and *Leptomonas* spp. have the capacity to synthesize arginine from citrulline and aspartate due the presence of the genes encoding argininosuccinate synthase and argininosuccinate lyase.

Oxidative stress protection

Oxidative stress protection in trypanosomatids is based on trypanothione, adduct of one molecule of spermidine and two molecules of glutathione[20]. Genes encoding trypanothione reductase, trypanothione synthase, thioredoxin and tryparedoxin peroxidase were identified in the *L. pyrrhocoris* genome.

At least five superoxide dismutases (mitochondrial and cytosolic isoenzymes) and an iron/ascorbate oxidoreductase were also detected. *L. pyrrhocoris* shares with *L. seymouri* and *Crithidia* spp. a bacterial-type catalase, which was acquired by a recent event of lateral transfer, since bacterial and trypanosomatid homologues are still 70% identical. This is in agreement with earlier observations of high catalase activities in these organisms[21,22]. Peroxisomal β-oxidation results in the formation of large amounts of the reactive oen species (ROS) which are inactivated by superoxide dismutase and catalase. Apparently multiple anti-ROS enzymes in *L. pyrrhocoris* provide sufficient protection against ROS inside the organelle (Suppl. Table 6).

Reducing equivalents in the form of NADPH maintain the redox components in the reduced state and are provided by two isoforms of NADP-IDH and by the hexose-monophosphate pathway enzymes, glucose-6-phosphate dehydrogenase and 6-phosphogluconate dehydrogenase, present in both the cytosol and in glycosomes. A plant-like ascorbate peroxidase, as described for *T. cruzi* and *Leishmania* spp., was also detected in *L. pyrrhocoris* as well as in *B. ayalai*, *B. saltans, P. confusum, L. seymouri* and *C. fasciculata* (Suppl. Table 6).

3. Trafficking and signaling components

Membrane and protein trafficking

The intracellular transport system of trypanosomatids has major roles in immune evasion, and is the site of synthesis, modification and degradation of cell surface molecules[23]. Most trypanosomatid flagellates analyzed to date have endomembrane protein complements that are well conserved when compared with other lineages[10,24]. *L. pyrrhocoris* is no exception, as it has a complement of vesicle coat and tethering complex proteins, and SNAREs similar or identical to those that have been identified in *T. brucei*[1,25-28] (Suppl. Table S7 and Suppl. Fig. S6). Significantly, the improved sampling of trypanosomatid genomes allowed us now to identify putative orthologs in *L. pyrrhocoris* and other

trypanosomatids of some membrane trafficking proteins missed in previous surveys, for example the Exocyst subunit Sec5 or the TRAPPII subunit Trs120 (Suppl. Table S7). Similarly to the *Leishmania* spp., there is no p67 in *L. pyrrhocoris*, the major lysosomal protein of trypanosomes. *L. pyrrhocoris* possesses a full complement of the classic heterotetrameric adaptor protein complexes, AP-1 through AP-4 (Suppl. Table S7), whereas the recently defined related complexes AP-5 and TSET are apparently missing, as previously reported for *Leishmania major* and *T. brucei*[29,30]. The presence of AP-1 through AP-4 complexes in *L. pyrrhocoris* is unusual as *Leishmania*, *Phytomonas* and African trypanosomes secondarily lack one or other of these complexes, and hence suggests a configuration that is more complex and rather closely resembles the free-living *B. saltans* also equipped with all four complexes. However, since *T. cruzi* also retains AP-1 through AP-4 complexes, this feature is not exclusively associated with the monoxenous life cycle[31]. Similarly to the GTPase cohort, there is no obvious modification associated with the membrane trafficking system that is associated with a switch to the parasitic life style.

Small GTPases

The set of small (Ras superfamily) GTPases in *L. pyrrhocoris* is nearly identical to that in *Leishmania major*, whereas more pronounced differences exist between *L. pyrrhocoris* and other trypanosomatids analyzed (i.e. *Phytomonas* sp. EM1, *T. brucei*, and *T. cruzi*), primarily because of differential loss of some genes in different lineages (Suppl. Table S8). A salient feature of the *L. pyrrhocoris* small GTPase set is an ARF-like GTPase, retained only in *T. cruzi* of all other trypanosomatid species analyzed here (see ARL18 in Suppl. Table S8), that represents a novel uncharacterized gene conserved in diverse protists and apparently ancestral for eukaryotes (Eliáš et al, unpublished data). However, the functional significance of its presence/absence pattern in trypanosomatids remains unclear due to the lack of functional data from any organism. Nearly all of the small GTPases present in some trypanosomatids, yet missing from *L. pyrrhocoris*, seem to have been lost before the divergence of the Leishmaniinae and the *Phytomonas* lineages and independently in the *T. brucei* lineage, making the *T. cruzi* small GTPase set the most extensive (Suppl. Table S8). The GTPases specifically retained in the *T. cruzi* lineage include four RAB family members (Rab21B, Rab32, Rab32L, and RabX5) and one ARF-like GTPase (ARL8) that are all predicted to be functionally associated with endosomal and/or lysosomal compartments, suggesting a trend towards reducing the molecular complexity of the endocytic pathway in trypanosomatid evolution. Our analysis also confirms that the *bona fide* Rho GTPase, previously characterized from *T. cruzi* and noticed to be missing from *T. brucei* and *L. major*, is restricted to the *T. cruzi* lineage (Suppl. Table S8)[32,33].

In addition to gene loss as an important factor sculpting the small GTPase set in

trypanosomatids, evolutionary innovations via emergence of new paralogs have also played a role. For example, our analyses of the *L. pyrrhocoris* genome revealed a previously unnoticed novel protein conserved in all trypanosomatids and characterized by an N-terminal phospholipid-binding PH (pleckstrin homology) domain and a C-terminal divergent ARF-like GTPase domain separated by a long unique region (PH-ARL, Suppl. Table S8). The presence of an obvious ortholog in *B. saltans* but no eukaryotes outside kinetoplastids indicates that this protein evolved in the kinetoplastid lineage, perhaps to regulate a specific membrane trafficking pathway (implied from the domain architecture of this protein). Small GTPase gene duplications within trypanosomatids are rare; for example, a single, recent duplication specific for the *L. pyrrhocoris* lineage could be identified, resulting in a pair of Rab7 paralogs identical even at the nucleotide level. More frequent are duplications affecting genes encoding ARF GTPases, crucial factors of vesicle trafficking at the Golgi. Trypanosomatids were found to harbour two significantly different paralogs ARF1 and ARF2[34], which apparently emerged from a gene duplication preceding the trypanosomatid radiation and proved to be conserved in all species investigated (Suppl. Table S8). ARF1 then duplicated independently in *T. brucei* and *T. cruzi* lineages, resulting in nearly or completely identical paralogs, whereas another independent duplication occurred before the split of Leishmaniinae and *Phytomonas*, adding a paralog in *L. major* previously denoted ARF3 and retained also in *L. pyrrhocoris*. The evolutionary stability of the ARF1/ARF3 pair suggests that these two paralogs have become functionally specialized. Overall, the complement of small GTPases indicates a well conserved endomembrane system, when compared with other trypanosomatids, with no major indication of modification that accompany the transition from the mono- to dixenous life style.

4. Cell surface proteins

The promastigote surface antigen (PSA)-like genes were retrieved from the *L. pyrrhocoris* gene set using a canonical protein sequence from *L. major* (LmjF.12.0740) and TBLASTN. Translated nucleotide sequences for the PSA-like genes were aligned in ClustalW[35] and then manually edited in BioEdit v7.1.3[36]. This produced a 168-character multiple sequence alignment of amino acid sequences, corresponding to the non-repetitive N-terminal domain of GP46 and phosphoproteoglycan (PPG) proteins. The remaining portions of the predicted proteins could not be aligned due to their non-homologous repeats. The phylogeny was estimated using the LG + Γ model in PhyML[37] with 100 non-parametric bootstraps and in MrBayes under the following settings: Nruns=4; Ngen=5,000,000; samplefreq=500 and default prior distribution[38].

A full GPI-anchor biosynthetic pathway and protein transfer system is present, consistent with the presence of multiple orthologs of gp63 and gp46, which likely populate the cell surface (Suppl.

Fig. S7). Furthermore, the presence of a β-galactofuranose transferase ortholog suggests that *L. pyrrhocoris* is probably capable of synthesizing GIPLs and, probably, a full LPG. Furthermore, multiple copies of genes encoding secreted- and membrane-bound acid phosphatase are also present. These proteins can act as acceptors for LPG-related repeats, providing more evidence for an extensive, *Leishmania*-like host interaction. There is no evidence for mucins, VSG or ISGs, while 19 amastin-like sequences were recovered from the *L. pyrrhocoris* genome. When combined with homologs from related trypanosomatid genomes, phylogenetic analysis of these sequences shows that they include orthologs of all amastin sub-families except for δ-amastin, which remains specific to *Leishmania* spp. (Suppl. Fig. S8).

One of the largest *Leishmania*-specific clusters in gene cluster analyses includes the promastigote surface antigen (PSA) or gp46 genes, suggesting that the family was elaborated after the origin of *Leishmania*. To examine this, we estimated maximum likelihood and Bayesian phylogenies for 34 PSA-like sequences present in *L. pyrrhocoris* and their homologs in a range of related species. Their phylogeny can be subdivided into a series of robust lineages with widespread distribution (Suppl. Fig. S9). Seven of these are represented in the *L. pyrrhocoris* genome, including the PPG genes (grey shading), while other lineages are present in both the investigated flagellate and *C. fasciculata*, but absent from the *Leishmania*. Interestingly, the divergent outgroup sequence from *T. cruzi* has an ortholog in *L. pyrrhocoris* but no other species. Most notably, the tree includes a clade, which was found exclusively in *Leishmania* species (shaded red in Suppl. Fig. S9). This clade contains genes located in *L. major* and other species at two tandem gene arrays, on chromosomes 12 and 31, respectively. The absence of orthologs to these arrays in *L. pyrrhocoris* and *C. fasciculata* demonstrates that these are *Leishmania*-specific derivations of this family of developmentally regulated, cell-surface glycoproteins playing a possible role in anterior migration through the insect host.

5. Kinase candidates

A draft kinome containing candidate members of the protein kinase superfamily in *Leptomonas* was predicted using Kinannote[39]. The predicted kinome sequences were aligned using ClustalW[35] with the Kinannote-predicted draft kinomes from *T. cruzi* (CL Brener Esmeraldo-like and CL Brener Non-Esmeraldo-like genomes), *L. braziliensis* and *L. major* (all downloaded from TriTrypDB v. 6.0). A ClustalW NJ tree was built from the alignment. Kinannote produces two kinome predictions - the draft kinome and a slightly smaller kinome which contains fewer IDs and more refined classifications. The NJ tree IDs were annotated to include the more refined Kinannote-generated kinase classifications. The remaining IDs, mostly classed as 'subthreshold' or 'protein kinase subdomain-containing protein' in the

draft kinome, were annotated to include these draft kinome classifications. The NJ tree was further annotated to indicate sequences that were found to be a reciprocal best BLAST hit with any of the *Leptomonas* draft kinome candidates. Clusters of orthologous kinases were identified in the NJ tree by (a) the bootstrapped branching pattern of the included species in the cluster, (b) rbh BLAST results between cluster members and (c) shared Kinannote classifications. *Leptomonas* sequences were typed as: 'conserved' if located in a cluster of orthologous kinases (clusters in which all member sequences were classified as 'subthreshold' by Kinannote were ignored), 'unique' if no orthologue was found in the dataset (unique sequences that were classified as 'subthreshold' by Kinannote were ignored), '*Leishmania*-only' if no *T. brucei* or T. *cruzi* orthologue candidates were identified, 'expanded' if more than one version was located in a cluster of orthologous kinases, and 'missing' if no representative was found in a cluster of orthologous kinases.

There are approximately 160 kinases in the *T. brucei* kinome, and a similar number have been described in *L. major* and *T. cruzi*[40]. Some of these have been implicated in differentiation and cell cycle progression of *Leishmania* and *Trypanosoma* spp. *via* changes between the phosphoproteomes of different life stages[41,42], and several are considered as potential drug targets[43]. Trypanosomatids appear to lack receptor-like tyrosine kinases, but all the other major kinase classes are well represented. There is a clear evidence for lineage-specific expansions or contractions within these families, making assignments based on homology challenging[40]. The number of kinases in *L. pyrrhocoris* is similar to that of other trypanosomatids, with some notable expansions and losses. This implies that the vast majority of these shared kinases have little to do with differentiation *per se*, or that they may play roles during development within the insect vector (Suppl. Table S9). *L. pyrrhocoris* has a small number of expansions within the CAMKK, NEK and STE/STE11 kinase families, while there are also several kinases that are absent from *L. pyrrhocoris* and *Leishmania* spp. However, these represent less than 8% of the total repertoire detected in our analysis. Significantly, two kinases assigned recently as having roles in delaying differentiation between the mammalian infective and insect stages of *T. brucei*, RDK1 (Tb927.11.14070) and RDK2 (Tb927.4.5310), are retained in *L. pyrrhocoris* (LpyrH10_02_0960 and LpyrH10_29_1130, respectively). This suggests that some of the signaling capability required for differentiation arose from pathways already present in monoxenous species.

6. Patterns of gene gains and losses

Ninety nine OGs were gained at the *Leishmania* node (Fig. 2), and only a few of those OGs were subsequently lost in several *Leishmania* spp. (with the largest number of losses in the lizard parasite *L. tarentolae*), which suggests a particular importance of these novel gene families for the *Leishmania* dixenous life cycle. The most frequent and specific gene ontology (GO) terms connected to the gains at

the basal *Leishmania* node are associated with proteolysis (Supplementary Fig. S12). In *Leishmania* spp., peptidases are key effectors of cell invasion, survival in macrophages and immune modulation[44]. For instance, one of the best known virulence factors, zinc metalloprotease GP63, has multiple functions during the establishment of *Leishmania* infection in the vertebrate host[45-47] and also in the insect vector. GP63 orthologs appeared earlier in evolution but are divergent enough in *Leishmania* to be recognized as a separate entity in the OrthoMCL analysis in this study (average identity between *Trypanosoma*-specific and *Leishmania*-specific GP63 orthologs is ~25%, whereas within the *Leishmania*-specific group it is 72%). Another frequent GO term assigned to OGs gained at the *Leishmania* node, 'phosphate-compound containing metabolic process', includes phosphatases and adenylate cyclases and is possibly connected to signal transduction. Additional signaling pathways have evolved in *Leishmania* spp. to enable their survival in the changing environment of the vector and vertebrate host organism[48]. However, most OGs gained at the *Leishmania* node contain proteins with no functional annotation (87 of 99 OGs, Fig. 2), which is reflected by the paucity of specific GO terms in Supplementary Fig. S12.

Nine gene families were expanded and 53 OGs underwent contraction at the *Leishmania* node (Supplementary Figs. S13-S15). Among functions associated with the expanded gene families, the following were most frequent according to GO term analysis: 5S rRNA binding within the ribosome; nucleoside diphosphate kinase; Rab GTPase activator; stearoyl-CoA 9-desaturase (Supplementary Fig. S14). Overall, 307 OGs were lost at the basal *Leishmania* node (see a pattern of gene gains for these OGs in Supplementary Fig. S16). The set of GO terms assigned to these gene families suggests the loss of some mobile genetic elements in *Leishmania* genomes (GO terms 'DNA recombination', 'DNA integration', 'RNA-dependent DNA replication') and the loss of metabolic versatility in *Leishmania*, reflected in the following GO terms: glycolytic process, coenzyme metabolic process, and aerobic respiration. For example, *Leishmania* spp. cannot synthesize some key co-factors such as biotin, coenzyme A, FMN and FAD, NAD, NADP, pyridoxal phosphate and thiamine pyrophosphate.

According to our analysis, a rather large number of OGs, 821, was gained at the Leishmaniinae node (Supplementary Fig. S17), associated with a wide variety of processes according to GO term analysis. The following 'biological process' terms were most frequent: protein phosphorylation, autophagy, protein glycosylation, transmembrane transport, cell redox homeostasis, translational elongation, carbohydrate transport, nucleic acid hydrolysis, small GTPase mediated signal transduction, long-chain fatty acid biosynthesis, and terms related to amino acid metabolism (Supplementary Fig. S17). The following 'molecular function' terms were most frequent: galactosyltransferase, heme binding, calcium binding, electron carrier, fatty acid elongase, translation elongation factor, sucrose alpha-glucosidase, calcium-dependent cysteine-type endopeptidase,

endonuclease, fatty-acyl-CoA binding, triglyceride lipase (Supplementary Fig. S18-S20). Taken together, these terms suggest changes in a variety of signal transduction and regulatory processes, in autophagy, in glycosylated proteins of the cell surface, translation, fatty acid biosynthesis, and in amino acid metabolism. *L. major* null mutants for several autophagy pathway genes showed reduced capacity for differentiation between life cycle forms and decreased ability to infect macrophages both *in vitro* and *in vivo*[49]. The glycosylation machinery is vital for *Leishmania* spp., which synthesize various glycoconjugates including membrane-attached glycosylphosphatidyl-inositol (GPI) anchored lipophophoglycan (LPG) and proteophosphoglycan (PPG) along with secreted forms of PPGs[50-52]. The cell surface of trypanosomes is dominated by other glycoconjugate structures: variant surface glycoproteins or procyclic acidic repetitive proteins in *T. brucei*, and glycosylinositolphospholipids, mucins or lipopeptidophosphoglycan in *T.cruzi* (depending on the life cycle stage)[53]. These data therefore indicate that the LPG/PPG system greatly predates the evolution of the dixenous parasitic life style. Hence the LPG/PPG system can be considered an adaptation mainly to arthropod hosts. Emergence of novel gene families of transmembrane transporters and proteins participating in amino acid metabolism at the Leishmaniinae node is associated with the fact that sugars and amino acids (particularly proline, threonine, arginine, and glutamic acid) serve as important energy substrates in the midgut of the insect vector[54]. Several novel genes participating in fatty acid biosynthesis emerged at the Leishmaniinae node. As in *T. brucei*, in the absence of cytosolic fatty acid synthase I, *Leptomonas* uses mitochondrial fatty acid synthase II and an extended set of elongases for *de novo* synthesis of fatty acids[55]. It was reported previously that *L. major* possesses orthologs of *T. brucei* elongases as well as a set of eight additional genes for elongases possibly involved in elongating saturated fatty acids[55]. In our OrthoMCL analysis five out of eight 'additional' elongases of *L. major* clustered separately from trypanosomal elongases and were also found in *Leptomonas* spp., and therefore are unique for the Leishmaniinae clade.

On the other hand, 372 gene families gained at the basal node of monoxenous *C. fasciculata*, *L. pyrrhocoris*, and *L. seymouri* (Fig. 2) are assumed to be associated with the monoxenous life style, although the ability of the latter species to survive at elevated temperatures and therefore its potential to occasionally infect warm-blooded hosts was shown[56,57]. GO terms associated with oxidation-reduction processes were assigned to a majority of proteins gained at this node (Supplementary Figs. S21, S22). Manual inspection of these sequences revealed that the term ‘catalase activity’ was assigned to several ones. Catalase is a heme-containing enzyme playing an important role in cellular protection against radical oxygen species (ROS) and is considered to be a typical biochemical marker of peroxisomes in most eukaryotic cells. Catalase is restricted to some monoxenous trypanosomatids while all dixenous species are catalase-negative[21,22,58]. Other most frequent functions gained at the

Leptomonas/*Crithidia* node include: M32 carboxypeptidases, glucose transporters, argininosuccinate lyases, protein tyrosine phosphatases, N-acyl-L-amino acid amidohydrolases and aminoacylases, protein-cysteine S-palmitoyltransferases. M32 carboxypeptidases were shown to be involved in peptide catabolism in *Leishmania*[59]. N-acyl-L-amino acid amidohydrolases and aminoacylases function in amino acid metabolism and proteolysis[60]. Among trypanosomatids, only *C. fasciculata* and *L. pyrrhocoris* were predicted to have the capacity to synthesize arginine from citrulline and aspartate due the simultaneous presence of genes encoding argininosuccinate synthase and argininosuccinate lyase in their genomes. Protein tyrosine phosphatases play an important role in regulation of various signaling processes, and it was suggested that diverse environments encountered at different stages of trypanosomatids' life cycles might have facilitated the development of a set of trypanosomatid-specific phosphatases, in addition to orthologs found in other eukaryotes[48].

Supplementary Methods

Genome assembly and annotation

The initial genome assembly of *Leptomonas pyrrhocoris* H10 was made using GS De Novo Assembler (Newbler) v. 2.6 from high-throughput sequencing reads of the 454 FLX platform (Roche): 1/ about 2 million single reads, average length 364 nt, 25x coverage of the genome assembly; 2/ about 1.1 million mate pair reads, average length 183 nt, insert size range 1,500 - 4,500 bp, 14x coverage. See statistics for the initial assembly below: number of scaffolds, 146; maximum scaffold length, 2,319,204 bp; total scaffold length, 29 Mbp; average scaffold size, 199 kbp, N50 scaffold size, 483 kbp; number of contigs in scaffolds, 1,303; average scaffold contig size, 21.6 kbp; N50 scaffold contig size, 57.7 kbp.

Subsequently, we performed manual assembly finishing, i.e. gap closing through analysis of the graph of alternative contig connections produced by Newbler. That allowed us to assemble some heterozygous and repetitive regions: 69% of gaps were closed, and 7% partially closed; 87% of 'N's were removed; 55% of 146 scaffolds were joined to at least one other scaffold, producing 96 scaffolds.

At the next step, insertion/deletion sequencing errors in homopolymer tracts, rather frequent in assemblies based on the 454 technology, were corrected with the iCORN v. 0.97 software (with default settings), using the following library of paired-end Illumina HiSeq genomic reads: about 38 million reads; read length 100 nt; insert size range 380-480 nt; 127x coverage.

In order to improve assembly quality still further, *L. pyrrhocoris* scaffolds were aligned on the high quality assembly of *L. major* Friedlin using MUMmer v. 3.23, and paths in the contig graph between *Leptomonas* scaffolds aligned end-to-end on a *Leishmania* chromosome were checked. In order to avoid bias favoring genome synteny with *L. major*, *Leptomonas* scaffolds were joined only if a

valid path in the contig graph of the original *Leptomonas* genome assembly existed, eventually producing 62 scaffolds. At this stage, the assembly was subjected to the first pass of gene annotation (see below). Illumina MiSeq transcriptome reads, after adapter and quality trimming in CLC Genomics Workbench v.7.0 (CLC Inc, Aarhus, Denmark), were aligned to the genome using Bowtie2 v.2.2.5[61] with '--end-to-end' and '--very-sensitive' options, and short gaps (<25 bp in length) within annotated genes were closed using this read mapping. The gaps were replaced with the sequence of the mapped RNA-seq reads or, if they were missing in RNA-seq reads, completely removed. Next, 10 contigs (from 3 kbp to 100 kbp in length) missed from the original assembly due to heterozygous deletions, were placed manually into the final assembly. Here are statistics for the final genome assembly: 60 scaffolds; maximum scaffold length, 2,995,728 bp; scaffold N50, 910,096 bp; total assembly length, 30.4 Mbp

Augustus v. 2.5.5[62] was used to annotate the final genome assembly of *L. pyrrhocoris*. Prediction accuracy of Augustus was improved by retraining using a training set of *L. pyrrhocoris* conserved proteins. In brief, *de novo* assembled scaffolds were searched against proteins in the TriTrypDB v. 7.0 database (BlastX E-value ≤ 10^{-5}) and best BLAST hits were chosen based on the following criteria: a) E-value ≤ 10^{-30}, b) hit length longer than 80 amino acids (aa), c) percent identity higher than 40. Subsequently, transcriptome reads after adapter and quality trimming in CLC Genomics Workbench v.7.0 (CLC Inc, Aarhus, Denmark) were aligned to the genome using Bowtie2 v.2.2.5[61] with '--end-to-end' and '--very-sensitive' options, and a non-redundant training set of 700 high-confidence gene models with unambiguous start site positions was created based on the BLAST hits and RNA-seq coverage data. Non-redundancy of the training set was achieved by excluding genes with more than 70% identity at the amino acid level. Augustus annotation was further improved manually in several steps. First, transcribed ORFs >200 aa in length not predicted by Augustus were added to the genome annotation. Second, gene models with start sites predicted in regions with no transcriptomic coverage were corrected based on RNA-seq data and alignments with orthologous genes from other trypanosomatids. Orthologs of *Leptomonas* genes with uncertain start codons were extracted from TriTrypDB v. 7.0 and aligned using Muscle with the option '-maxiters 64'[63]. Finally, a frameshift correction step was performed using transcriptomic reads aligned to the *L. pyrrhocoris* genome as described above. When an insertion/deletion (indel) near a suspected frameshift site was supported by more than 50% of transcriptomic reads, it was introduced into the genome sequence, usually correcting a frameshift. For tRNA gene prediction the tRNAscan-SE program was used with default parameters[64]. For annotating other non-coding RNAs the BLASTN algorithm (E-value ≤ 10^{-10}) was employed with subsequent manual inspection of BLAST results. Non-coding RNAs (snRNA, snoRNA, rRNA, SRPRNA) of *C. fasciculata* and *Leishmania* spp. downloaded from TriTrypDB v.7

were used as BLAST query. As a result, 10,148 genes were annotated in the *L. pyrrhocoris* genome, which has been submitted to the TriTrypDB[65] and GenBank under the accession LGTL00000000 (BioProject PRJNA284491). The version described in this paper is version LGTL01000000.

Transcriptomic data processing and analysis

The following transcriptomic read libraries were generated for *L. pyrrhocoris* H10 and sequenced on the Illumina MiSeq platform: 1/ poly(A)-enriched RNA fraction, 17.3 million reads, 150 nt read length, insert size range 180-380 bp; 2/ total RNA fraction (rRNA partially depleted with the RiboMinus Eukaryote Kit for RNA-Seq, Life Technologies), 34.2 million reads, 250 nt read length, insert size range 200-400 bp.

Differential gene expression analysis was done using the RNA-seq tool in CLC Genomics Workbench v. 7.0 and 8.0. Raw reads were subjected to quality-based trimming (regions with Phred quality < 20 were trimmed, no more than one N was allowed in the remaining sequence), adapter trimming, and a minimum length threshold of 30 bp. Processed reads were then mapped to the annotated *L. mexicana*, *L. major*, or *L. seymouri* genomes with the following parameters: maximum number of mismatches, 2; minimum fraction of read length mapped, 0.9 ; minimum identity within the mapped sequence, 0.95 ; maximum number of best-scoring hits for a read, 30. All libraries were mapped as paired-end, and expression values (RPKM) for each gene were calculated. To identify gene sets that are differentially expressed between various conditions or life cycle stages, the EDGE test was employed[66]. Genes with expression fold change ≥ 1.5 and FDR-corrected p-value ≤ 0.05 were chosen for further analyses.

Identification of *trans*-splicing and polyadenylation acceptor sites

The paired end reads generated from both cDNA libraries were processed as described before[67]. Briefly, to identify SL acceptor sites reads from the RNA-seq library containing the final 12 bases (TGTACTTTATTG) of the SL sequence were extracted, the SL excised, and the remaining read mapped to the *L. pyrrhocoris* genome to record the positions of the *trans*-splice sites. Reads that were less than 21 nucleotides in length after extraction of the SL sequence were discarded prior to mapping. Reads that mapped to the unprocessed SL gene array were also discarded. To identify polyadenylation sites reads from the RNA-seq library were searched for runs of 5 or more A nucleotides at end of a read (or 5 or more T at the start). These A or T tails were removed from the read and the remainder of the read was mapped on the genome. The polyadenylation addition sites were recorded if the genomic locus itself contained no equivalent run of As or Ts at the mapped position.

Supplementary references

1 Murungi, E. *et al.* A comparative analysis of trypanosomatid SNARE proteins. *Parasitol Int* **63**, 341-348 (2014).

2 Dan-Goor, M., Nasereddin, A., Jaber, H. & Jaffe, C. L. Identification of a secreted casein kinase 1 in *Leishmania donovani*: effect of protein over expression on parasite growth and virulence. *PLoS One* **8**, e79287 (2013).

3 Buxbaum, L. U. *et al.* Cysteine protease B of *Leishmania mexicana* inhibits host Th1 responses and protective immunity. *J Immunol* **171**, 3711-3717 (2003).

4 Williams, R. A., Woods, K. L., Juliano, L., Mottram, J. C. & Coombs, G. H. Characterization of unusual families of ATG8-like proteins and ATG12 in the protozoan parasite *Leishmania major*. *Autophagy* **5**, 159-172 (2009).

5 Papadopoulou, B. *et al.* Reduced infectivity of a *Leishmania donovani* biopterin transporter genetic mutant and its use as an attenuated strain for vaccination. *Infect Immun* **70**, 62-68 (2002).

6 McCall, L. I. & McKerrow, J. H. Determinants of disease phenotype in trypanosomatid parasites. *Trends Parasitol* **30**, 342-349 (2014).

7 Ghedin, E. *et al.* Gene synteny and evolution of genome architecture in trypanosomatids. *Mol Biochem Parasitol* **134**, 183-191 (2004).

8 Soderlund, C., Nelson, W., Shoemaker, A. & Paterson, A. SyMAP: A system for discovering and viewing syntenic regions of FPC maps. *Genome Res* **16**, 1159-1168 (2006).

9 Peacock, C. S. *et al.* Comparative genomic analysis of three *Leishmania* species that cause diverse human disease. *Nat Genet* **39**, 839-847 (2007).

10 Porcel, B. M. *et al.* The streamlined genome of *Phytomonas* spp. relative to human pathogenic kinetoplastids reveals a parasite tailored for plants. *PLOS Genet* **10**, e1004007 (2014).

11 Carver, T., Harris, S. R., Berriman, M., Parkhill, J. & McQuillan, J. A. Artemis: an integrated platform for visualization and analysis of high-throughput sequence-based experimental data. *Bioinformatics* **28**, 464-469 (2012).

12 Darling, T. N. & Blum, J. J. D-lactate production by *Leishmania braziliensis* through the glyoxalase pathway. *Mol Biochem Parasitol* **28**, 121-127 (1988).

13 Michels, P. A., Bringaud, F., Herman, M. & Hannaert, V. Metabolic functions of glycosomes in trypanosomatids. *Biochim Biophys Acta* **1763**, 1463-1477 (2006).

14 Contreras-Shannon, V. & McAlister-Henn, L. Influence of compartmental localization on the function of yeast NADP+-specific isocitrate dehydrogenases. *Arch Biochem Biophys* **423**, 235-246 (2004).

15 van Weelden, S. W., van Hellemond, J. J., Opperdoes, F. R. & Tielens, A. G. New functions for parts of the Krebs cycle in procyclic *Trypanosoma brucei*, a cycle not operating as a cycle. *J Biol Chem* **280**, 12451-12460 (2005).

16 van Hellemond, J. J., Opperdoes, F. R. & Tielens, A. G. The extraordinary mitochondrion and unusual citric acid cycle in *Trypanosoma brucei*. *Biochem Soc Trans* **33**, 967-971 (2005).

17 Gutteridge, W. E. Some effects of pentamidine di-isethionate on *Crithidia fasciculata*. *J Protozool* **16**, 306-311 (1969).

18 Bringaud, F., Barrett, M. P. & Zilberstein, D. Multiple roles of proline transport and metabolism in trypanosomatids. *Front Biosci* **17**, 349-374 (2012).

19 Barderi, P. *et al.* The NADP+-linked glutamate dehydrogenase from *Trypanosoma cruzi*: sequence, genomic organization and expression. *Biochem J* **330 (Pt 2)**, 951-958 (1998).

20 Olin-Sandoval, V., Moreno-Sanchez, R. & Saavedra, E. Targeting trypanothione metabolism in trypanosomatid human parasites. *Curr Drug Targets* **11**, 1614-1630 (2010).

21 Eeckhout, Y. [Properties and location of the Trypanosomide "*Crithidia luciliae*" acid hydrolases]. *Arch Int Physiol Biochim* **78**, 993-994 (1970).

22 Souto-Padron, T. & de Souza, W. Fine structure and cytochemistry of peroxisomes (microbodies) *Leptomonas samueli*. *Cell Tissue Res* **222**, 153-158 (1982).

23 Field, M. C. & Carrington, M. The trypanosome flagellar pocket. *Nat Rev Microbiol* **7**, 775-786 (2009).

24 Berriman, M. *et al.* The genome of the African trypanosome *Trypanosoma brucei*. *Science* **309**,

416-422 (2005).

25 Leung, K. F., Dacks, J. B. & Field, M. C. Evolution of the multivesicular body ESCRT machinery; retention across the eukaryotic lineage. *Traffic* **9**, 1698-1716 (2008).

26 Koumandou, V. L., Dacks, J. B., Coulson, R. M. & Field, M. C. Control systems for membrane fusion in the ancestral eukaryote; evolution of tethering complexes and SM proteins. *BMC Evol Biol* **7**, 29 (2007).

27 Koumandou, V. L. *et al.* Evolutionary reconstruction of the retromer complex and its function in *Trypanosoma brucei*. *J Cell Sci* **124**, 1496-1509 (2011).

28 Adung'a, V. O., Gadelha, C. & Field, M. C. Proteomic analysis of clathrin interactions in trypanosomes reveals dynamic evolution of endocytosis. *Traffic* **14**, 440-457 (2013).

29 Hirst, J. *et al.* The fifth adaptor protein complex. *PLoS Biol* **9**, e1001170 (2011).

30 Hirst, J. *et al.* Characterization of TSET, an ancient and widespread membrane trafficking complex. *Elife* **3**, e02866 (2014).

31 Manna, P. T., Kelly, S. & Field, M. C. Adaptin evolution in kinetoplastids and emergence of the variant surface glycoprotein coat in African trypanosomatids. *Mol Phylogenet Evol* **67**, 123-128 (2013).

32 De Melo, L. D., Eisele, N., Nepomuceno-Silva, J. L. & Lopes, U. G. TcRho1, the *Trypanosoma cruzi* Rho homologue, regulates cell-adhesion properties: evidence for a conserved function. *Biochem Biophys Res Commun* **345**, 617-622 (2006).

33 Abbasi, K., DuBois, K. N., Dacks, J. B. & Field, M. C. A novel Rho-like protein TbRHP is involved in spindle formation and mitosis in trypanosomes. *PLoS One* **6**, e26890 (2011).

34 Price, H. P., Panethymitaki, C., Goulding, D. & Smith, D. F. Functional analysis of TbARL1, an N-myristoylated Golgi protein essential for viability in bloodstream trypanosomes. *J Cell Sci* **118**, 831-841 (2005).

35 Larkin, M. A. *et al.* Clustal W and Clustal X version 2.0. *Bioinformatics* **23**, 2947-2948 (2007).

36 Hall, T. A. BioEdit: a user-friendly biological sequence alignment editor and analysis program for Windows 95/98/NT. *Nucl Acids Symp Ser* **41**, 95-98 (1999).

37 Guindon, S. *et al.* New algorithms and methods to estimate maximum-likelihood phylogenies: assessing the performance of PhyML 3.0. *Syst Biol* **59**, 307-321 (2010).

38 Ronquist, F. *et al.* MrBayes 3.2: efficient Bayesian phylogenetic inference and model choice across a large model space. *Syst Biol* **61**, 539–542 (2012).

39 Goldberg, J. M. *et al.* Kinannote, a computer program to identify and classify members of the eukaryotic protein kinase superfamily. *Bioinformatics* **29**, 2387-2394 (2013).

40 Parsons, M., Worthey, E. A., Ward, P. N. & Mottram, J. C. Comparative analysis of the kinomes of three pathogenic trypanosomatids: *Leishmania major*, *Trypanosoma brucei* and *Trypanosoma cruzi*. *BMC Genomics* **6**, 127 (2005).

41 Jones, N. G. *et al.* Regulators of *Trypanosoma brucei* cell cycle progression and differentiation identified using a kinome-wide RNAi screen. *PLoS Pathog* **10**, e1003886 (2014).

42 Hem, S. *et al.* Identification of *Leishmania*-specific protein phosphorylation sites by LC-ESI-MS/MS and comparative genomics analyses. *Proteomics* **10**, 3868-3883 (2010).

43 Urbaniak, M. D., Martin, D. M. & Ferguson, M. A. Global quantitative SILAC phosphoproteomics reveals differential phosphorylation is widespread between the procyclic and bloodstream form lifecycle stages of *Trypanosoma brucei*. *J Proteome Res* **12**, 2233-2244 (2013).

44 Silva-Almeida, M., Pereira, B. A., Ribeiro-Guimaraes, M. L. & Alves, C. R. Proteinases as virulence factors in *Leishmania* spp. infection in mammals. *Parasit Vectors* **5**, 160 (2012).

45 Sádlová, J., Volf, P., Victoir, K., Dujardin, J. C. & Votýpka, J. Virulent and attenuated lines of *Leishmania major*: DNA karyotypes and differences in metalloproteinase GP63. *Folia Parasitol* **53**, 81-90 (2006).

46 Joshi, P. B., Kelly, B. L., Kamhawi, S., Sacks, D. L. & McMaster, W. R. Targeted gene deletion in *Leishmania major* identifies leishmanolysin (GP63) as a virulence factor. *Mol Biochem Parasitol* **120**, 33-40 (2002).

47 Hajmová, M., Chang, K. P., Kolli, B. & Volf, P. Down-regulation of gp63 in *Leishmania amazonensis* reduces its early development in *Lutzomyia longipalpis*. *Microbes Infect* **6**, 646-649 (2004).

48 Szöör, B. Trypanosomatid protein phosphatases. *Mol Biochem Parasitol* **173**, 53-63 (2010).

49 Williams, R. A., Mottram, J. C. & Coombs, G. H. Distinct roles in autophagy and importance in infectivity of the two ATG4 cysteine peptidases of *Leishmania major*. *J Biol Chem* **288**, 3678-3690 (2013).

50 Svárovská, A. *et al. Leishmania major* glycosylation mutants require phosphoglycans (lpg2-) but not lipophosphoglycan (lpg1-) for survival in permissive sand fly vectors. *PLoS Negl Trop Dis* **4**, e580 (2010).

51 Klein, C., Gopfert, U., Goehring, N., Stierhof, Y. D. & Ilg, T. Proteophosphoglycans of *Leishmania mexicana*. Identification, purification, structural and ultrastructural characterization of the secreted promastigote proteophosphoglycan pPPG2, a stage-specific glycoisoform of amastigote aPPG. *Biochem J* **344 Pt 3**, 775-786 (1999).

52 Mukhopadhyay, S. & Mandal, C. Glycobiology of *Leishmania donovani*. *Indian J Med Res* **123**, 203-220 (2006).

53 Guha-Niyogi, A., Sullivan, D. R. & Turco, S. J. Glycoconjugate structures of parasitic protozoa. *Glycobiology* **11**, 45R-59R (2001).

54 Opperdoes, F. & Michels, P. A. in *Leishmania: after the genome* (eds P. Myler & N. Fasel) Ch. 7, 123-158 (Caister Academic Press, 2008).

55 Lee, S. H., Stephens, J. L. & Englund, P. T. A fatty-acid synthesis mechanism specialized for parasitism. *Nat Rev Microbiol* **5**, 287-297 (2007).

56 Ghosh, S., Banerjee, P., Sarkar, A., Datta, S. & Chatterjee, M. Coinfection of *Leptomonas seymouri* and *Leishmania donovani* in Indian leishmaniasis. *J Clin Microbiol* **50**, 2774-2778 (2012).

57 Singh, N., Chikara, S. & Sundar, S. SOLiD sequencing of genomes of clinical isolates of *Leishmania donovani* from India confirm *Leptomonas* co-infection and raise some key questions. *PLOS One* **8**, e55738 (2013).

58 Wertlieb, D. M. & Guttman, H. N. Catalase in insect trypanosomatids. *J Protozool* **10**, 109-112 (1963).

59 Isaza, C. E. *et al.* A proposed role for *Leishmania major* carboxypeptidase in peptide

catabolism. *Biochem Biophys Res Commun* **373**, 25-29 (2008).

60 Alves, J. M. *et al.* Endosymbiosis in trypanosomatids: the genomic cooperation between bacterium and host in the synthesis of essential amino acids is heavily influenced by multiple horizontal gene transfers. *BMC Evol Biol* **13**, 190 (2013).

61 Langmead, B. & Salzberg, S. L. Fast gapped-read alignment with Bowtie 2. *Nat Methods* **9**, 357-359 (2012).

62 Stanke, M. *et al.* AUGUSTUS: *ab initio* prediction of alternative transcripts. *Nucleic Acids Res* **34**, W435-439 (2006).

63 Edgar, R. C. MUSCLE: multiple sequence alignment with high accuracy and high throughput. *Nucleic Acids Res* **32**, 1792-1797 (2004).

64 Lowe, T. M. & Eddy, S. R. tRNAscan-SE: a program for improved detection of transfer RNA genes in genomic sequence. *Nucleic Acids Res* **25**, 955-964 (1997).

65 Aslett, M. *et al.* TriTrypDB: a functional genomic resource for the Trypanosomatidae. *Nucleic Acids Res* **38**, D457-462 (2010).

66 Si, Y. & Liu, P. An optimal test with maximum average power while controlling FDR with application to RNA-seq data. *Biometrics* **69**, 594-605 (2013).

67 Fiebig, M., Gluenz, E., Carrington, M. & Kelly, S. SLaP mapper: a webserver for identifying and quantifying spliced-leader addition and polyadenylation site usage in kinetoplastid genomes. *Mol Biochem Parasitol* **196**, 71-74 (2014).
